# Morphological and Phylogenetic Characterizations Reveal Five New Species of *Astrothelium* (*Trypetheliales*, *Ascomycota*) from China

**DOI:** 10.3390/jof8100994

**Published:** 2022-09-22

**Authors:** Shu-Hua Jiang, Chao Zhang, Xian-Dong Xue, André Aptroot, Jiang-Chun Wei, Xin-Li Wei

**Affiliations:** 1 State Key Laboratory of Mycology, Institute of Microbiology, Chinese Academy of Sciences, Beijing 100101, China; 2 Institute of Life Science and Green Development, College of Life Sciences, Hebei University, Baoding 071002, China; 3 College of Life Sciences, Shandong Normal University, Jinan 250014, China; 4 Laboratório de Botânica/Liquenologia, Instituto de Biociências, Universidade Federal de Mato Grosso do Sul, Avenida Costa e Silva s/n, Bairro Universitário, Campo Grande CEP 79070-900, Mato Grosso do Sul, Brazil; 5 University of Chinese Academy of Sciences, Beijing 100049, China

**Keywords:** diversity, morphology, new taxa, *Trypetheliaceae*, phylogeny

## Abstract

The lichenized fungal genus *Astrothelium* is an important element of crustose lichen communities in tropical to subtropical forests. Morphological and molecular phylogenetic approaches to investigate species diversity of *Astrothelium* (*Trypetheliaceae*) from Southern China were carried out in this study. Bayesian and maximum-likelihood (ML) analyses were generated based on the combined data set of internal transcribed spacer (ITS), partial regions of the nuclear ribosomal large subunit (LSU), and the largest subunit of RNA polymerase II gene sequences (RPB1). The morphological comparison with the known *Astrothelium* taxa and molecular phylogeny support five new species: *Astrothelium jiangxiense* sp. nov., *A. luminothallinum* sp. nov., *A. pseudocrassum* sp. nov., *A. subeustominspersum* sp. nov., and *A. subrufescens* sp. nov. All these species are described and illustrated in detail.

## 1. Introduction

*Astrothelium* Eschw. is a genus of lichenized fungi, with the type species *Astrothelium conicum* Eschw., belonging to the family *Trypetheliaceae* in the order *Trypetheliales* in the class *Dothideomycetes* of the phylum *Ascomycota* [1,2,3]. Traditionally, *Astrothelium* included the species with fused lateral ostioles and transversely septate ascospores. Harris (1989, 1995) anticipated that the classification scheme employed for *Trypetheliaceae* was artificial and would result in the polyphyly of some genera [4,5]. Aptroot et al. (2008) subsequently echoed his contentions, further emphasizing the need for a revision of generic concepts within *Trypetheliaceae* [6]. Utilizing molecular data, Del Prado et al. (2006) and Nelsen et al. (2009) began assessing Harris’s assertions and demonstrated the non-monophyly of *Trypethelium* and *Astrothelium* [3,7]. Further, ontogenetic studies of muriform-spored taxa revealed these spores initially form transverse septa with diamond-shaped lumina and subsequently develop muriform septation, thus suggesting a close evolutionary connection between species producing these different ascospore types [8]. Nelsen et al. (2014) explicitly studied relationships within *Trypetheliaceae* based on molecular analysis and showed that species from a number of genera together form a strongly supported group, referred to as the “Astrothelium” clade [9]. This result was supported by Lücking et al. [10,11] and Aptroot and Lücking [12]. With its modern circumscription [12], the genus, with a total of over 250 taxa [1,13,14,15,16,17], is the largest in the family and exhibits much variation in perithecial arrangement and ascospore septation, including species essentially referred to as the previously separate genera *Astrothelium*, *Bathelium*, *Campylothelium*, *Cryptothelium*, *Laurera*, and *Trypethelium* [1,2,9,10,12,13,14,15]. In both its original and revised delimitation, the genus has a pantropical distribution [1,2,15,18,19,20,21].

*Astrothelium* is distinguished by the corticate, usually olive-green thallus, the simple to aggregated or pseudostromatic ascomata with apical to lateral, separate or fused ostioles; the ascomata or pseudostromata can be immersed to prominent, often different from the thallus in structure and color. The ascospores in *Astrothelium* are distoseptate, with diamond-shaped lumina, which are best seen in the species with transversely septate ascospores, but ascospores with muriform septation are also common [1,12]. Thus far, most known species in the genus produce hyaline ascospores, except *Astrothelium fuscosporum* Soto-Medina, Aptroot and Lücking, producing very characteristic, multiseptate, and brown ascospores [12,22].

*Astrothelium* species with astrothelioid ascospores in China are poorly known; up to now, only four species have been recorded: *A. cinnamomeum* (Eschw.) Müll. Arg. [23], *A. sinense* S.H. Jiang and C. Zhang [17], *A. speciosum* Zahlbr. [24], and *A. variolosum* (Ach.) Müll. Arg. [24]. The aim of this study is to investigate the diversity of *Astrothelium* in Southern China, combining morphological, chemical, and molecular data.

## 2. Materials and Methods

### 2.1. Material Examined

All materials were collected from Guangdong, Guangxi, Guizhou, and Jiangxi Provinces, and are preserved in the Herbarium Mycologicum Academiae Sinicae-Lichenes, Beijing, China (HMAS–L) and Fungarium of the College of Life Sciences, Liaocheng University, Liaocheng, China (LCUF).

### 2.2. Phenotypic Analysis

Morphological characters were examined and photographed under a Motic SMZ-168 series stereo microscope and LEICA M125 and DFC450 dissecting microscopes. The anatomical characters were explored with a razor blade under an A2 dissecting stereoscope with an Axio Imager.

### 2.3. Chemical Analysis

The lichen substances were detected using thin layer chromatography (TLC) [25,26]. The particularly stable and reliable solvent C (toluene/acetic acid 170:30) was used in this study as it often provides the best discrimination of lichen substances [25]. Relative Rf values were determined on the plate by a control mixture: atranorin and norstictic acid, in *Lethariella cladonioides* (Nyl.) Krog. The controls were assigned invariant Rf values, and other spots were measured relative to them.

We utilized the normal procedure—to soak the lichen fragments firstly in c. 1 mL of acetone for 10 min in a small test tube. Then, this solution was used for spotting on the TLC plate. After that, the plate was preequilibrated with glacial acetic acid vapor and subsequently proceeded with elution in solvent C. The plate was dried and then examined under short wavelength (254 nm) ultraviolet light for pigments. Further, it was sprayed with 10% sulfuric acid and heated at 110° in an oven for 10 min to develop the spots. The Rf values and color of each lichen substance were recorded and immediately examined under long wavelength (365 nm) ultraviolet light. The Rf values, as well as the fluorescent properties, were compared and analyzed to confirm the identity of the substance [25,26].

### 2.4. Phylogenetic Analyses

#### 2.4.1. DNA Extraction and PCR Amplification

Sixteen fresh specimens were chosen for DNA extraction (Supplementary file S1) using the modified CTAB method [27]. The partial region of the internal transcribed spacer (ITS) was amplified using the ITS4 and ITS5 primers [28]. The fungal nuclear ribosomal large subunit (LSU) was amplified using combinations of the primers: LROR-ACCCGCTGAACTTAAGC [29], LR3-GGTCCGTGTTTCAAGAC [29], 1F-CAGTCTGAGTGAATTGCTAA (in this study), and 1R-TTTCTTGACATTGGCATTTG (in this study). The largest subunit of RNA polymerase II gene sequence RPB1 was amplified using the primers RPB1-Af and RPB1-Cr [29].

PCR reactions were carried out in 25 μL containing 1 µL each primer solution (10 µM), 2 µL genomic DNA, 8 µL ddH_2_O, and 13 µL 2 × Taq PCR MasterMix^®^ (Cwbio Inc., Jiangsu, China). Thermocycling of ITS conditions comprised initial denaturation at 95 °C for 5 min; followed by 31 cycles of denaturation at 94 °C for 30 s, annealing at 52 °C for 30 s, elongation at 72 °C for 50 s, and a final extension at 72 °C for 10 min. PCR amplification of LSU and RPB1 included: a 1 min initial denaturation at 94 °C, 38 cycles of 1 min denaturation at 94 °C, 45 s (for LSU) or 90 s (for RPB1) annealing step at 52 °C, 1 min extension at 72 °C, a final extension at 72 °C for 10 min. The target product of PCR was checked by 0.8% agarose electrophoresis gels and sequenced by Majorbio Sanger Inc. (Beijing, China). The new sequences derived in this study were deposited in GenBank (https://www.ncbi.nlm.nih.gov/, accessed on 25 August 2022; Supplementary file S1).

#### 2.4.2. Phylogenetic Analyses

Sequences for each marker in this study were combined with those obtained from GenBank by Basic Local Alignment Search Tool (BLAST) (Supplementary file S1), generating a separate ITS and a concatenated three-locus (ITS, LSU, RPB1) dataset (Supplementary files S2 and S3). The top hits obtained after running a BLAST search of ITS were also included (Supplementary file S4), which can help us judge the novelty of species more easily based on quantitative measurement. Compared to ITS, LSU is relatively conservative, and difficult to distinguish species when they are closely related. However, when the two independent branches in the LSU tree were estimated for large evolutionary divergence, it also indicates that the two species are distinct. Therefore, considering that most of the known sequences of *Astrothelium* are about LSU, we generated a separate LSU dataset for analysis (Supplementary file S5). Estimates of evolutionary divergence between LSU sequences were conducted in MEGA v.7 (Kumar et al., Philadelphia, PA, USA) [30].

For constructing the phylogenetic tree, the genus *Bathelium*, belonging to the same family *Trypetheliaceae* within *Astrothelium*, was chosen as outgroup [10]. Sequences for each marker were firstly aligned independently with MAFFT v.7 (Katoh and Standley, Osaka, Japan) [31], and the combinability was tested as described previously [32]. Only when no significant conflict was detected, would the three markers ITS, LSU, and RPB1 be combined. Bayesian analyses were performed by using MrBayes v.3.2.7 (Ronquist et al., Stockholm, Sweden), as detailed in Jiang et al. [32]. Every 100th generation was sampled as a tree with 5,000,000 generations running. Maximum likelihood (ML) analyses involving 1000 pseudoreplicates were performed using IQ-TREE v2.0.6 (Minh et al., Canberra, Australia) [33]. The best-fit substitution model was selected using ModelFinder [34]. In the ML analyses of ITS sequences, the TIM2 + F + G4 model was selected as the best model according to BIC. TN + F + I + G4 and TIM2 + F + I + G4 were selected as the best models for LSU and the three-locus dataset, respectively. The phylogenetic tree was drawn by FigTree v.1.4.3 (http://tree.bio.ed.ac.uk/software/figtree/, accessed on 25 August 2022).

## 3. Results

### 3.1. Phylogenetic Analyses

**Figure 1:**
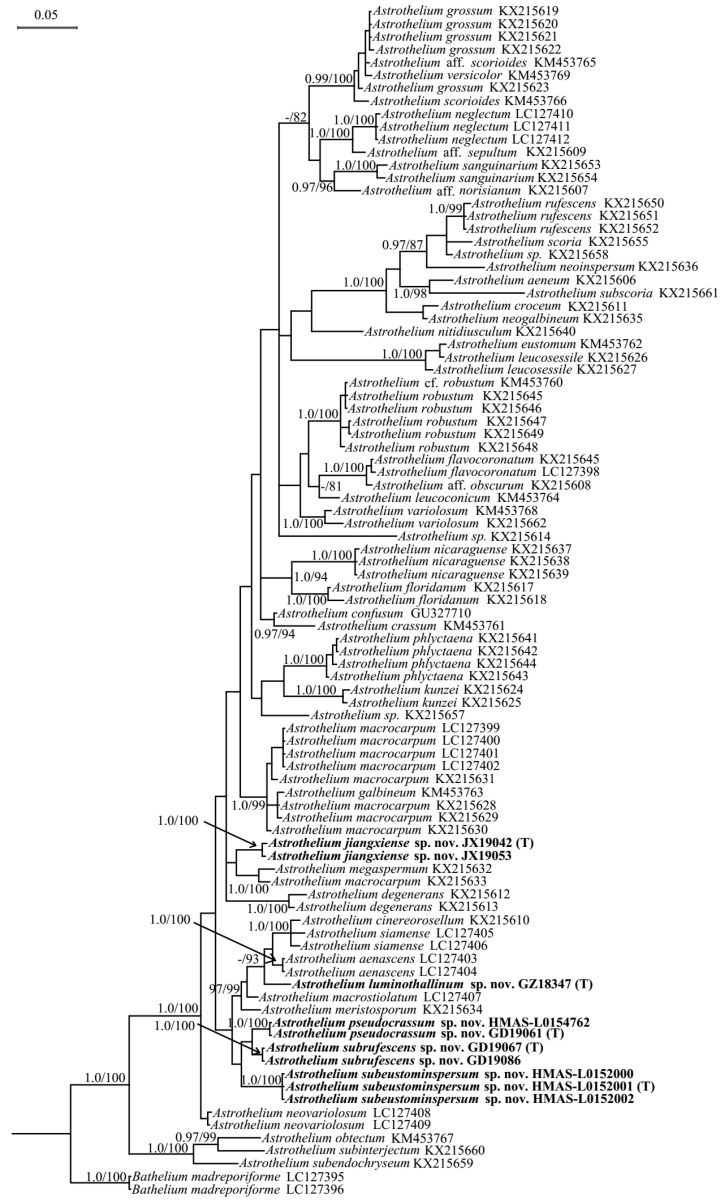
Bayesian tree showing the internal phylogeny of *Astrothelium* based on LSU (587 bp). Bayesian inference posterior probabilities above 0.95 (left) and maximum likelihood bootstrap support above 70% (right) are shown at nodes (B–PP/ML–BP). Terminals in boldface indicate newly generated sequences for this study. The type specimens in the phylogenetic tree were labeled by letter T.

**Figure 2:**
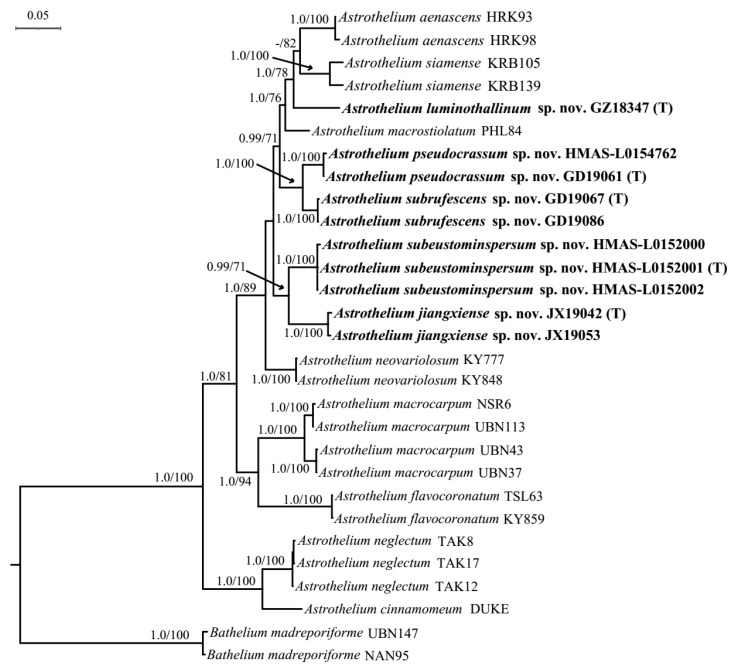
Bayesian tree showing the internal phylogeny of *Astrothelium* based on three markers (ITS, LSU, and RPB1) with an alignment length of 1880 bp. Bayesian inference posterior probabilities above 0.95 (left) and maximum likelihood bootstrap support above 70% (right) are shown at nodes (B–PP/ML–BP). Terminals in boldface indicate newly generated sequences for this study. The type specimens in the phylogenetic tree were labeled by letter T.

The dataset included 16 ITS sequences, 10 LSU sequences, and 7 RPB1 sequences newly generated in this study. The ITS (Supplementary file S6) and LSU sequences were analyzed separately (Figure 1) and subsequently compared with the three-marker tree based on a concatenated alignment with 1880 bp (ITS: 455 bp; LSU: 587 bp; RPB1: 838 bp; Figure 2). No different relationships were revealed by the separate analyses for the ITS, LSU, and RPB1 datasets, all with reciprocal posterior probabilities (PP) of 0.99; therefore, these three markers can be combined.

As we know, ITS was often used as barcoding to distinguish lichen species due to its high variability [35]. Compared to ITS, LSU is relatively conservative, and its sensitivity is lower at the species level. Thus, LSU is difficult to distinguish species with when they are closely related and similar, but when the two independent lineages in the LSU tree were estimated of large evolutionary divergence (Supplementary file S7), it can help us delimit them into different species.

The single ITS and LSU phylogeny and the combined sequence matrices revealed five new monophyletic lineages corresponding to five new species here: *Astrothelium jiangxiense* S.H. Jiang and C. Zhang sp. nov., *A. luminothallinum* S.H. Jiang and C. Zhang sp. nov., *A. pseudocrassum* S.H. Jiang and C. Zhang sp. nov., *A. subeustominspersum* S.H. Jiang and C. Zhang sp. nov., and *A. subrufescens* S.H. Jiang and C. Zhang sp. nov. Most clades in all trees had strong support through Bayesian and ML analyses. *Astrothelium* *luminothallinum* is such a unique species that no close relatives were found in all trees (Figure 1 and Figure 2, and Supplementary file S6); they all supported it as a distinct clade. In addition, *A. pseudocrassum* clustered with *A. subrufescens* (Figure 1 and Figure 2, and Supplementary file 6), but they can be distinguished easily in morphology. *A. jiangxiense* and *A.* *subeustominspersum* were grouped into one clade according to the three-gene combined tree (Figure 2). However, the single ITS and LSU phylogenetic trees did not support this relationship, showing that each species was highly supported and obviously separated from the others (Figure 1 and Supplementary file S6). This minor conflict makes it challenging to evaluate the exact relationships between different clades in *Astrothelium*; however, it does not affect their interpretation as distinct phylogenetic entities.

### 3.2. Taxonomy

**Figure 3:**
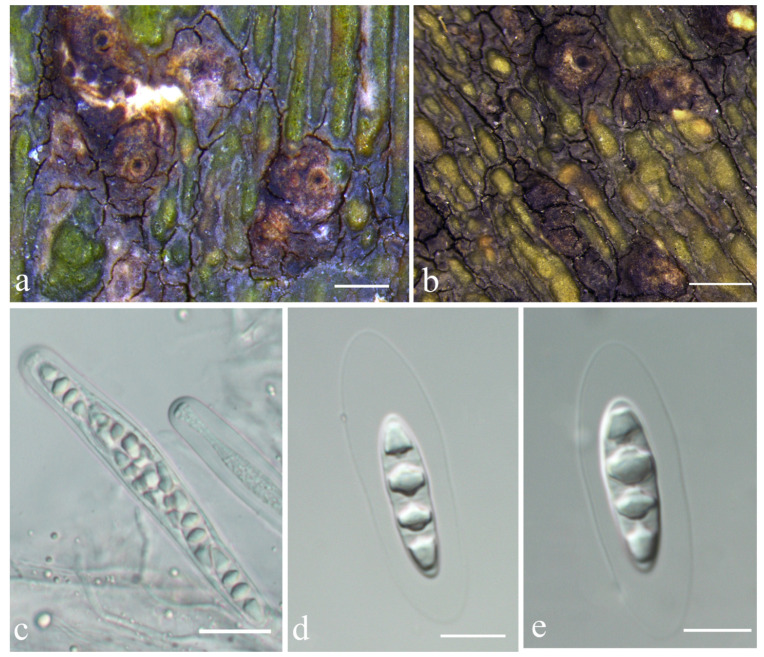
*Astrothelium jiangxiense* sp. nov. (**a**,**b**) Thallus with ascomata (holotype, LCUF JX19042); (**c**) ascus, with hamathecium interspersed with oil droplets (LCUF JX19053); (**d**,**e**) ascospores (holotype, LCUF JX19042). Scale bars: (**a**,**b**) = 0.1 mm; (**c**) = 20 µm; (**d**,**e**) = 10 µm.

***Astrothelium jiangxiense*** S.H. Jiang & C. Zhang, sp. nov. (Figure 3)

Fungal Names FN 571292.

*Etymology*: The epithet refers to the discovery of this new species in Jiangxi province, China.

*Typus*: CHINA, Jiangxi, Shangrao City, Yushan County, Sanqing Mountain, alt. 1295 m, on bark, 17 March 2019, M. Li JX19042 (LCUF, holotype).

*Diagnosis*: The new species differs from the similar species *Astrothelium scoria* (Fée) Aptroot and Lücking and *A. subscoria* Flakus and Aptroot by the bullate thallus and usually single but not white-covered pseudostromata.

*Description*: Thallus crustose, corticolous, olive-green to bright green, verrucose to bullate, continuous, without prothallus, 40–90 μm thick, covering areas up to 5 cm diam. Algae trentepohlioid. Ascomata perithecia, conical to pyriform, black, 0.6–1 mm diam., erumpent to prominent, usually single, but sometimes 2–4 aggregated in pseudostromata. Pseudostromata blackish brown. Ostiole apical, black, except for a pale brown rim around 50–100 μm diam. Ascomata wall carbonized, black, 40–260 μm thick. Hamathecium hyaline, interspersed with oil droplets. Asci cylindrical to clavate, 120–140 × 12–15 µm. Ascospores eight per ascus, biseriate, hyaline, fusiform, transversely three-septate, 16–30 × 6–8 μm, lumina diamond-shaped, surrounded by a smooth gelatinous sheath, 2–8 μm wide. Pycnidia not seen.

*Chemistry*: Thallus UV-, pseudostromata UV-. TLC showed an unidentified substance at Rf five of solvent C, with UV+ red reaction (Supplementary file S8).

*Habitat and distribution*: The new species grows on the bark of tropical and subtropical regions and is currently only found in China.

*Additional specimens examined*: CHINA. Jiangxi, Shangrao City, Yushan County, Sanqing Mountain, alt. 1317 m, on bark, 17 March 2019, Z.T. Yao JX19053 (LCUF).

*Notes*: This taxon can be recognized by its olive-green to bright green bullate thallus (UV-), pale yellow to brown, somewhat conical pseudostromata, usually single ascomata, and apical ostiole. In TLC examination, we found an unknown substance at Rf five of solvent C, UV+ red (Supplementary file S8). It would key out in the recent world key [36] in key I at couplet 5 and 6 (Supplementary file S9) and differs by the bullate thallus and usually single but not white-covered pseudostromata. *Astrotheliumscoria* resembles the new species in ascospore characters; however, its thallus is often yellowish or brownish and smooth; besides, ascomata are irregularly grouped to pseudostromata, and no substances can be detected by TLC [12]. Further, the new species and *A. scoria* form independent branches in the phylogenetic tree constructed by LSU (Figure 1) and show large evolutionary divergence (15.4%, Supplementary file S7), which supports them as different species. *A. subscoria* is another similar species but can be distinguished by ascomata with white cover [37]. In addition, the LSU analysis indicates their large genetic distance (16.7%; Figure 1, Supplementary file S7). It is also somewhat similar to *A. aenascens* Aptroot, but the latter has parietin detected by TLC, and ascospores are not surrounded by a gelatinous layer [38]. Furthermore, the molecular evidence support that they are two distinct species (Figure 1 and Figure 2, Supplementary file S6). *A. diaphanocorticatum* Aptroot and Sipmanis is similar to the new species in the bullate thallus and three-septate ascospores, but its asci are wider (120–150 × 16–20 μm) and ascospores are not surrounded by a gelatinous layer [16].

**Figure 4:**
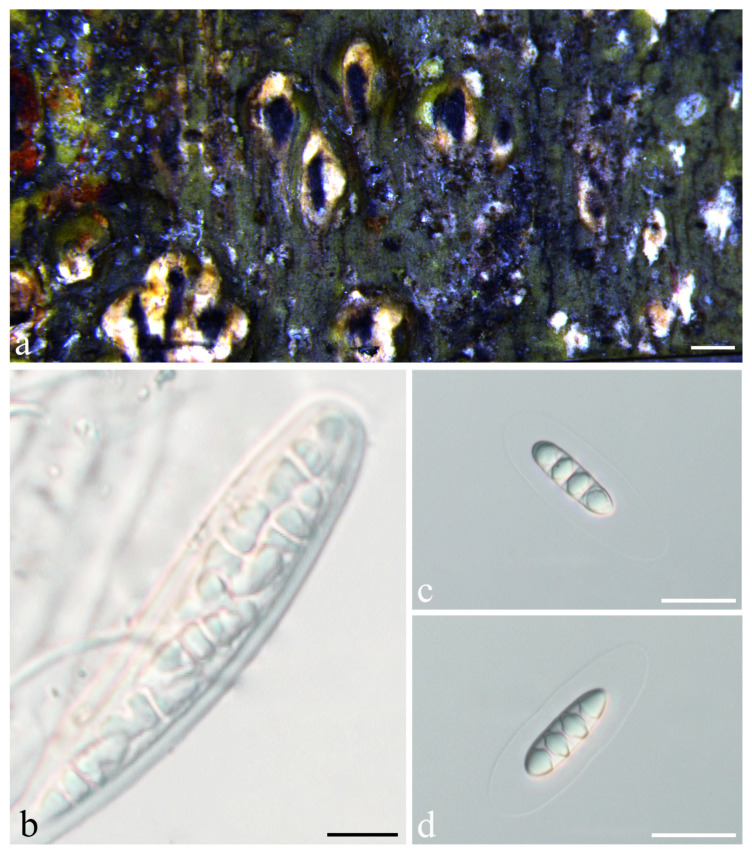
*Astrothelium luminothallinum* sp. nov. (holotype, LCUF GZ18347). (**a**) Thallus and pseudostromata; (**b**) ascus; (**c**,**d**) ascospores. Scale bars: (**a**) = 0.25 mm; (**b**) = 20 µm; (**c**,**d**) = 20 µm.

***Astrothelium luminothallinum*** S.H. Jiang and C. Zhang, sp. nov. (Figure 4)

Fungal Names FN 571293.

*Etymology*: The epithet refers to the thallus containing lichexanthone, contrasting with the pseudostromata, which are absent of lichexanthone.

*Typus*: CHINA, Guizhou, Libo County, Limingguanxiang, alt. 750 m, on bark, 23 October 2018, Z.F. Jia GZ18347 (LCUF, holotype).

*Diagnosis*: The new species was similar to *Astrothelium phlyctaena* (Fée) Aptroot and Lücking but can be distinguished by interspersed hamathecium, and UV-negative pseudostromata.

*Description*: Thallus crustose, corticolous, continuous, grey-green or light green, 2–4 cm diam., 300–600 µm thick, smooth to uneven. Algae trentepohlioid. Ascomata perithecia, conical to pyriform, elongated, black, 0.15–0.40 mm diam., immersed to erumpent, single or aggregated in pseudostromata. Pseudostromata surfaces grey-white or grey-yellow, not concolorous to the thallus, raised, top flattened. Ostiole apical, appearing as blackish dots, black, 0.1–0.3 mm diam. Ascomata wall carbonized, black, 32–72 μm thick. Hamathecium hyaline, interspersed with oil droplets. Asci cylindrical to clavate, colorless, 75–120 × 12–18 µm. Ascospores eight per ascus, biseriate to irregular, hyaline, fusiform, ends rounded, transversely three-septate, 18–29 × 6–10 μm, lumina diamond-shaped, surrounded by a smooth gelatinous sheath, 4–18 μm wide. Pycnidia not seen.

*Chemistry*: Thallus UV+ yellow, pseudostromata UV-. TLC: lichexanthone.

*Habitat and distribution*: The new species grows on the bark of tropical and subtropical regions and is currently only found in China.

*Additional specimens examined*: CHINA, Guizhou, Libo County, Limingguanxiang, alt. 750 m, on bark, 23 October 2018, Z.F. Jia 20191092 (HMAS-L 0154761).

*Notes*: This new species would key out in the recent world key [36] in key H at couplet 16 and 17 (Supplementary file S9) and is similar to *A. phlyctaena*, but the latter often has clear hamathecium and pseudostromata UV+ yellow [12]. In addition, they formed independent branches in the LSU phylogenetic tree, with large evolutionary divergence (10.2%; Figure 1, Supplementary file S7). Another related species is *A. leucosessile* Lücking, M.P. Nelsen and Aptroot, but it differs by having whitish and sessile pseudostromata [11]. Further, the LSU analysis reveals its large genetic distance from *A. luminothallinum* (14.3%, Supplementary file S7, Figure 1).

**Figure 5:**
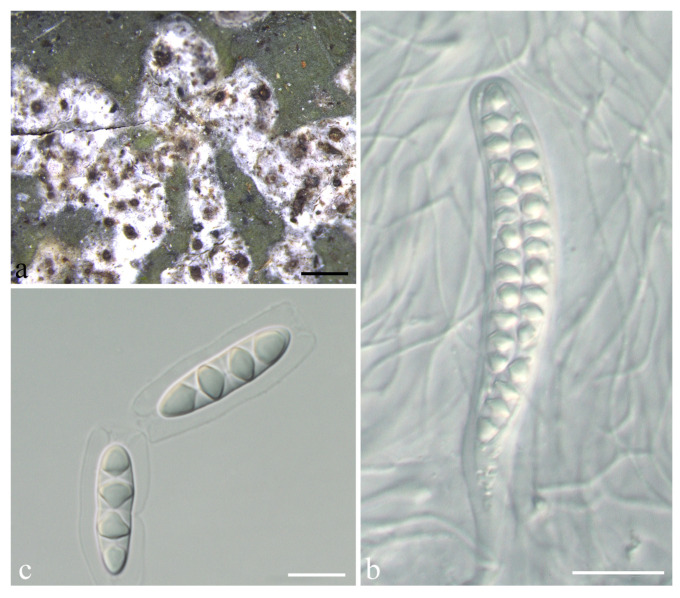
*Astrothelium pseudocrassum* sp. nov. (holotype, LCUF GD19061). (**a**) Thallus with ascomata; (**b**) ascus; (**c**) ascospores. Scale bars: (**a**) = 1 mm; (**b**) = 20 μm; (**c**) = 10 µm.

***Astrothelium pseudocrassum*** S.H. Jiang and C. Zhang, sp. nov. (Figure 5)

Fungal Names FN 571294.

*Etymology*: The epithet conveys the potential confusion with *Astrothelium crassum*.

*Typus*: CHINA, Guangdong, Shenzhen City, Dapeng Peninsula National Geopark, alt. 167 m, on bark, 17 January 2019, F.Y. Liu GD19061 (LCUF, holotype).

*Diagnosis*: The new species differs from the similar species *Astrothelium crassum* (Fée) Aptroot and Lücking by the thin thallus and usually white-covered pseudostromata.

*Description*: Thallus crustose, corticolous, pale green, smooth to uneven, continuous, not surrounded by prothallus, 40–100 µm thick, covering areas up to 4 cm diam., not inducing gall formation of the host bark. Algae trentepohlioid. Ascomata perithecia, conical to pyriform, black, 0.3–0.6 mm diam., immersed to erumpent, sometimes single, but often 4–25 aggregated in pseudostromata. Pseudostromata surface white to grey, not concolorous to the thallus, raised, irregular to often linear in outline. Ostiole eccentric, fused, black, 0.2–0.5 mm diam., covered by a whitish rim. Ascomata wall carbonized, black, 50–160 μm thick. Hamathecium hyaline, clear, not interspersed. Asci cylindrical to clavate, 85–120 × 13–16 μm. Ascospores eight per ascus, biseriate to irregular, hyaline, fusiform, transversely three-septate, 20–27 × 6–9 μm, lumina diamond-shaped, surrounded by a smooth gelatinous sheath, 2–11 μm wide. Pycnidia not seen.

*Chemistry*: Thallus UV-, pseudostromata UV-. TLC: no substances detected.

*Habitat and distribution*: The new species grows on the bark of tropical and subtropical regions and is currently only found in China.

*Additional specimens examined*: CHINA. Guangdong, Shenzhen City, Dapeng Peninsula National Geopark, alt. 167 m, on bark, 17 January 2019, F.Y. Liu 20191093 (HMAS-L 0154762).

*Notes*: This species would key out in the recent world key [36] in key K at couplet 16 (Supplementary file S9) and differs from *A. crassum* by the thin thallus and usually white-covered pseudostromata. The latter was often characterized by ascomata with a whitish rim bordering a wide, dark ostiolar area and thickened thallus [12]. Further, they form independent branches in the phylogenetic tree constructed by LSU (Figure 1) and show large evolutionary divergence (8.2%; Supplementary file S7), which supports them as different species. Another related species *A*. *subdissocians* (Nyl. ex Vain.) Aptroot and Lücking can be distinguished by ascomata with complete whitish cover, including the ostiolar area [12]. *A. nitidiusculum* (Nyl.) Aptroot and Lücking is another similar species in having whitish ascomata with dark ostiolar area, but its pseudostromata are not distinct, and the ostioles are apical [12]. In addition, they form independent branches in the phylogenetic tree constructed by LSU (Figure 1) and show large evolutionary divergence (11.4%; Supplementary file S7), which supports them as two different species.

**Figure 6:**
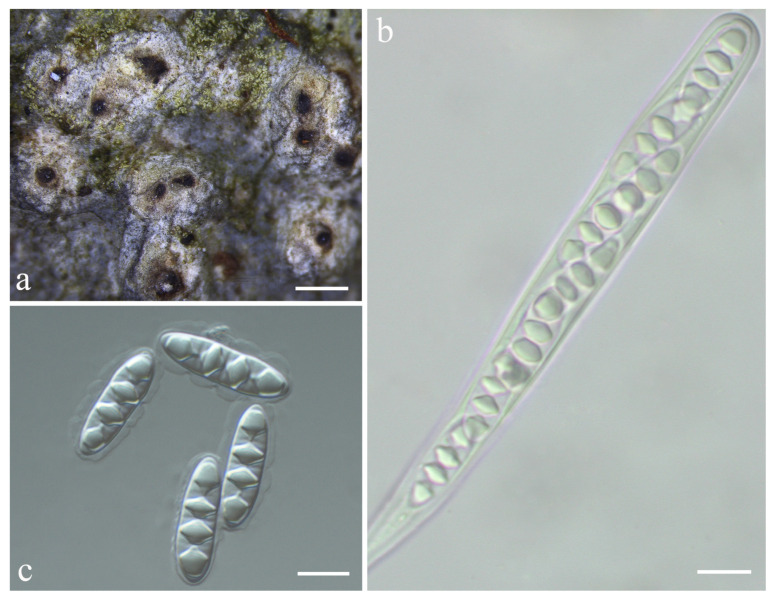
*Astrothelium subeustominspersum* sp. nov. (holotype, HMAS–L 0152001). (**a**) Thallus with ascomata; (**b**) ascus; (**c**) ascospores. Scale bars: (**a**) = 1 mm; (**b**,**c**) = 10 µm.

***Astrothelium subeustominspersum*** S.H. Jiang and C. Zhang, sp. nov. (Figure 6).

Fungal Names FN 571295.

*Etymology*: The epithet refers to the potential confusion with *Astrothelium eustominspersum*.

*Typus*: CHINA. Guangxi, Shangsi County, Shiwandashan National Nature Reserve, alt. 290 m, on bark, 21 March 2019, S.H. Jiang and C. Zhang 20190856 (HMAS–L 0152001, holotype).

*Diagnosis*: The new species can be distinguished from the most related species *Astrothelium eustominspersum* Aptroot and Oliveira-Junior by the lichexanthone only contained in the thallus.

*Description*: Thallus crustose, corticolous, greenish-grey to olive-green, smooth to uneven, somewhat shiny, continuous, covering areas up to 6 cm diam., 0.2–0.4 mm thick, not surrounded by a prothallus. Algae trentepohlioid. Ascomata perithecia, globose to pyriform, black, 0.15–0.4 mm diam., single to 2–15 aggregated groups immersed in pseudostromata. Pseudostromata surfaces white-grey to brownish, not concolorous to the thallus, raised, rounded to irregular. Ostiole eccentric, fused, black, 0.2–0.6 mm diam. Ascomata wall carbonized, 30–100 μm thick. Hamathecium hyaline, interspersed with oil droplets. Asci cylindrical to clavate, 88–170 × 13–17 μm. Ascospores eight per ascus, biseriate to irregular, hyaline, fusiform, transversely three-septate, 17–28 × 6–8 μm, lumina diamond-shaped, surrounded by a smooth gelatinous sheath, 1–6 μm wide. Pycnidia not seen.

Chemistry: Thallus UV+, pseudostromata UV-. TLC: lichexanthone.

*Habitat and distribution*: The new species grows on the bark of tropical and subtropical regions and is currently only found in China.

*Additional specimens examined*: CHINA. Guangxi, Shangsi County, Shiwandashan National Nature Reserve, alt. 290 m, on bark, 21 March 2019, S.H. Jiang and C. Zhang 20190855 (HMAS–L 0152000), 20190873 (HMAS–L 0152002).

*Notes*: This species would key out in the recent world key [36] in key J at couplet 17 (Supplementary file S9) and differs from the remaining species keying out there in that it has lichexanthone only in the thallus and that it has an interspersed hamathecium. The most similar species is *Astrothelium eustominspersum*, but the lichexanthone is only in the ostiole [39]. *A. studerae* Aptroot and M. Cáceres resembles this new species, but it can be distinguished by apical ostiole and lichexanthone only in ascomata [16]. *A. neovariolosum* Luangsuph., Aptroot and Sangvichien is also similar to this new species in pseudostromata and ascospores, but it differs in black prothallus, apical ostiole, and smaller perithecia and asci [29]. In addition, they formed two distinct clades in both ITS and ITS-LSU-RPB1 phylogenetic trees (Figure 2 and Supplementary file S6).

**Figure 7:**
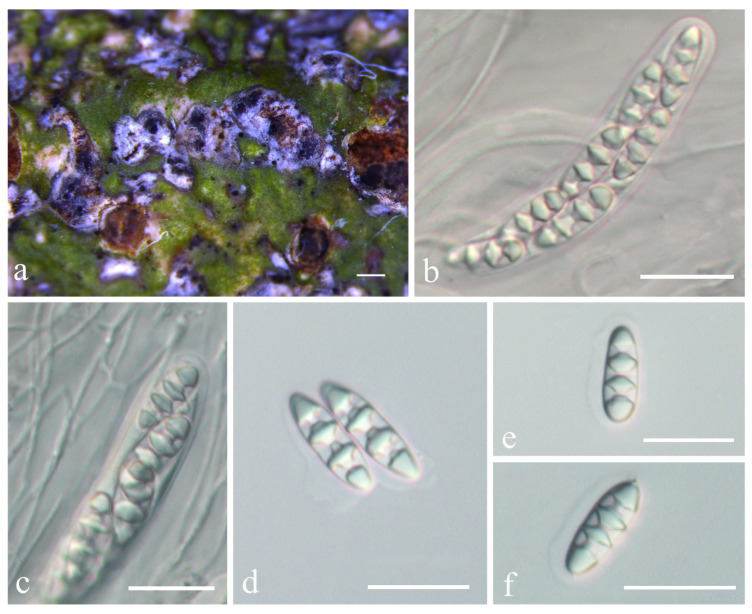
*Astrothelium subrufescens* sp. nov. (holotype, LCUF GD19067). (**a**) Thallus and pseudostromata; (**b**,**c**) ascus; (**d**–**f**) ascospores. Scale bars: (**a**) = 0.1 mm; (**b**–**f**) = 20 µm.

***Astrothelium subrufescens*** S.H. Jiang and C. Zhang, sp. nov. (Figure 7).

Fungal Names FN 571296.

*Etymology*: The epithet refers to the potential confusion with *Astrothelium rufescens*.

*Typus*: CHINA, Guangdong, Shenzhen City, Dapeng Peninsula National Geopark, alt. 167 m, on bark, 17 January 2019, F.Y. Liu GD19067 (LCUF, holotype).

*Diagnosis*: The new species can be distinguished from the somewhat similar species *Astrothelium rufescens* (Müll. Arg.) Aptroot and Lücking by the pseudostromata that contain few ascomata.

*Description*: Thallus crustose, corticolous, continuous, 1–9 cm diam., 30–90 µm thick, smooth to uneven, or bullate, olive-green. Algae trentepohlioid. Ascomata perithecia, black, conical to pyriform, 0.6–1.5 mm diam., immersed to erumpent in pseudostromata. Pseudostromata surfaces grey-white or grey-yellow, not concolorous to the thallus. Ostiole apical, black, with whitish surrounding area, 0.1–0.5 mm diam. Ascomata wall carbonized, black, 55–160 μm thick. Hamathecium is clear, not interspersed with oil droplets. Asci cylindrical to clavate, colorless, 85–117 × 13–16 µm. Ascospores eight per ascus, biseriate to irregular, hyaline, fusiform, ends rounded, transversely three-septate, 19–27 × 6–9 µm, lumina diamond-shaped, surrounded by a smooth gelatinous sheath, 1.5–11 μm wide. Pycnidia not seen.

*Chemistry*: Thallus UV-, pseudostromata UV-. TLC: no substance detected.

*Habitat and distribution*: The new species grows on the bark of tropical and subtropical regions and is currently only found in China.

*Additional specimens examined*: CHINA, Guangdong, Shenzhen City, Dapeng Peninsula National Geopark, alt. 167 m, on bark, 17 January 2019, F.Y. Liu GD19065 (LCUF), GD19086 (LCUF), GD19094 (LCUF). Hainan, Changjiang County, Bawangling National Nature Reserve, alt. 762 m, on bark, 8 December 2019, Z. Chao and Z.T. Yao HN20192176 (HMAS–L 0152007); Wuzhishan City, Wuzhishan National Nature Reserve, alt. 784 m, on bark, 12 December 2019, Z. Chao and Z.T. Yao HN20192312 (HMAS–L 0152041), HN20192317 (HMAS–L 0152042).

*Notes:* This species would key out in the recent world key [36] in key I at couplet 11–13 (Supplementary file S9). It is closely related to *Astrothelium rufescens*, but the latter species has pseudostromata containing more ascomata [12]. Further, from the LSU phylogenetic tree, they formed into two distinct clades (Figure 1), and their evolutionary divergence is far (13.6%; Supplementary file S7), which also supports them as different species. *A. crassum* also resembles this new species in whitish pseudostromata, but it differs in the ostiole lateral [12]. Furthermore, they formed independent lineages in the LSU tree (Figure 1), with a large genetic distance (7.3%; Supplementary file S7), indicating that they are two different species.

## 4. Discussion

The *Trypetheliaceae* is one of the oldest described families in the lichenized *Ascomycota* [2]. Based on molecular data, the core *Trypetheliaceae* were reorganized, with most species now in a single genus, *Astrothelium*, and additional lineages allocated in the other seventeen genera [1]. Species in *Astrothelium* often have a pantropical distribution [1,2,6,18,19]. In Southern China, there is abundant subtropical to tropical evergreen resources [40]. This habitat is favorable for the pyrenocarpous lichens, including *Astrothelium*. However, the genus has not been sufficiently studied; therefore, so far, four species have been recorded from China [17,23,24]. *A. speciosum* and *A. variolosum* were reported by Zahlbruckner (1933) for the first time from China [24]. After that, Aptroot and his colleagues contributed to the research on pyrenocarpous species and other microlichens in tropical China, especially in Hong Kong, Taiwan, and Xishuangbanna regions in Yunnan [23,41,42,43,44]. The vast majority of the species they encountered were new records for China. Furthermore, among those surveys of lichens in tropical China, *A. cinnamomeum* was reported in their annotated checklist of the lichens of Hong Kong [23]. During the investigation of lichenized fungi from (sub-)tropical China in our study, some samples were not able to be categorized as any previously described *Astrothelium* species. Based on morphological characteristics and phylogenetic analysis of the combined ITS, LSU, and RPB1 sequence datasets, there is sufficient evidence to verify five species new to science: *Astrothelium jiangxiense* sp. nov., *A. luminothallinum* sp. nov., *A. pseudocrassum* sp. nov., *A. subeustominspersum* sp. nov., and *A. subrufescens* sp. nov., even though the specimens were limited in this study.

In the modern circumscription [12], *Astrothelium* exhibits much variation in perithecial arrangement and ascospore septation, including septate and muriform ascospores. Five new species described here are all characterized by three-septate ascospores and surrounded by a smooth gelatinous sheath, indicating the diversity of *Astrothelium* is higher than previously understood. To better recognize the species relationship of *Astrothelium*, more taxonomic studies should be continuously carried out in the near future. More material will doubtlessly be found if the pyrenocarpous lichen flora in the area is investigated in more detail. Experience shows that with deeper studies, more rare species will be discovered, and there might be quite a number of yet undiscovered taxa. In addition, with further investigations, we expect to discover additional species within muriform ascospores in this genus from China.

## Data Availability

The newly generated sequences can be found in GenBank (https://www.ncbi.nlm.nih.gov/, accessed on 25 August 2022; Supplementary file S1). All new taxa were deposited in Fungal Names (https://nmdc.cn/fungalnames, accessed on 25 August 2022).

## Supplementary Materials

The following supporting information can be downloaded at: https://www.mdpi.com/article/10.3390/jof8100994/s1. Supplementary file S1: Specimens used in the phylogenetic analysis; Supplementary file S2: ITS_ alignment dataset; Supplementary file S3: Concatenated three-locus (ITS, LSU, RPB1) dataset; Supplementary file S4: The top hits obtained after running a BLAST search of ITS; Supplementary file S5: LSU_ alignment dataset; Supplementary file S6: The phylogenetic tree of ITS; Supplementary file S7: Estimates of evolutionary divergence between LSU sequences; Supplementary file S8: TLC test of the new speices *Astrothelium jiangxiense* sp. nov.; Supplementary file S9: World key to the species of *Pyrenulaceae* and *Trypetheliaceae*.
