# Supplementary material for: Morphological and Phylogenetic Characterizations Reveal Five New Species of Astrothelium (Trypetheliales, Ascomycota) from China
Source: J Fungi (Basel). 2022 Sep 22;8(10):994. doi: 10.3390/jof8100994 (PMC9604869; doi:10.3390/jof8100994)
Supplement: Supplementary file 1 [file jof-08-00994-s001.zip › jof-1907393-Supplementary file 1 Table.pdf]

Table S1. Specimens used in the phylogenetic analysis.

Species	Specimen No.	GenBank No. ITS	LSU	RPB1
Astrothelium aenascens	HRK93	LC127385	LC127403	LC128036
A. aenascens	HRK98	LC127386	LC127404	LC128037
A. aeneum	MPN302		KX215606	
A. cinereorosellum	MPN199C		KX215610	
A. cinnamomeum	DUKE	DQ782839	AY584652	DQ782824
A. confusum			GU327710	
A. crassum	MPN335		KM453761	
A. croceum	MPN55		KX215611	
A. degenerans	MPN266		KX215613	
A. degenerans	F16657		KX215612	
A. eustomum	MPN258		KM453762	
A. flavocoronatum	KY859	LC127381	LC127398	LC128030
A. flavocoronatum	TSL63	AB758900	LC127397	LC128031
A. floridanum	MPN216		KX215618	
A. floridanum	MPN214		KX215617	
A. galbineum	MPN260		KM453763	
A. grossum	MPN751		KX215623	
A. grossum	MPN657		KX215622	
A. grossum	MPN264		KX215621	
A. grossum	MPN263		KX215620	
A. grossum	MPN262		KX215619	
***A. jiangxiense* sp. nov.**	**LCUF JX19042**	**OP289105**	**OP289121**	**OP313732**
***A. jiangxiense* sp. nov.**	**LCUF JX19053**	**OP289106**	**OP289122**	**OP313733**
A. kunzei	MPN203B		KX215625	
A. kunzei	MPN201B		KX215624	
A. leucoconicum	MPN42		KM453764	
A. leucosessile	MPN58C		KX215627	
A. leucosessile	MPN265		KX215626	
***A. luminothallinum* sp. nov.**	**GZ18347**	**OP289110**	**OP289123**	--
***A. luminothallinum* sp. nov.**	**HMAS-L 0154761**	**OP289109**	--	--
A. macrocarpum	MPN57C		KX215631	
A. macrocarpum	MPN339		KX215630	
A. macrocarpum	MPN261		KX215629	
A. macrocarpum	MPN131		KX215628	
A. macrocarpum	NSR6	AB759880	LC127402	LC128033
A. macrocarpum	UBN37	LC127384	LC127400	LC128032
A. macrocarpum	UBN43	LC127383	LC127399	LC128034
A. macrocarpum	UBN113	LC127382	LC127401	LC128035
A. macrostiolatum	PHL84	LC127389	LC127407	LC128040
A. megaspermum	MPN234		KX215633	
A. megaspermum	MPN138		KX215632	
A. meristosporum	MPN198		KX215634	
A. neogalbineum	MPN51		KX215635	
A. neoinspersum	MPN61C		KX215636	
A. neglectum	TAK8	LC127392	LC127410	LC128043
A. neglectum	TAK12	LC127393	LC127411	LC128044
A. neglectum	TAK17	LC127394	LC127412	LC128045
A. neovariolosum	KY777	LC127390	LC127408	LC128041
A. neovariolosum	KY848	LC127391	LC127409	LC128042
A. nicaraguense	MPN213		KX215639	
A. nicaraguense	MPN211		KX215638	
A. nicaraguense	MPN205		KX215637	
A. nitidiusculum	MPN768		KX215640	
A. aff. *norisianum*	MPN23B		KX215607	
A. aff. *obscurum*	MPN194		KX215608	

A. obtectum	MPN422		KM453767	
A. phlyctaena	MPN386		KX215644	
A. phlyctaena	MPN381		KX215643	
A. phlyctaena	MPN376		KX215642	
A. phlyctaena	MPN373		KX215641	
***A. pseudocrassum* sp. nov.**	**LCUF GD19061**	**OP289108**	**OP289125**	**OP313735**
***A. pseudocrassum* sp. nov.**	**HMAS-L 0154762**	**OP289107**	**OP289124**	**OP313734**
A. robustum	MPN223		KX215649	
A. robustum	MPN222		KX215648	
A. robustum	MPN221		KX215647	
A. robustum	MPN212		KX215646	
A. robustum	MPN209		KX215645	
A. cf. *robustum*	MPN754		KM453760	
A. rufescens	MPN346		KX215652	
A. rufescens	MPN345		KX215651	
A. rufescens	MPN143		KX215650	
A. siamense	KRB105	LC127387	LC127405	LC128038
A. siamense	KRB139	LC127388	LC127406	LC128039
A. sanguinarium	MPN767		KX215654	
A. sanguinarium	MPN766		KX215653	
A. aff. *scorioides*	MPN703		KM453765	
A. scoria	MPN310		KX215655	
A. scorioides	MPN770		KM453766	
A. aff. *sepultum*	MPN229		KX215609	
A. sp.	MPN96		KX215614	
A. sp.	MPN390		KX215658	
A. sp.	MPN162		KX215657	
A. sp.	MPN133		KX215656	
A. subendochryseum	MPN202B		KX215659	
A. subinterjectum	MPN157		KX215660	
***A. subeustominspersum* sp. nov.**	**HMAS-L 0152000**	**OP289118**	**OP289126**	**OP313736**
***A. subeustominspersum* sp. nov.**	**HMAS-L 0152001**	**OP289119**	**OP289127**	**OP313737**
***A. subeustominspersum* sp. nov.**	**HMAS-L 0152002**	**OP289120**	**OP289128**	**OP313738**
***A. subrufescens* sp. nov.**	**GD19065**	**OP289111**	--	--
***A. subrufescens* sp. nov.**	**GD19067**	**OP289112**	**OP289129**	--
***A. subrufescens* sp. nov.**	**GD19086**	**OP289113**	**OP289130**	--
***A. subrufescens* sp. nov.**	**GD19094**	**OP289114**	--	--
***A. subrufescens* sp. nov.**	**HMAS-L 0152007**	**OP289115**	--	--
***A. subrufescens* sp. nov.**	**HMAS-L 0152041**	**OP289116**	--	--
***A. subrufescens* sp. nov.**	**HMAS-L 0152042**	**OP289117**	--	--
A. subscoria	MPN325		KX215661	
A. variolosum	MPN41		KX215662	
A. variolosum	MPN43		KM453768	
A. versicolor	MPN259		KM453769	
Bathelium madreporiforme	NAN95	LC127396	LC127414	LC128047
B. madreporiforme	UBN147	LC127395	LC127413	LC128046

[1] The new sequences generated in this study were in bold.
