# Supplementary material for: Morphological and Phylogenetic Characterizations Reveal Five New Species of Astrothelium (Trypetheliales, Ascomycota) from China
Source: J Fungi (Basel). 2022 Sep 22;8(10):994. doi: 10.3390/jof8100994 (PMC9604869; doi:10.3390/jof8100994)
Supplement: Supplementary file 1 [file jof-08-00994-s001.zip › jof-1907393-Supplementary file 4 The top hits obtained after running a BLAST search of ITS.pdf]

1. The top hits obtained after running a BLAST search of ITS for *Astrothelium jiangxiense* sp. nov. (holotype JX19042)

Descriptions | Graphic Summary | Alignments | Taxonomy

Sequences producing significant alignments Download Select columns Show 100

select all *100 sequences selected* GenBank Graphics Distance tree of results MSA Viewer

Description	Scientific Name	Max Score	Total Score	Query Cover	E value	Per. Ident	Acc. Len	Accession
Astrothelium neovariolosum genes for 18S rRNA, ITS1, 5.8S rRNA, ITS2, 28S rRNA, partial and complete seque...	Astrothelium neo...	480	480	95%	2e-130	85.33%	545	LC127391.1
Astrothelium neovariolosum genes for 18S rRNA, ITS1, 5.8S rRNA, ITS2, 28S rRNA, partial and complete seque...	Astrothelium neo...	480	480	95%	2e-130	85.33%	544	LC127390.1
Astrothelium macrostiolatum genes for 18S rRNA, ITS1, 5.8S rRNA, ITS2, 28S rRNA, partial and complete seque...	Astrothelium mac...	477	477	97%	7e-130	84.90%	605	LC127389.1
Astrothelium siamense genes for 18S rRNA, ITS1, 5.8S rRNA, ITS2, 28S rRNA, partial and complete sequence, i...	Astrothelium sia...	452	452	90%	3e-122	85.20%	592	LC127388.1
Astrothelium flavocoronatum genes for 18S rRNA, ITS1, 5.8S rRNA, ITS2, 28S rRNA, partial and complete seque...	Astrothelium flav...	451	451	90%	1e-121	85.65%	543	AB758900.1
Astrothelium flavocoronatum genes for 18S rRNA, ITS1, 5.8S rRNA, ITS2, 28S rRNA, partial and complete seque...	Astrothelium flav...	444	444	90%	4e-120	85.45%	545	LC127381.1
Astrothelium aenascens genes for 18S rRNA, ITS1, 5.8S rRNA, ITS2, 28S rRNA, partial and complete sequence,...	Astrothelium aen...	442	442	89%	5e-119	84.41%	594	LC127385.1
Astrothelium siamense genes for 18S rRNA, ITS1, 5.8S rRNA, ITS2, 28S rRNA, partial and complete sequence, i...	Astrothelium sia...	439	439	89%	2e-118	84.86%	583	LC127387.1
Astrothelium aenascens genes for 18S rRNA, ITS1, 5.8S rRNA, ITS2, partial and complete sequence, isolate: HR...	Astrothelium aen...	433	433	87%	3e-116	84.52%	550	LC127386.1
Trypethelium sp. DUKE 0000007 isolate AFTOL-ID 110 internal transcribed spacer 1, 5.8S ribosomal RNA gene,...	Astrothelium cinn...	427	427	88%	1e-114	83.41%	483	DQ782839.1
Astrothelium neglectum genes for 18S rRNA, ITS1, 5.8S rRNA, ITS2, 28S rRNA, partial and complete sequence, i...	Astrothelium negl...	419	419	90%	2e-112	82.49%	552	LC127394.1
Astrothelium neglectum genes for 18S rRNA, ITS1, 5.8S rRNA, ITS2, 28S rRNA, partial and complete sequence, i...	Astrothelium negl...	419	419	90%	2e-112	82.49%	568	LC127393.1
Astrothelium neglectum genes for 18S rRNA, ITS1, 5.8S rRNA, ITS2, 28S rRNA, partial and complete sequence, i...	Astrothelium negl...	419	419	90%	2e-112	82.49%	567	LC127392.1
Astrothelium sp. ZTY-2020a voucher LCUF GD19282 small subunit ribosomal RNA gene, partial sequence; intern...	Astrothelium sp....	408	408	90%	3e-109	83.29%	603	MT948055.1
Astrothelium macrocarpum genes for 18S rRNA, ITS1, 5.8S rRNA, ITS2, 28S rRNA, partial and complete sequen...	Astrothelium mac...	407	484	88%	1e-108	86.57%	784	LC127384.1
Astrothelium macrocarpum genes for 18S rRNA, ITS1, 5.8S rRNA, ITS2, 28S rRNA, partial and complete sequen...	Astrothelium mac...	407	484	88%	1e-108	86.57%	800	LC127383.1
Astrothelium macrocarpum genes for 18S rRNA, ITS1, 5.8S rRNA, ITS2, 28S rRNA, partial and complete sequen...	Astrothelium mac...	407	484	88%	1e-108	86.57%	800	LC127382.1
Astrothelium sp. ZTY-2020a voucher LCUF GD19156 small subunit ribosomal RNA gene, partial sequence; intern...	Astrothelium sp....	404	404	90%	1e-107	83.06%	602	MT948056.1
Astrothelium macrocarpum TNS:YO12392 genes for 18S rRNA, ITS1, 5.8S rRNA, ITS2, 28S rRNA, partial and co...	Astrothelium mac...	403	475	94%	1e-107	84.43%	810	LC573991.1
Astrothelium macrocarpum genes for 18S rRNA, ITS1, 5.8S rRNA, ITS2, 28S rRNA, partial and complete sequence	Astrothelium mac...	402	475	88%	4e-107	86.29%	799	AB759880.1
Astrothelium macrocarpum voucher HMAS-L0146701 small subunit ribosomal RNA gene, partial sequence; intern...	Astrothelium mac...	402	475	88%	4e-107	86.29%	800	MT948054.1
Astrothelium macrocarpum TNS:YO12285 genes for 18S rRNA, ITS1, 5.8S rRNA, ITS2, 28S rRNA, partial and co...	Astrothelium mac...	398	475	89%	5e-106	85.84%	726	LC573990.1
Astrothelium sp. isolate JAM-177 small subunit ribosomal RNA gene, partial sequence; internal transcribed space...	Astrothelium sp.	393	393	89%	2e-104	82.35%	621	MT553327.1
Trypethelium sp. isolate JAM-176 small subunit ribosomal RNA gene, partial sequence; internal transcribed space...	Trypethelium sp.	334	334	71%	5e-87	83.78%	622	MT553326.1
Uncultured Capnodiales clone Hj93 18S ribosomal RNA gene, partial sequence; internal transcribed spacer 1, 5.8...	uncultured Capn...	326	326	68%	3e-84	84.28%	617	JX317516.1
Marcelaria cumingii genes for 18S rRNA, ITS1, 5.8S rRNA, ITS2, 28S rRNA, partial and complete sequence, isola...	Marcelaria cumingii	286	286	49%	2e-72	88.03%	556	LC034180.1

2. The top hits obtained after running a BLAST search of ITS for *Astrothelium luminothallinum* sp. nov. (holotype GZ18347)

Descriptions | Graphic Summary | Alignments | Taxonomy

Sequences producing significant alignments Download Select columns Show 100

select all *100 sequences selected* GenBank Graphics Distance tree of results MSA Viewer

Description	Scientific Name	Max Score	Total Score	Query Cover	E value	Per. Ident	Acc. Len	Accession
Astrothelium macrostiolatum genes for 18S rRNA, ITS1, 5.8S rRNA, ITS2, 28S rRNA, partial and complete seque...	Astrothelium mac...	551	551	100%	4e-152	87.28%	605	LC127389.1
Astrothelium neovariolosum genes for 18S rRNA, ITS1, 5.8S rRNA, ITS2, 28S rRNA, partial and complete seque...	Astrothelium neo...	523	523	92%	2e-143	88.10%	545	LC127391.1
Astrothelium neovariolosum genes for 18S rRNA, ITS1, 5.8S rRNA, ITS2, 28S rRNA, partial and complete seque...	Astrothelium neo...	523	523	92%	2e-143	88.10%	544	LC127390.1
Astrothelium siamense genes for 18S rRNA, ITS1, 5.8S rRNA, ITS2, 28S rRNA, partial and complete sequence, i...	Astrothelium sia...	517	517	98%	8e-142	86.50%	592	LC127388.1
Astrothelium siamense genes for 18S rRNA, ITS1, 5.8S rRNA, ITS2, 28S rRNA, partial and complete sequence, i...	Astrothelium sia...	512	512	100%	3e-140	86.09%	583	LC127387.1
Astrothelium aenascens genes for 18S rRNA, ITS1, 5.8S rRNA, ITS2, 28S rRNA, partial and complete sequence,...	Astrothelium aen...	509	509	92%	1e-139	87.71%	594	LC127385.1
Astrothelium aenascens genes for 18S rRNA, ITS1, 5.8S rRNA, ITS2, partial and complete sequence, isolate: HR...	Astrothelium aen...	500	500	90%	6e-137	87.89%	550	LC127386.1
Astrothelium sp. ZTY-2020a voucher LCUF GD19282 small subunit ribosomal RNA gene, partial sequence; intern...	Astrothelium sp....	452	452	92%	3e-122	85.31%	603	MT948055.1
Astrothelium sp. ZTY-2020a voucher LCUF GD19156 small subunit ribosomal RNA gene, partial sequence; intern...	Astrothelium sp....	447	447	92%	1e-120	85.08%	602	MT948056.1
Astrothelium flavocoronatum genes for 18S rRNA, ITS1, 5.8S rRNA, ITS2, 28S rRNA, partial and complete seque...	Astrothelium flav...	425	425	90%	4e-114	83.69%	545	LC127381.1
Astrothelium flavocoronatum genes for 18S rRNA, ITS1, 5.8S rRNA, ITS2, 28S rRNA, partial and complete seque...	Astrothelium flav...	422	422	90%	5e-113	83.65%	543	AB758900.1
Astrothelium neglectum genes for 18S rRNA, ITS1, 5.8S rRNA, ITS2, 28S rRNA, partial and complete sequence, i...	Astrothelium negl...	407	407	92%	1e-108	81.94%	552	LC127394.1
Astrothelium neglectum genes for 18S rRNA, ITS1, 5.8S rRNA, ITS2, 28S rRNA, partial and complete sequence, i...	Astrothelium negl...	407	407	92%	1e-108	81.94%	568	LC127393.1
Astrothelium neglectum genes for 18S rRNA, ITS1, 5.8S rRNA, ITS2, 28S rRNA, partial and complete sequence, i...	Astrothelium negl...	407	407	92%	1e-108	81.94%	567	LC127392.1
Trypethelium sp. DUKE 0000007 isolate AFTOL-ID 110 internal transcribed spacer 1, 5.8S ribosomal RNA gene,...	Astrothelium cinn...	402	402	89%	4e-107	82.82%	483	DQ782839.1
Astrothelium sp. isolate JAM-177 small subunit ribosomal RNA gene, partial sequence; internal transcribed space...	Astrothelium sp.	373	373	90%	2e-98	81.03%	621	MT553327.1
Astrothelium macrocarpum genes for 18S rRNA, ITS1, 5.8S rRNA, ITS2, 28S rRNA, partial and complete sequen...	Astrothelium mac...	369	428	73%	3e-97	90.41%	784	LC127384.1
Astrothelium macrocarpum genes for 18S rRNA, ITS1, 5.8S rRNA, ITS2, 28S rRNA, partial and complete sequen...	Astrothelium mac...	369	428	73%	3e-97	90.41%	800	LC127383.1
Astrothelium macrocarpum genes for 18S rRNA, ITS1, 5.8S rRNA, ITS2, 28S rRNA, partial and complete sequen...	Astrothelium mac...	369	428	73%	3e-97	90.41%	800	LC127382.1
Astrothelium macrocarpum TNS:YO12392 genes for 18S rRNA, ITS1, 5.8S rRNA, ITS2, 28S rRNA, partial and co...	Astrothelium mac...	369	432	73%	3e-97	90.41%	810	LC573991.1
Astrothelium macrocarpum genes for 18S rRNA, ITS1, 5.8S rRNA, ITS2, 28S rRNA, partial and complete sequence	Astrothelium mac...	364	419	73%	1e-95	90.04%	799	AB759880.1
Astrothelium macrocarpum voucher HMAS-L0146701 small subunit ribosomal RNA gene, partial sequence; intern...	Astrothelium mac...	364	419	73%	1e-95	90.04%	800	MT948054.1
Astrothelium macrocarpum TNS:YO12285 genes for 18S rRNA, ITS1, 5.8S rRNA, ITS2, 28S rRNA, partial and co...	Astrothelium mac...	361	420	78%	1e-94	87.41%	726	LC573990.1
Trypethelium sp. isolate JAM-176 small subunit ribosomal RNA gene, partial sequence; internal transcribed space...	Trypethelium sp.	356	356	72%	2e-93	85.12%	622	MT553326.1
Uncultured Capnodiales clone Hj93 18S ribosomal RNA gene, partial sequence; internal transcribed spacer 1, 5.8...	uncultured Capn...	332	332	67%	7e-86	86.04%	617	JX317516.1
Marcelaria cumingii genes for 18S rRNA, ITS1, 5.8S rRNA, ITS2, 28S rRNA, partial and complete sequence, isola...	Marcelaria cumingii	285	285	37%	9e-72	97.09%	556	LC034180.1
Marcelaria cumingii genes for 18S rRNA, ITS1, 5.8S rRNA, ITS2, 28S rRNA, partial and complete sequence, isola...	Marcelaria cumingii	285	285	37%	9e-72	97.09%	555	LC034179.1

3. The top hits obtained after running a BLAST search of ITS for *Astrothelium pseudocrassum* sp. nov. (holotype GD19061)

Descriptions | Graphic Summary | Alignments | Taxonomy

Sequences producing significant alignments — Download — Select columns — Show 100

select all *100 sequences selected* — GenBank — Graphics — Distance tree of results — MSA Viewer

Description	Scientific Name	Max Score	Total Score	Query Cover	E value	Per. Ident	Acc. Len	Accession
Astrothelium macrostiolatum genes for 18S rRNA, ITS1, 5.8S rRNA, ITS2, 28S rRNA, partial and complete seque...	Astrothelium mac...	579	579	100%	3e-160	88.98%	605	LC127389.1
Astrothelium neovariolosum genes for 18S rRNA, ITS1, 5.8S rRNA, ITS2, 28S rRNA, partial and complete sequen...	Astrothelium neo...	543	543	96%	2e-149	88.01%	545	LC127391.1
Astrothelium neovariolosum genes for 18S rRNA, ITS1, 5.8S rRNA, ITS2, 28S rRNA, partial and complete sequen...	Astrothelium neo...	543	543	96%	2e-149	88.01%	544	LC127390.1
Astrothelium siamense genes for 18S rRNA, ITS1, 5.8S rRNA, ITS2, 28S rRNA, partial and complete sequence, is...	Astrothelium sia...	527	527	97%	4e-145	87.22%	592	LC127388.1
Astrothelium aenascens genes for 18S rRNA, ITS1, 5.8S rRNA, ITS2, 28S rRNA, partial and complete sequence, i...	Astrothelium aen...	526	526	96%	2e-144	87.84%	594	LC127385.1
Astrothelium siamense genes for 18S rRNA, ITS1, 5.8S rRNA, ITS2, 28S rRNA, partial and complete sequence, is...	Astrothelium sia...	517	517	97%	8e-142	86.80%	583	LC127387.1
Astrothelium aenascens genes for 18S rRNA, ITS1, 5.8S rRNA, ITS2, partial and complete sequence, isolate: HR...	Astrothelium aen...	517	517	93%	8e-142	88.02%	550	LC127386.1
Astrothelium flavocoronatum genes for 18S rRNA, ITS1, 5.8S rRNA, ITS2, 28S rRNA, partial and complete seque...	Astrothelium flav...	425	425	89%	4e-114	83.69%	543	AB758900.1
Astrothelium flavocoronatum genes for 18S rRNA, ITS1, 5.8S rRNA, ITS2, 28S rRNA, partial and complete seque...	Astrothelium flav...	423	423	89%	1e-113	83.49%	545	LC127381.1
Astrothelium sp. ZTY-2020a voucher LCUF GD19282 small subunit ribosomal RNA gene, partial sequence; intern...	Astrothelium sp. ...	392	392	91%	2e-104	82.13%	603	MT948055.1
Astrothelium sp. isolate JAM-177 small subunit ribosomal RNA gene, partial sequence; internal transcribed spacer...	Astrothelium sp.	390	390	89%	8e-104	82.27%	621	MT553327.1
Astrothelium sp. ZTY-2020a voucher LCUF GD19156 small subunit ribosomal RNA gene, partial sequence; intern...	Astrothelium sp. ...	389	389	91%	3e-103	81.86%	602	MT948056.1
Trypethelium sp. isolate JAM-176 small subunit ribosomal RNA gene, partial sequence; internal transcribed spacer...	Trypethelium sp.	379	379	72%	1e-100	87.13%	622	MT553326.1
Astrothelium neglectum genes for 18S rRNA, ITS1, 5.8S rRNA, ITS2, 28S rRNA, partial and complete sequence, i...	Astrothelium negl...	379	379	92%	1e-100	81.35%	552	LC127394.1
Astrothelium neglectum genes for 18S rRNA, ITS1, 5.8S rRNA, ITS2, 28S rRNA, partial and complete sequence, i...	Astrothelium negl...	379	379	92%	1e-100	81.35%	568	LC127393.1
Astrothelium neglectum genes for 18S rRNA, ITS1, 5.8S rRNA, ITS2, 28S rRNA, partial and complete sequence, i...	Astrothelium negl...	379	379	92%	1e-100	81.35%	567	LC127392.1
Astrothelium macrocarpum genes for 18S rRNA, ITS1, 5.8S rRNA, ITS2, 28S rRNA, partial and complete sequenc...	Astrothelium mac...	362	435	85%	4e-95	85.45%	784	LC127384.1
Astrothelium macrocarpum genes for 18S rRNA, ITS1, 5.8S rRNA, ITS2, 28S rRNA, partial and complete sequenc...	Astrothelium mac...	362	435	85%	4e-95	85.45%	800	LC127383.1
Astrothelium macrocarpum genes for 18S rRNA, ITS1, 5.8S rRNA, ITS2, 28S rRNA, partial and complete sequenc...	Astrothelium mac...	362	435	85%	4e-95	85.45%	800	LC127382.1
Astrothelium macrocarpum genes for 18S rRNA, ITS1, 5.8S rRNA, ITS2, 28S rRNA, partial and complete sequence	Astrothelium mac...	358	426	85%	5e-94	85.15%	799	AB759880.1
Astrothelium macrocarpum voucher HMAS-L0146701 small subunit ribosomal RNA gene, partial sequence; intern...	Astrothelium mac...	358	426	85%	5e-94	85.15%	800	MT948054.1
Trypethelium sp. DUKE 0000007 isolate AFTOL-ID 110 internal transcribed spacer 1, 5.8S ribosomal RNA gene, a...	Astrothelium cinn...	356	356	87%	2e-93	80.85%	483	DQ782839.1
Astrothelium macrocarpum TNS:YO12285 genes for 18S rRNA, ITS1, 5.8S rRNA, ITS2, 28S rRNA, partial and co...	Astrothelium mac...	355	428	85%	6e-93	84.98%	726	LC573990.1
Astrothelium macrocarpum TNS:YO12392 genes for 18S rRNA, ITS1, 5.8S rRNA, ITS2, 28S rRNA, partial and co...	Astrothelium mac...	352	420	85%	7e-92	84.80%	810	LC573991.1
Uncultured Capnodiales clone Hj93 18S ribosomal RNA gene, partial sequence; internal transcribed spacer 1, 5.8...	uncultured Capn...	345	345	66%	3e-90	86.60%	617	JX317516.1
Marcelaria cumingii genes for 18S rRNA, ITS1, 5.8S rRNA, ITS2, 28S rRNA, partial and complete sequence, isolat...	Marcelaria cumingii	307	307	67%	8e-79	82.81%	555	LC034179.1
Marcelaria cumingii genes for 18S rRNA, ITS1, 5.8S rRNA, ITS2, 28S rRNA, partial and complete sequence, isolat...	Marcelaria cumingii	303	303	67%	3e-77	82.50%	556	LC034180.1

4. The top hits obtained after running a BLAST search of ITS for *Astrothelium subeustominspersum* sp. nov. (holotype HMAS–L 0152001)

Descriptions | Graphic Summary | Alignments | Taxonomy

Sequences producing significant alignments — Download — Select columns — Show 100

select all *100 sequences selected* — GenBank — Graphics — Distance tree of results — MSA Viewer

Description	Scientific Name	Max Score	Total Score	Query Cover	E value	Per. Ident	Acc. Len	Accession
Astrothelium macrostiolatum genes for 18S rRNA, ITS1, 5.8S rRNA, ITS2, 28S rRNA, partial and complete seque...	Astrothelium mac...	508	508	95%	4e-139	85.78%	605	LC127389.1
Astrothelium neovariolosum genes for 18S rRNA, ITS1, 5.8S rRNA, ITS2, 28S rRNA, partial and complete seque...	Astrothelium neo...	490	490	95%	1e-133	85.49%	545	LC127391.1
Astrothelium neovariolosum genes for 18S rRNA, ITS1, 5.8S rRNA, ITS2, 28S rRNA, partial and complete seque...	Astrothelium neo...	490	490	95%	1e-133	85.49%	544	LC127390.1
Astrothelium aenascens genes for 18S rRNA, ITS1, 5.8S rRNA, ITS2, 28S rRNA, partial and complete sequence, ...	Astrothelium aen...	468	468	91%	4e-127	85.58%	594	LC127385.1
Astrothelium aenascens genes for 18S rRNA, ITS1, 5.8S rRNA, ITS2, partial and complete sequence, isolate: HR...	Astrothelium aen...	459	459	91%	2e-124	85.11%	550	LC127386.1
Astrothelium siamense genes for 18S rRNA, ITS1, 5.8S rRNA, ITS2, 28S rRNA, partial and complete sequence, i...	Astrothelium sia...	449	449	92%	3e-121	84.78%	583	LC127387.1
Astrothelium siamense genes for 18S rRNA, ITS1, 5.8S rRNA, ITS2, 28S rRNA, partial and complete sequence, i...	Astrothelium sia...	433	433	92%	3e-116	83.29%	592	LC127388.1
Astrothelium flavocoronatum genes for 18S rRNA, ITS1, 5.8S rRNA, ITS2, 28S rRNA, partial and complete seque...	Astrothelium flav...	431	431	89%	9e-116	83.81%	543	AB758900.1
Astrothelium flavocoronatum genes for 18S rRNA, ITS1, 5.8S rRNA, ITS2, 28S rRNA, partial and complete seque...	Astrothelium flav...	425	425	89%	4e-114	83.61%	545	LC127381.1
Astrothelium sp. isolate JAM-177 small subunit ribosomal RNA gene, partial sequence; internal transcribed space...	Astrothelium sp.	393	393	88%	2e-104	82.46%	621	MT553327.1
Astrothelium macrocarpum genes for 18S rRNA, ITS1, 5.8S rRNA, ITS2, 28S rRNA, partial and complete sequen...	Astrothelium mac...	379	436	90%	1e-100	84.17%	784	LC127384.1
Astrothelium macrocarpum genes for 18S rRNA, ITS1, 5.8S rRNA, ITS2, 28S rRNA, partial and complete sequen...	Astrothelium mac...	379	436	90%	1e-100	84.17%	800	LC127383.1
Astrothelium macrocarpum genes for 18S rRNA, ITS1, 5.8S rRNA, ITS2, 28S rRNA, partial and complete sequen...	Astrothelium mac...	379	436	90%	1e-100	84.17%	800	LC127382.1
Astrothelium macrocarpum TNS:YO12392 genes for 18S rRNA, ITS1, 5.8S rRNA, ITS2, 28S rRNA, partial and co...	Astrothelium mac...	377	438	84%	2e-99	86.20%	810	LC573991.1
Astrothelium macrocarpum genes for 18S rRNA, ITS1, 5.8S rRNA, ITS2, 28S rRNA, partial and complete sequence	Astrothelium mac...	375	375	77%	6e-99	83.89%	799	AB759880.1
Astrothelium macrocarpum voucher HMAS-L0146701 small subunit ribosomal RNA gene, partial sequence; intern...	Astrothelium mac...	375	375	77%	6e-99	83.89%	800	MT948054.1
Astrothelium sp. ZTY-2020a voucher LCUF GD19282 small subunit ribosomal RNA gene, partial sequence; intern...	Astrothelium sp. ...	371	371	91%	7e-98	81.82%	603	MT948055.1
Astrothelium macrocarpum TNS:YO12285 genes for 18S rRNA, ITS1, 5.8S rRNA, ITS2, 28S rRNA, partial and co...	Astrothelium mac...	369	425	84%	3e-97	85.37%	726	LC573990.1
Astrothelium sp. ZTY-2020a voucher LCUF GD19156 small subunit ribosomal RNA gene, partial sequence; intern...	Astrothelium sp. ...	367	367	91%	9e-97	81.59%	602	MT948056.1
Trypethelium sp. isolate JAM-176 small subunit ribosomal RNA gene, partial sequence; internal transcribed space...	Trypethelium sp.	352	352	71%	7e-92	84.64%	622	MT553326.1
Astrothelium neglectum genes for 18S rRNA, ITS1, 5.8S rRNA, ITS2, 28S rRNA, partial and complete sequence, i...	Astrothelium negl...	347	347	91%	9e-91	78.33%	552	LC127394.1
Astrothelium neglectum genes for 18S rRNA, ITS1, 5.8S rRNA, ITS2, 28S rRNA, partial and complete sequence, i...	Astrothelium negl...	347	347	91%	9e-91	78.33%	568	LC127393.1
Astrothelium neglectum genes for 18S rRNA, ITS1, 5.8S rRNA, ITS2, 28S rRNA, partial and complete sequence, i...	Astrothelium negl...	347	347	91%	9e-91	78.33%	567	LC127392.1
Uncultured Capnodiales clone Hj93 18S ribosomal RNA gene, partial sequence; internal transcribed spacer 1, 5.8...	uncultured Capn...	334	334	66%	2e-86	85.48%	617	JX317516.1
Trypethelium sp. DUKE 0000007 isolate AFTOL-ID 110 internal transcribed spacer 1, 5.8S ribosomal RNA gene, ...	Astrothelium cinn...	330	330	87%	2e-85	78.44%	483	DQ782839.1
Uncultured fungus clone 107A66967 18S ribosomal RNA gene, partial sequence; internal transcribed spacer 1, c...	uncultured fungus	286	286	40%	2e-72	94.54%	383	JX333079.1
Marcelaria cumingii genes for 18S rRNA, ITS1, 5.8S rRNA, ITS2, 28S rRNA, partial and complete sequence, isola...	Marcelaria cumingii	284	284	43%	9e-72	91.67%	555	LC034179.1

5. The top hits obtained after running a BLAST search of ITS for *Astrothelium subrufescens* sp. nov. (holotype GD19067)

Descriptions | Graphic Summary | Alignments | Taxonomy

Sequences producing significant alignments　　**Download**　**Select columns**　Show 100

select all *100 sequences selected*　　GenBank　Graphics　Distance tree of results　MSA Viewer

Description	Scientific Name	Max Score	Total Score	Query Cover	E value	Per. Ident	Acc. Len	Accession
Astrothelium macrostiolatum genes for 18S rRNA, ITS1, 5.8S rRNA, ITS2, 28S rRNA, partial and complete sequen...	Astrothelium mac...	605	605	100%	2e-168	90.06%	605	LC127389.1
Astrothelium neovariolosum genes for 18S rRNA, ITS1, 5.8S rRNA, ITS2, 28S rRNA, partial and complete sequenc...	Astrothelium neo...	578	578	100%	3e-160	88.72%	545	LC127391.1
Astrothelium neovariolosum genes for 18S rRNA, ITS1, 5.8S rRNA, ITS2, 28S rRNA, partial and complete sequenc...	Astrothelium neo...	578	578	100%	3e-160	88.72%	544	LC127390.1
Astrothelium siamense genes for 18S rRNA, ITS1, 5.8S rRNA, ITS2, 28S rRNA, partial and complete sequence, iso...	Astrothelium sia...	535	535	96%	3e-147	87.64%	592	LC127388.1
Astrothelium aenascens genes for 18S rRNA, ITS1, 5.8S rRNA, ITS2, 28S rRNA, partial and complete sequence, is...	Astrothelium aen...	519	519	100%	2e-142	86.39%	594	LC127385.1
Astrothelium siamense genes for 18S rRNA, ITS1, 5.8S rRNA, ITS2, 28S rRNA, partial and complete sequence, iso...	Astrothelium sia...	509	509	95%	1e-139	86.82%	583	LC127387.1
Astrothelium aenascens genes for 18S rRNA, ITS1, 5.8S rRNA, ITS2, partial and complete sequence, isolate: HRK98	Astrothelium aen...	509	509	93%	1e-139	87.53%	550	LC127386.1
Astrothelium flavocoronatum genes for 18S rRNA, ITS1, 5.8S rRNA, ITS2, 28S rRNA, partial and complete sequence	Astrothelium flav...	435	435	89%	2e-117	84.09%	543	AB758900.1
Astrothelium flavocoronatum genes for 18S rRNA, ITS1, 5.8S rRNA, ITS2, 28S rRNA, partial and complete sequen...	Astrothelium flav...	434	434	89%	7e-117	83.89%	545	LC127381.1
Astrothelium neglectum genes for 18S rRNA, ITS1, 5.8S rRNA, ITS2, 28S rRNA, partial and complete sequence, is...	Astrothelium negl...	400	400	94%	2e-106	81.54%	552	LC127394.1
Astrothelium neglectum genes for 18S rRNA, ITS1, 5.8S rRNA, ITS2, 28S rRNA, partial and complete sequence, is...	Astrothelium negl...	400	400	94%	2e-106	81.54%	568	LC127393.1
Astrothelium neglectum genes for 18S rRNA, ITS1, 5.8S rRNA, ITS2, 28S rRNA, partial and complete sequence, is...	Astrothelium negl...	400	400	94%	2e-106	81.54%	567	LC127392.1
Astrothelium sp. ZTY-2020a voucher LCUF GD19156 small subunit ribosomal RNA gene, partial sequence; internal...	Astrothelium sp. ...	398	398	89%	5e-106	82.51%	602	MT948056.1
Astrothelium sp. ZTY-2020a voucher LCUF GD19282 small subunit ribosomal RNA gene, partial sequence; internal...	Astrothelium sp. ...	398	398	89%	5e-106	82.55%	603	MT948055.1
Trypethelium sp. DUKE 0000007 isolate AFTOL-ID 110 internal transcribed spacer 1, 5.8S ribosomal RNA gene, a...	Astrothelium cinn...	396	396	92%	2e-105	81.53%	483	DQ782839.1
Astrothelium sp. isolate JAM-177 small subunit ribosomal RNA gene, partial sequence; internal transcribed spacer ...	Astrothelium sp.	390	390	89%	8e-104	82.27%	621	MT553327.1
Trypethelium sp. isolate JAM-176 small subunit ribosomal RNA gene, partial sequence; internal transcribed spacer ...	Trypethelium sp.	386	386	71%	3e-102	87.35%	622	MT553326.1
Astrothelium macrocarpum genes for 18S rRNA, ITS1, 5.8S rRNA, ITS2, 28S rRNA, partial and complete sequence...	Astrothelium mac...	360	433	92%	1e-94	83.61%	784	LC127384.1
Astrothelium macrocarpum genes for 18S rRNA, ITS1, 5.8S rRNA, ITS2, 28S rRNA, partial and complete sequence...	Astrothelium mac...	360	433	92%	1e-94	83.61%	800	LC127383.1
Astrothelium macrocarpum genes for 18S rRNA, ITS1, 5.8S rRNA, ITS2, 28S rRNA, partial and complete sequence...	Astrothelium mac...	360	433	92%	1e-94	83.61%	800	LC127382.1
Astrothelium macrocarpum TNS:YO12392 genes for 18S rRNA, ITS1, 5.8S rRNA, ITS2, 28S rRNA, partial and com...	Astrothelium mac...	356	425	85%	2e-93	85.11%	810	LC573991.1
Astrothelium macrocarpum genes for 18S rRNA, ITS1, 5.8S rRNA, ITS2, 28S rRNA, partial and complete sequence	Astrothelium mac...	355	424	92%	6e-93	83.33%	799	AB759880.1
Astrothelium macrocarpum voucher HMAS-L0146701 small subunit ribosomal RNA gene, partial sequence; internal...	Astrothelium mac...	355	424	92%	6e-93	83.33%	800	MT948054.1
Astrothelium macrocarpum TNS:YO12285 genes for 18S rRNA, ITS1, 5.8S rRNA, ITS2, 28S rRNA, partial and com...	Astrothelium mac...	351	424	91%	7e-92	83.38%	726	LC573990.1
Uncultured Capnodiales clone Hj93 18S ribosomal RNA gene, partial sequence; internal transcribed spacer 1, 5.8S...	uncultured Capno...	332	332	66%	7e-86	85.29%	617	JX317516.1
Marcelaria cumingii genes for 18S rRNA, ITS1, 5.8S rRNA, ITS2, 28S rRNA, partial and complete sequence, isolat...	Marcelaria cumingii	312	312	67%	6e-80	83.12%	555	LC034179.1
Marcelaria cumingii genes for 18S rRNA, ITS1, 5.8S rRNA, ITS2, 28S rRNA, partial and complete sequence, isolat...	Marcelaria cumingii	303	303	67%	3e-77	82.50%	556	LC034180.1
