# Supplementary material for: Morphological and Phylogenetic Characterizations Reveal Five New Species of Astrothelium (Trypetheliales, Ascomycota) from China
Source: J Fungi (Basel). 2022 Sep 22;8(10):994. doi: 10.3390/jof8100994 (PMC9604869; doi:10.3390/jof8100994)
Supplement: Supplementary file 1 [file jof-08-00994-s001.zip › jof-1907393-Supplementary file 6 The phylogenetic tree of ITS.pdf]

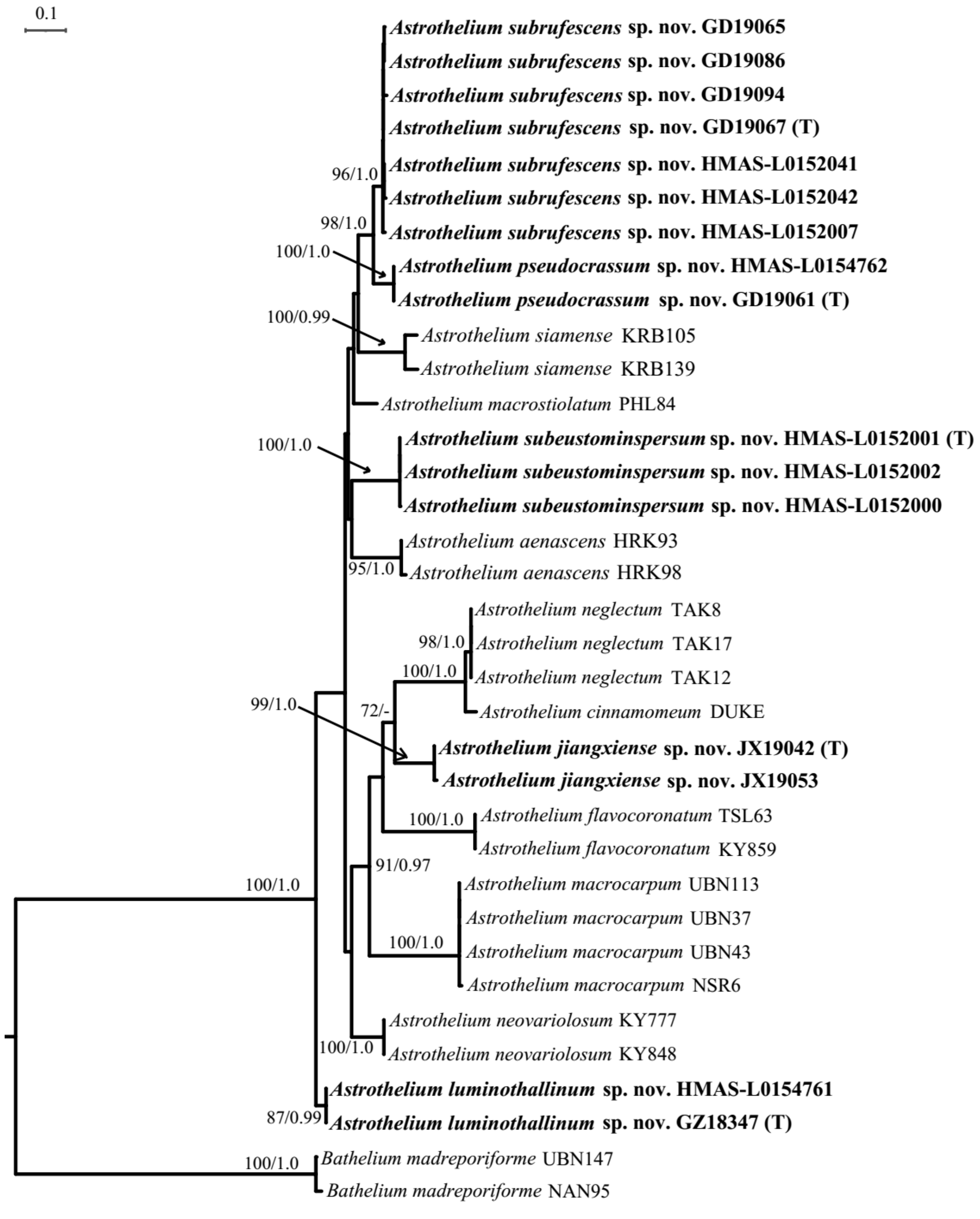

Supplementary 6. ML tree showing the internal phylogeny of *Astrothelium* based on ITS with an alignment length of 455 bp. Maximum likelihood bootstrap support above 70% (left) and Bayesian inference posterior probabilities above 0.95 (right) are shown at nodes (ML–BP/B–PP). Terminals in boldface indicate newly generated sequences for this study. The type specimens in the phylogenetic tree were labeled by letter T.
