# Supplementary material for: Morphological and Phylogenetic Characterizations Reveal Five New Species of Astrothelium (Trypetheliales, Ascomycota) from China
Source: J Fungi (Basel). 2022 Sep 22;8(10):994. doi: 10.3390/jof8100994 (PMC9604869; doi:10.3390/jof8100994)
Supplement: Supplementary file 1 [file jof-08-00994-s001.zip › jof-1907393-Supplementary file 8 TLC test of the new speices Astrothelium jiangxiense.pdf]

We attached the figure below to display the shape of UV+ red spot by TLC in *Astrothelium jiangxiense* sp. nov.

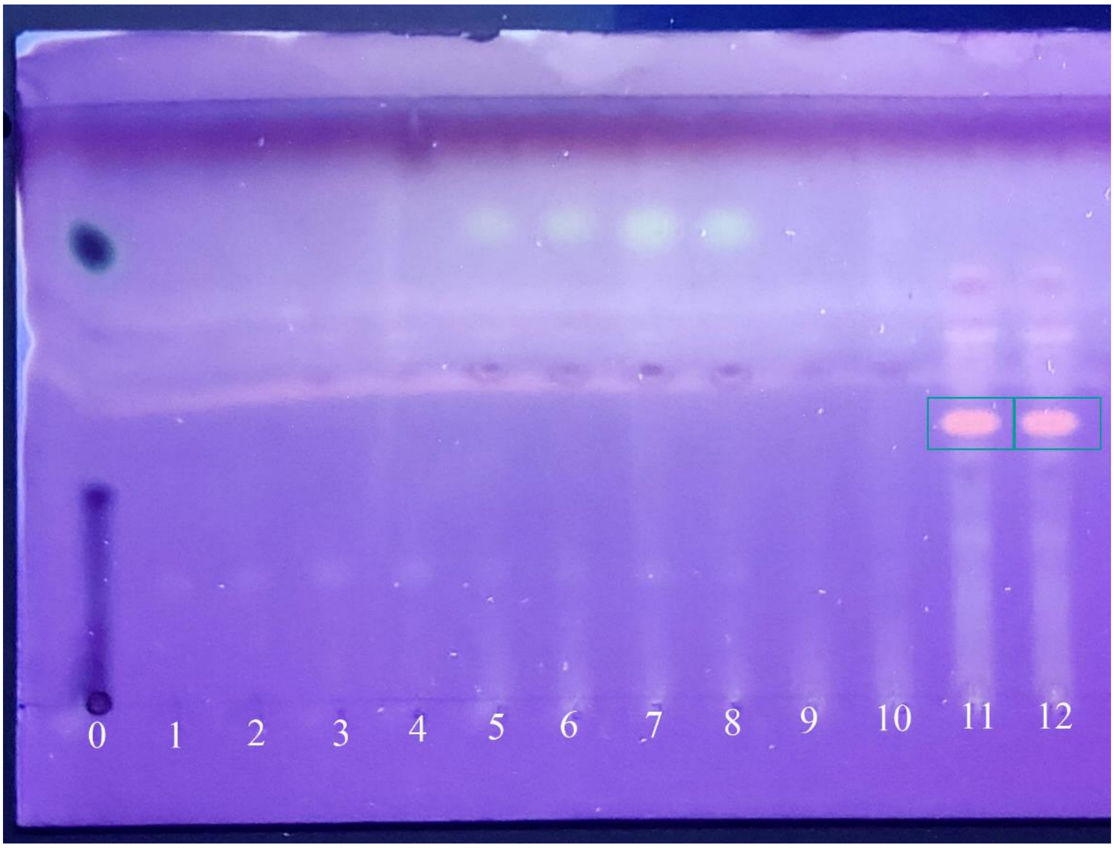

Supplementary 8. TLC test of the new speices *Astrothelium jiangxiense*. 0: standard substance (*Lethariella cladonioides*). 1-2: The bark without thallus of the new species *Astrothelium jiangxiense* as the control (1 is JX19042, and 2 is JX19053); 3-10: Other taxon in the experiment; 11-12: The new species *Astrothelium jiangxiense* (11 is JX19042, and 12 is JX19053). The UV red spot of *Astrothelium jiangxiense* in 11 and 12 were marked.
