# Supplementary material for: Morphological and Phylogenetic Characterizations Reveal Five New Species of Astrothelium (Trypetheliales, Ascomycota) from China
Source: J Fungi (Basel). 2022 Sep 22;8(10):994. doi: 10.3390/jof8100994 (PMC9604869; doi:10.3390/jof8100994)
Supplement: Supplementary file 1 [file jof-08-00994-s001.zip › jof-1907393-Supplementary file 9 World key to the species of Pyrenulaceae and Trypetheliaceae.pdf]

World key to the species of Pyrenulaceae and Trypetheliaceae

André Aptroot[1]

[1]Laboratório de Botânica / Liquenologia, Instituto de Biociências Universidade Federal de Mato Grosso do Sul, Avenida Costa e Silva s/n Bairro Universitário, CEP 79070-900, Campo Grande, Mato Grosso do Sul, Brazil email: andreaptroot@gmail.com

The keys below treat all c. 750 species in the families Pyrenulaceae and Trypetheliaceae. They are a separate print of the keys published in this book:

FELIX SCHUMM & ANDRÉ APTROOT: Atlas of Pyrenulaceae and Trypetheliaceae, Volume 1, pp1-520, ISBN 978-3-7543-9685-8

They are published separately here online so as to give access to a wide audience, also to those who do not buy the book.

General key to the families and some genera of tropical pyrenocarpous lichens
(specimens without ascospores or with only conidia can rarely been identified and are not keyed out; it is important to distinguish septation with thickened cell walls, called distosepta, from simple eusepta; also it is important to observe whether or not the hamathecium filaments = paraphyses are branched; in case one of these characters is not ascertained, try both alternatives)

Please note that **keys A** to **E** mainly contain Pyrenulaceae, and **keys F** to **Ω** are all Trypetheliaceae. The keys look similar to previously published keys, but differ in numerous points, notably additional species and the corrections of mistakes. Each key alternative is given a separate code (like K2b) for easy future reference. Updated known world distributions are indicated for each species.

1a Hamathecium filaments absent or slimy; algae dark green, verrucarioid; thallus usually microsquamulose. Verrucariaceae (corticolous: *Agonimia*, *Endocarpon*, *Placidium*, *Psoroglaena*)
1b Hamathecium filaments present; algae trentepohlioid, or sometimes absent. 2

2a Ascospores simple. 3
2b Ascospores septate. 5

3a Ascospores globose, >50 per ascus; pantropical (Brazil, Papua New Guinea, Solomon Islands). *Melanophloea pacifica* P. James & Vězda
3b Ascospores 1 to 8 per ascus. 4

4a Ascospores ornamented with warts or spines. *Monoblastia*
4b Ascospores not ornamented, with wide, shaped gelatinous sheath: pantropical, but rarely reported (Brazil, Papua New Guinea). *Papilionovela athallina* Aptroot

5a Hamathecium with unbranched paraphyses or hamathecium unclear. 6
5b Hamathecium with branched to anastomosing paraphysoids. 19

6a Ascospores brown. 7
6b Ascospores hyaline. 12

7a Ascospores remaining stuck in mass on top of the ascoma, mazaedioid. Pyrenulaceae (*Mazaediothecium, Pyrgillus*). **Key E**
7b Ascospores discharged. 8

8a Ascospores 1-septate. 9
8b Ascospores more than 1-septate. Pyrenulaceae. 11

9a Ascospores not ornamented, often thick-walled, distoseptate. Pyrenulaceae (*Clypeopyrenis, Distopyrenis, Granulopyrenis, Parapyrenis*). **Key D**
9b Ascospores ornamented, thin-walled. 10

10a Ascospores up to 20 × 8 µm, only in one species larger (up to 30 × 10 µm). *Pseudobogoriella*. **Key X**
10b Ascospores 20–50 × 8–15 µm. *Bogoriella*. **Key Q**

11a Ascospores transversely septate. Pyrenulaceae (mostly *Eopyrenula, Lithothelium, Pyrenula*). **Key B**
11b Ascospores (sub)muriform. Pyrenulaceae (mostly *Anthracothecium, Pyrenula*). **Key A**

12a Ascospores >10 per ascus. *Thelopsis*
12b Ascospores 1 to 8 per ascus. 13

13a Hamathecium a pseudoparenchymatous tissue; ascomata usually in dense groups with common black cover. *Mycoporum*
13b Hamathecium consisting of filaments. 14

ISSN 1868-4173

14a Ascospores thick-walled, distoseptate, either with rather unclear septation or with filiform ascopores). Pyrenulaceae (*Celothelium, Lithothelium*). **Key C**
14b Ascospores thin-walled, euseptate. 15

15a Ascomata in thalline warts with black pulveraceous mass. *Phyllobathelium*
15b Ascomata not in thalline warts with black pulveraceous mass. 16

16a Thallus medulla bright yellow or orange. *Myeloconis*
16b Thallus medualla not bright yellow or orange. 17

17a Ascomata with periphyses in addition to paraphyses. *Topelia*
17b Ascomata without periphyses. 18

18a Ascomata adorned with setae formed of conglutinated hairs. *Trichothelium*
18b Ascomata usually not adorned, but if with setate, these not made of conglutinated hairs. *Porina*

19a Ascospores distoseptate, mostly thick-walled; when realtively thin-walled, either hyaline and with constrictions or brown and ornamented. Trypetheliaceae. 23
19b Ascospores thin-walled, euseptate. 20

20a Hamathecium filaments unbranched between the asci. 21
20b Hamathecium filaments branched. 22

21a Ascospores 1 to 3-septate. Monoblastiaceae (*Acrocordia, Anisomeridium, Megalotremis, Trypetheliopsis*)
22b Ascospores muriform. *Julella*

22a Ascospores hyaline, usually uniseriate in the ascus; conidia with gelatinous appendages. Strigulaceae (*Dichoporis, Flagellostrigula, Strigula, Swinscowia*)
22b Ascospores biseriate; conidia without appendages. Thelenellaceae (*Aspidothecium, Thelenella*)

23a Ascospores remaining hyaline. 24
23b Ascospores becoming brown. 50

24a Ascospores 1-septate. 25
24b Ascospores 3-septate to muriform. 26

25a Ascospores smooth-walled. *Constrictolumina*. **Key R**
25b Ascospores granular ornamented. *Macroconstrictolumina*. **Key T**

26a Thallus ecorticate, usually whitish or greyish. 27
26b Thallus distinctly corticate, olive-green to yellowish brown. 31

27a Hamathecium filaments basally thickened, branched but usually not anastomosing. 28
27b Hamathecium filaments thin, straight, branched and anastomosing to form a net-like structure. 29

28a Ascospores smooth-walled. *Constrictolumina*. **Key R**
28b Ascospores granular ornamented. *Macroconstrictolumina*. **Key T**

29a Ascospores with diamond-shaped lumina. *Pseudopyrenula*. **Key Y**
29b Ascospores with almost rectangular lumina. 30

30a Ascospores transversely septate, or when muriform, ostiole apical. *Polymeridium*. **Key W**
30b Ascospores muriform; ostioles lateral. *Dictyomeridium*. **Key S**

31a Ascospores transversely septate. 32
31b Ascospores muriform. 41

32a Ascospores very large with few (3–5) septa, typically over 100 × 30 µm and up to 190 × 60 µm; septa and walls somewhat thickened but lumina not astrothelioid. *Architrypethelium*. **Key G**
32b Ascospores when 3–5-septate very rarely exceeding 100 × 30 µm and if so, ascospores distinctly astrothelioid, with diamond-shaped lumina. 33

33a Ascospores with diamond-shaped lumina. 34
33b Ascospores with ellipsoid to lentiform or almost rectangular lumina. 38

34a Ascomata strongly prominent to sessile, completely exposed, pure black, somewhat egg-shaped. *Nigrovothelium*. **Key V**
34b Ascomata immersed to erumpent or aggregate in erumpent to sessile pseudostromata. *Astrothelium*. 35

35a Ascomata each with a separate, consistently apical ostiole. 36
35b Ascomata either with a separate, eccentric to lateral ostiole or several ascomata with afus ed ostiole which is then positioned apically relative to the ascomatal cluster but with individual channels originating eccentric or lateral from each individual ascoma or chamber. 37

ISSN 1868-4173

36a Thallus and/or ascomata with external or internal, yellow to red pigment(s) (usually K+ red to purple) and/or with lichexanthone (then UV+ yellow). **Key H**
36b Pigments and lichexanthone absent. **Key I**

37a Thallus and/or ascomata with external or internal, yellow to red pigment(s) (usually K+ red to purple) and/or with lichexanthone (then UV+ yellow). **Key J**
37b Pigments and lichexanthone absent. **Key K**

38a Ascomata aggregate in prominent to sessile, brownish black pseudostromata; ascospores with thin septa and walls. *Bathelium*. **Key P**
38b Ascomata solitary to pseudostromatic but pseudostromata not as above; ascospores with slightly thickened septa. 39

39a Ascomata immersed to erumpent or rarely indistinctly pseudostromatic; pigments mostly absent. 40
39b Ascomata aggregate in distinct, prominent to sessile pseudostromata; internal and/or external pigments usually present. *Trypethelium*. **Key Z**

40a Thallus UV-negative. *Viridothelium*. **Key ω**
40b Thallus UV+ yellow; Brazil. *Exiliseptum ocellatum* (Müll. Arg.) R.C. Harris

41a Ascospores with ellipsoid to lentiform or almost rectangular lumina. 42
41b Ascospores with diamond-shaped lumina. 45

42a Ascomata single. *Architrypethelium submuriforme* Aptroot
42b Ascomata aggregate. 43

43a Ascomata aggregate in prominent to sessile, brownish black pseudostromata. *Bathelium*. **Key P**
43b Ascomata in erumpent, whitish pseudostromata. 44

44a Thallus UV-negative: Panama. *Viridothelium tricolor* Lücking, M.P. Nelsen & N. Salazar
44b Thallus UV+ yellow; Brazil. *Trypethelium muriforme* Aptroot & M.F. Souza

45a Ascomata in prominent to sessile warts covered by a thick layer of yellow-orange or red pigment. *Marcelaria*. **Key U**
45b Ascomata immersed to erumpent, rarely prominent, usually covered by thallus, rarely with a thin layer of pigment pruina. 46

46a Ascomata aggregate in irregular, blackish pseudostromata; ascospores about 50 × 15 µm; Thailand. *Architrypethelium murisporum* Luangsuph., Lumbsch & Sangvichien
46b Ascomata and ascospores not with the above combination of characters. *Astrothelium*. 47

47a Ascomata each with a separate, consistently apical ostiole. 48
47b Ascomata either with a separate, eccentric to lateral ostiole or several ascomata with afus ed ostiole which is then positioned apically relative to the ascomatal cluster but with individual channels originating eccentric or lateral from each individual ascoma or chamber. 49

48a Thallus and/or ascomata with external or internal, yellow to red pigment(s) (usually K+ red to purple) and/or with lichexanthone (then UV+ yellow). **Key L**
48b Pigments and lichexanthone absent. **Key M**

49a Thallus and/or ascomata with external or internal, yellow to red pigment(s) (usually K+ red to purple) and/or with lichexanthone (then UV+ yellow). **Key N**
49b Pigments and lichexanthone absent. **Key O**

50a Ascospores transversely septate. 51
50b Ascospores (sub-)muriform. 56

51a Ascospores with a basal euseptum and the basal cell pale, otherwise distoseptate and brown; neotropical. *Polypyrenula sexlocularis* (Müll. Arg.) D. Hawksw.
51a Ascospores septa all similar. 52

52a Ascospores large, usually over 100 µm long. 53
52b Ascospores small, under 50 µm long. 54

53a Ascospores 3-septate, 25–50 µm broad, about 3–4 times as long as broad. *Architrypethelium*. **Key G**
53b Ascospores 11–15-septate, 25–30 µm broad, about 4–5 times as long as broad; Colombia. *Astrothelium fuscosporum* Soto-Medino, Aptroot & Lücking

54a Ascospores drop-shaped, macrocephalic, with strongly thickened terminal walls and two narrow, rectangular lumina near the center; Azores. *Schummia angulata* (Aptroot & Schumm) Lücking, R. Miranda & Aptroot
54b Ascospores ellipsoid, not macrocephalic. 55

55a Ascospores up to 20 × 8 µm, only in one species larger (up to 30 × 10 µm). *Pseudobogoriella*. **Key X**

ISSN 1868-4173

55b Ascospores 20–50 × 8–15 µm. *Bogoriella*. **Key Q**

56a Ascospores small, under 50 × 20 µm, often with irregularly thickened endospore and halter-shaped lumina. *Bogoriella*. **Key Q**
56b Ascospores very large, 150–400 × 50–140 µm, with outer wall often breaking under pressure. *Aptrootia*. **Key F**

Key A. **Mostly tropical corticolous pyrenocarpous lichens with simple paraphyses and brown, submuriform to muriform, distoseptate ascospores (mostly Pyrenulaceae: *Anthracothecium, Lithothelium* and *Pyrenula*)**

A1a Young ascospores with eusepta only; mature ascospores with scarce endospore and edgy corners. *Anthracothecium*. 2
A1b Septation only or mostly distoseptate, with notably thick endospore layers, in particularly in the corners, and causing the locules to appear rounded. 6

A2a Ascomata simple. 3
A2b Ascomata mostly compound with a joint ostiole. 5

A3a Ostiole apical. 4
A3b Ostiole lateral; pantropical. *Anthracothecium australiense* (Müll. Arg.) Aptroot

A4a Ascospores 2–4/ascus; pantropical. *Anthracothecium macrosporum* (Hepp) Müll. Arg.
A4b Ascospores 6–8/ascus; pantropical. *Anthracothecium prasinum* (Eschw.) R.C. Harris

A5a Ascospores 1–2/ascus; pantropical. *Anthracothecium interlatens* (Nyl.) Aptroot
A5b Ascospores 6–8/ascus; australasian, possibly also african.
Anthracothecium gregale (C. Knight) Aptroot

A6a Ascospores strongly flattened (lozenge-shaped), with two rows of locules. *Sulcopyrenula*. 7
A6b Ascospores not flattened, circular in transverse section. 11

A7a Thallus UV-negative. 8
A7b Thallus UV+ yellow. 9

A8a Hamathecium inspersed; neotropical. *Sulcopyrenula canellae-albae* (Fée) H. Harada
A8b Hamathecium not inspersed; pantropical. *Sulcopyrenula staurospora* (Tuck.) H. Harada

A9a Ascospores with 4 locules. 10
A9b Ascospores with 8 locules; Guyana. *Sulcopyrenula biseriata* Aptroot & Sipman

A10a Ascospores ellipsoidal, c. 2 times as long as wide; Brazil.
Sulcopyrenula cruciata Aptroot
A10b Ascospores nearly globose; neotropical. *Sulcopyrenula subglobosa* (Riddle) Aptroot

A11a Ascospores submuriform, only one or a few of the median locules with longitudinal septa. 12
A11b Ascospores muriform (when ascospores small, logically only few longitudinal septa can be present; when in doubt, start with the first alternative). 25

A12a Ascomata usually fused or compound, when separate, ostioles lateral; ostioles apical or lateral. 13
A12bAscomata separate and simple; ostioles apical. 18

A13a Ascomata fused, in black, exposed groups with several ostioles; ascospores 15–22 µm long; australasian. *Lithothelium hieroglyphicum* Aptroot
A13b Ascomata compound, with several, usually flask-shaped chambers usually immersed in the thallus and with a joint ostiole. 14

A14a Ascospores mostly less than 25 µm long. 15
A14b Ascospores mostly over 25 µm long. 17

A15a Thallus with pseudocyphellae; ascospores 15–19 µm long; Guyana. *Pyrenula infrastroidea* Aptroot & Sipman
A15b Thallus without pseudocyphellae. 16

A16a Ascospores red-brown, 14–23 µm long; eastern palaeotropical (Australia to Japan). *Lithothelium nanosporum* (C. Knight) Aptroot
A16b Ascospores grey-brown, 17–20(–25) µm long; neotropical. *Pyrenula subvariabilis* Aptroot & Sipman

A17a Ascospores up to 32 µm long; australasian. *Pyrenula subumbilicata* (C. Knight) Aptroot
A17b Ascospores 45–65 µm long; neotropical. *Pyrenula erumpens* R.C. Harris

A18a Ascospores 13-35(-40) µm long. 19
A18b Ascospores 34–72 µm long. 24

A19a Ascospores 13-17 µm long. 20

ISSN 1868-4173

A19b Ascospores 22–35(-40) µm long. 21

A20a Ascospores with little endospore formation, lumina almost angular; on wood, pacific. *Pyrenographa irregularis* (Wehmeyer) R.C. Harris
A20b Ascospores much thickened, with rounded lumina; Florida. *Distopyrenis submuriformis* R.C. Harris

A21a Thallus probably without algae; temperate. *Requienella seminuda* (Pers.) Boise
A21b Thallus superficial, with copious algae. 22

A22a Ascospores 22–40 µm long; overmature ascospores with reddish content before shiveling; pantropical. *Pyrenula seminuda* (Müll. Arg.) Sipman & Aptroot
A22b Ascospores 23–35 µm long; overmature spores all shrivelled. 23

A23a Ascospores 23–35 µm long; hamathecium not inspersed; eastern palaeotropical. *Pyrenula gibberulosa* (Vain.) Aptroot
A23b Ascospores 23–33 µm long; hamathecium inspersed; eastern palaeotropical (India). *Pyrenula darjeelingensis* Jagadeesh Ram & G.P. Sinha

A24a Ascospores with pointed ends; hamathecium not inspersed; eastern palaeotropical (Australia to Japan). *Pyrenula subvariolosa* (C. Knight) Aptroot
A24b Ascospores with rounded ends; hamathecium inspersed; neotropical. *Pyrenula novemseptata* Vain.

A25a Ascomata and/or thallus or medulla with yellow, orange or red anthraquinone pigments; pigments K+ pink to purplish. 26
A25b Anthraquinones absent; thallus K– or yellowish. 32

A26a Thallus warts internally with soft orange medulla; Philippines.
Pyrenula endocrocea Aptroot
A26b Yellow, orange or red pigment on outside. 27

A27a Ascomata and/or thallus red. 28
A27b Ascomata and/or thallus yellow to orange. 30

A28a Ostiole apical; hamathecium inspersed; neotropical. *Pyrenula cruentata* (Müll. Arg.) R.C. Harris
A28b Ostiole lateral; hamathecium not inspersed. 29

A29a Ascomata partly fused with joint ostioles; ascospores with up to 6 locules per segment; caribbean. *Pyrenula kermesina* R.C. Harris

A29b Ascomata solitary; ascospores with up to 2 locules per segment; pacific. *Pyrenula palmarum* (Krempelh.) R.C. Harris

A30a Ascospores 10–23 µm long, with 3 primary septa. 31
A30b Ascospores 23–35 µm long, with 5–7 primary septa; neotropical.
Pyrenula ochraceoflavens (Nyl.) R.C. Harris

A31a Thallus red-orange; ascospores 9–18 µm long; Brazil. *Pyrenula aurantiacorubra* Aptroot & M. Cáceres
A31b Thallus yellow to orange; ascospores 10–23 µm long; pantropical.
Pyrenula ochraceoflava (Nyl.) R.C. Harris
(with small ascospores with two layers of locules; pacific: *Pyrenula ochraceoflava* var. *pacifica* P.M. McCarthy)

A32a Ostioles lateral. 33
A32b Ostioles apical. 41

A33a Ascospores <70 µm long. 34
A33b Ascospores >70 µm long. 36

A34a Ascomata at least partly with more than one chamber and with joint ostiole. 35
A34b Ascomata all simple and single, with separate ostioles; ascospores 15–33 µm long; pantropical (El Salvador, India). *Pyrenula microspora* (Nagarkar & Patw.) Upreti

A35a Ascospores 25–45 µm long; pantropical. *Pyrenula astroidea* (Fée) R.C. Harris
A35b Ascospores 45–70 µm long; pantropical. *Pyrenula ravenelii* (Tuck.) R.C. Harris

A36a Ascospores 2/ascus, 135–200 µm long; pantropical. *Pyrenula lyoni* (Zahlbr.) Aptroot
A36b Ascospores 4–8/ascus. 37

A37a Ascospores 25–110 µm long. 38
A37b Ascospores 85–135 µm long. 40

A38a Ascospores 25–31 µm long; Guyana. *Pyrenula triangularis* Aptroot & Sipman
A38b Ascospores 50–110(–135) µm long. 39

A39a Ascospores 50–90 µm long; pantropical (El Salvador, India). *Pyrenula ceylonensis* (Ajay Singh & Upreti) Aptroot
A39b Ascospores 70–135 µm long; W. Europe, Madagascar & Macaronesia). *Pyrenula hibernica* (Nyl.) Aptroot

A40a Ascomata mostly with several chambers; pantropical. *Pyrenula schiffneri* (Zahlbr.) Aptroot
A40b Ascomata often with single chamber; neotropical. *Pyrenula chilensis* (Fée) R.C. Harris

A41a Ascospores <25 µm long. 42
A41b Ascospores >25 µm long. 46

A42a Hamathecium inspersed. 43
A42b Hamathecium not inspersed. 44

A43a Ascospores 13–18 µm long, with basal cilium; Mauritius. *Pyrenula muriciliata* Diederich & Ertz
A43b Ascospores 20–25 µm long, without cilium; Borneo. *Pyrenula borneensis* Aptroot

A44a Thallus UV+yellow; pantropical. *Pyrenula confinis* (Nyl.) R.C. Harris
A44b Thallus UV-negative. 45

A45a Ascospores 15–22 µm long; pantropical. *Pyrenula parvinuclea* (Meyen & Flot.) Aptroot
A45b Ascospores 7–10 µm long; India. *Pyrenula nanospora* (Ajay Singh) Upreti

A46a Old ascospores containing orange oil before shriveling; pantropical. *Pyrenula breutelii* (Müll. Arg.) Aptroot
A46b Old ascospores without orange oil, directly shriveling. 47

A47a Ascospores >80 µm long, mostly 2/ascus. 48
A47b Ascospores <80 µm long, mostly 4–8/ascus. 53

A48a Hamathecium inspersed, ascospores 90–200 µm long. 49
A48b Hamathecium not inspersed. 50

A49a Thallus UV+ yellow, with lichexanthone; neotropical. *Pyrenula xanthoglobulifera* Aptroot, Lücking & M. Cáceres
A49b Thallus UV-negative, without lichexanthone; pantropical. *Pyrenula globifera* (Eschw.) Aptroot

A50a Thallus without pseudocyphellae, ascospores 80–140(–155) µm long; pantropical. *Pyrenula platystoma* (Müll. Arg.) Aptroot
A50b Thallus with pseudocyphellae. 51

A51a Ascospores 80–180 µm long, 2-8/ascus. 52

A51b Ascospores 205–350 µm long, 1/ascus; Guyana. *Pyrenula monospora* Aptroot & Sipman

A52a Ascospores 80–110 µm long, 2-8/ascus; Hawaii. *Pyrenula neosandwicensis* Aptroot
A52b Ascospores 115–180 µm long, 2/ascus; pantropical. *Pyrenula duplicans* (Nyl.) Aptroot

A53a Hamathecium inspersed. 54
A53b Hamathecium not inspersed. 55

A54a Ascospores 2/ascus, 55–75 µm long; Brazil. *Pyrenula bispora* Aptroot & M. Cáceres
A54b Ascospores 8/ascus, 30–45 µm long; pantropical (India, Brazil). *Pyrenula sublaevigata* (Patw. & Makhija) Upreti

A55a Locules relatively large and angular, with up to 6 between 2 primary septa; pantropical. *Pyrenula leucostoma* Ach.
A55b. Locules mostly round, at least in the central part of the ascospore with more than 6 between 2 primary septa. 56

A56a Ascospores <50 µm long. 57
A56b Ascospores >50 µm long. 60

A57a Ascospores 25–35 µm long; palaeotropical. *Pyrenula welwitschii* (Upreti & Ajay Singh) Aptroot
A57b Ascospores >35 µm long. 58

A58a Ascospores 11–15 µm wide; pantropical. *Pyrenula thelomorpha* Tuck.
A58b Ascomata 14–22 µm wide. 59

A59a Old ascospores containing colourless oil before shriveling; neotropical. *Pyrenula oleosa* R.C. Harris
A59b Old ascospores without oil, directly shriveling when old; neotropical. *Pyrenula dissimulans* (Müll. Arg.) R.C. Harris

A60a Ascomata deeply immersed in bark below the thallus, not visible, only ostioles visible; Brazil. *Pyrenula abditicarpa* Aptroot & M. Cáceres
A60b Ascomata more-or-less exposed. 61

A61a Ascospores with rounded ends; pantropical. *Pyrenula pyrenuloides* (Mont.) R.C. Harris

ISSN 1868-4173

A61b Ascospores with pointed ends; pantropical. *Pyrenula papillifera* (Nyl.) Aptroot

Key B. **Corticolous pyrenocarpous lichens with simple paraphyses and brown, transversely 3- or more septate distoseptate ascospores (mostly Pyrenulaceae: *Eopyrenula*, *Lithothelium* and *Pyrenula*)**

B1a Ascospores 3-loculate. 2
B1b Ascospores 4- or more-loculate. 4

B2a Ascospore septation strongly asymmetrical; Madagascar. *Lacrymospora parasitica* Aptroot
B2b Ascospore septation symmetrical. 3

B3a Ascomata aggregated in grey to black pseudostromata; pantropical. *Pyrenula lineatostroma* Aptroot
B3b Ascomata dispersed, not stromatoid; Brazil. *Pyrenula biseptata* Aptroot & M. Cáceres

B4a Ascospores nearly only euseptate, locules rectangular, at most a bit rounded in the corners, end cells often paler than middle cells. 5
B4b Ascospores clearly distoseptate, locules more or less rounded or diamond-shaped. 17

B5a Ascospores more than 5 times as long as wide; South Africa. *Pyrenowilmsia wilmsiana* (Zahlbr.) Aptroot
B5b Ascospores less than 4times as long as wide. 6

B6a Ascospores only 4-loculate; macroconidia 3-septate or unknown. 7
B6b Ascospores 4–8-loculate; macroconidia either 1–7-septate or unknown. 12

B7a Ascoma wall dense. 8
B7a Ascoma wall cellular. *Eopyrenula*. 10

B8a Ascospores smooth, 14–17 µm long; neotropical. *Pyrenula tenuisepta* R.C. Harris
B8b Ascospores verrucose, 30–47 µm long. *Bogoriella*. 9

B9a Ascomata at least partly fused with a more or less joint ostiole; Japan; pantropical. *Bogoriella collospora* (Vain.) Aptroot & Lücking
B9b Ascomata all single; Australasian; pantropical. *Bogoriella queenslandica* (Müll. Arg.) Aptroot & Lücking

B10a Ascospores 4–5 µm wide; New world temperate. *Eopyrenula parvispora* R.C. Harris & Aptroot
B10b Ascospores 5.5–7.5 µm wide. 11

B11a Ascospores mostly <15 µm long; Atlantic (W. Europe). *Eopyrenula avellanae* Coppins
B11b Ascospores mostly >15 µm long; Atlantic (W. Europe). *Eopyrenula grandicula* Coppins

B12a Ascospores 4(–6)-loculate, macroconidia 1-septate; Old world temperate. *Eopyrenula leucoplaca* (Wallr.) R.C. Harris
B12b Ascospores (4–)6–8-loculate; macroconidia 3–7-septate or unknown. 13

B13a Ascospores mostly >15 µm long. *Eopyrenula*. 14
B13b Ascospores mostly over 25 µm long. 15

B14a Ascospores >6 µm wide, macroconidia (5–)7-septate; Atlantic Europe. *Eopyrenula septemseptata* Coppins
B14b Ascospores <6 µm wide, macroconidia 3(–4)-septate; New world temperate. *Eopyrenula intermedia* Coppins

B15a Ascospores mostly 37–47 µm long, constricted at the septa; Europe. *Blastodesmia nitida* A. Massal.
B15b Ascospores 25–35 µm long. *Requienella*. 16

B16a Ascospores 5-7-septate, rarely 3-septate; temperate. *Requienella seminuda* (Pers.) Boise
B16b Ascospores mostly 3-septate; temperate. *Requienella fraxini* Jaklitsch & Voglmayr

B17a Ascospores red brown and locules at least becoming rounded when older (species on rock not keyed out here). *Lithothelium*. 18
B17b Ascospores grey to brown, rarely red brown and then locules angular or diamond-shaped. *Pyrenula*. 22

B18a Thallus UV+ yellow; Costa Rica. *Lithothelium fluorescens* Aptroot & Sipman
B18b Thallus UV-negative. 19

B19a Ascospores 4-loculate. 20
B19b Ascospores 8-loculate. 21

B20a Ascospores 15–20 µm long; eastern palaeotropical. *Lithothelium decumbens* (Müll. Arg.) Aptroot

B20b Ascospores 25–40 µm long; northern temperate. *Lithothelium phaeosporum* (R.C. Harris) Aptroot

B21a Ascospores 30–40 µm long; northern temperate. *Lithothelium septemseptatum* (R.C. Harris) Aptroot
B21b Ascospores 55–80 µm long; New world temperate. *Lithothelium macrosporum* (R.C. Harris) Aptroot

B22a Ostioles pointing in various directions, mostly eccentric to lateral; ascomata sometimes with several chambers connected to joint ostioles. 23
B22b Ostioles apical or, when eccentric, all pointing in the same direction; ascomata with one chamber or each chamber with own ostiole. 48

B23a Ascospores 6–16-loculate. 24
B23b Ascospores 4-loculate. 27

B24a Ascospores 6-loculate. 25
B24b Ascospores 10–16-loculate. 26

B25a Ascospores 42–55 µm long, 8/ascus; neotropical (Colombia, Florida). *Pyrenula pleiomera* (Nyl.) Zahlbr.
B25b Ascospores 60–65 µm long, 4/ascus; Venezuela. *Pyrenula tetraspora* Aptroot & Sipman

B26a Ascospores 10–14-loculate, 65–90 × 17–22 µm; ascomata with single chamber; Brazil. *Pyrenula fusispora* (Malme) Aptroot
B26b Ascospores 12–16-loculate, 50–70 × 4.5–6 µm; ascomata with 5–15 stellately fused chambers; Japan. *Pyrenula tokyensis* (Müll. Arg.) H. Harada

B27a Thallus UV+ yellow. 28
B27b Thallus UV-negative. 29

B28a Ascospores 21–23 µm long; Hawaii. *Pyrenula hawaiiensis* Aptroot
B28b Ascospores 32–45 µm long; Brazil. *Pyrenula crassiuscula* (Malme) Aptroot

B29a Terminal locules directly against the exospore wall. 30
B29b Terminal locules separated from the exospore wall by endospore thickening. 34

B30a Ascomata at least partly with 2-7 chambers with joint ostioles; neotropical. *Pyrenula subgregantula* Müll. Arg.
B30b Ascomata single. 31

B31a Ascospores 13–25 µm long. 32
B31b Ascospores 25–65 µm long. 33

B32a Ascospores 16–25 µm long; pantropical. *Pyrenula circumfiniens* Vain.
B32b Ascospores 13–16 µm long; neotropical. *Pyrenula elliptica* Müll. Arg.

B33a Ascospores 45–65 µm long; neotropical. *Pyrenula erumpens* R.C. Harris
B33b Ascospores 25–35 µm long; pantropical. *Pyrenula cuyabensis* (Malme) R.C. Harris

B34a Ostiole with red colour (inconspicuous character!). 35
B34b Ostiole without red colour. 36

B35a Ostiole outside with a red ring; New world temperate. *Pyrenula wetmorei* R.C. Harris
B35b Ostiole red brown to bright red, eccentric, single; Brazil. *Pyrenula rubrolateralis* Aptroot & M. Cáceres
B35c Ostiole red, fused; Brazil. *Pyrenula sanguineoastroidea* Aptroot

B36a Ascomata mostly with several chambers and a joint ostiole. 37
B36b Ascomata nearly all single. 43

B37a Ascospores 10–24 µm long. 38
B37b Ascospores >25 µm long. 41

B38a Ascospores 10–12 µm long; Argentina. *Pyrenula punctoleucotrypa* Aptroot, L.I. Ferraro & M. Cáceres
B38b Ascospores mostly 15–20 µm long. 39

B39a Ascospores 17–20 x 5.5–6.5 µm; hamathecium inspersed with colourless oil globules instead of clear; Brazil. *Pyrenula inspersicollaris* Aptroot & M. Cáceres
B39b Ascospores 12–24 µm long; pantropical. 40

B40a Thallus partly wine-red to purplish by K+ purple pigment; ascospores 16–21 µm long; Brazil. *Pyrenula violaceastroidea* C.O. Mendonça, Aptroot & M. Cáceres
B40b Thallus not pigmented; ascospores 12–24 µm long; pantropical. *Pyrenula septicollaris* (Eschw.) R.C. Harris

B41a Ascoma chambers deeply immersed; ascospores 23–27 µm long; Brazil. *Pyrenula leptaleoides* Aptroot & M. Cáceres
B41b Ascoma chambers emergent; ascospores mostly over 25 µm long. 42

B42a Ascospores 25–35 µm long; pantropical. *Pyrenula cubana* (Müll. Arg.) R.C. Harris

ISSN 1868-4173

B42b Ascospores 30–45 µm long; pantropical. *Pyrenula cryptothelia* (Müll. Arg.) Aptroot & Etayo

B43a Ascospores 13–17 µm long; hamathecium inspersed; USA (Florida). *Pyrenula wheeleri* R.C. Harris
B43b Ascospores >17 µm long; hamathecium not inspersed. 44

B44a Ascospores 17–28 µm long. 45
B44b Ascospores 28–45 µm long (compare also *Pyrenula minarum*, with partly eccentric ostioles). 47

B45a Ascospores with dark bands of euseptum material between the locules, 19–22 µm long; Brazil. *Pyrenula viridipyrgilla* Aptroot & M. Cáceres
B45b Ascospores without dark bands, 18–30 µm long. 46

B46a Ascospores with at least one pointed end; almost cosmopolitan. *Pyrenula acutispora* Kalb & Hafellner
B46a Ascospores with rounded ends; pantropical. *Pyrenula microtheca* R.C. Harris

B47a Ascospores 35–45 µm long; pantropical. *Pyrenula adacta* Fée
B47b Ascospores 28–32 µm long; probably pantropical (only known from Brazil and Papua New Guinea). *Pyrenula gahavisukana* Aptroot

B48a Ascospores with 2-8 or many hyaline cilia at the ends (not to be confused with germ tubes which are one per lumen). 49
B48b Ascospores without cilia. 50

B49a Ascospores 4-loculate; Australasian (Papua New Guinea). *Pyrenula ciliata* Aptroot
B49b Ascospores 6–8-loculate; neotropical. *Pyrenula hirsuta* Etayo

B50a Ascospores at least partly with more than 3 septa, or with 3 septa and long tails at one or both ends, seemingly more than 4-loculate. 51
B50b Ascospores all distinctly 4-loculate. 65

B51a Ascospores at least seemingly 5–8-loculate. 52
B51b Ascospores 8–18-loculate, more than 4 times as long as wide. 59

B52a Ascospore with tails c. 1 µm wide and not septate; palaeotropical. 53
B52b Ascospore without tails, at most pointed; neotropical. 54

B53a Ascospores with tails at both ends; Australia. *Pyrenula bicuspidata* Müll. Arg.
B53b Ascospores with tail at basal end only; Oceania. *Pyrenula mastigophora* Aptroot

B54a Ascospores more than 4 times as long as wide; neotropical. 55
B54b Ascospores les than 4 times as long as wide. 56

B55a Ascospores more than 4 times as long as wide; hamathecium clear. *Pyrenula melanophthalma* (Mont.) Trevis.
B55b Ascospores 30–37 x 3–4 µm; thallus UV+ yellow instead of UV–; hamathecium inspersed with colourless oil globules. *Pyrenula musaespor*a Aptroot & M. Cáceres

B56a Ascospores <30 µm long. 57
B56b Ascospores >29 µm long. 58

B57a Old ascospores with orange oil; thallus often with pseudocyphellae; pantropical. *Pyrenula sexlocularis* (Nyl.) Müll. Arg.
B57b Ascospores without orange oil; thallus without pseudocyphellae; South Africa. *Pyrenula wilmsiana* Müll. Arg.

B58a Ascospores 29–35 µm long; hamathecium not inspersed; Australasian (Papua New Guinea). *Pyrenula sexluminata* Aptroot
B58b Ascospores 30–55 µm long; hamathecium inspersed; pantropical. *Pyrenula caracasana* Müll. Arg.

B59a Ascospores with a long tail at one end; Pacific (Mariana Islands). *Pyrenula flagellata* H. Harada
B59b Ascospores without tails. 60

B60a Ascospores 8–18-loculate. 61
B60b Ascospores (6–)8-loculate. 64

B61a Thallus without pseudocyphellae. 62
B61b Thallus with pseudocyphellae. 63

B62a Ascospores 13–18-loculate; eastern palaeotropical (Ryukyu Islands, Japan). *Pyrenula cylindrica* Kashiw.
B62b Ascospores 8–13-loculate; USA (Florida). *Pyrenula clavatispora* Common & Aptroot

B63a Ascospores 8–12-loculate; eastern palaeotropical (India). *Pyrenula subcylindrica* Jagadeesh Ram & Upreti
B63b Ascospores 12–18-loculate, 75–90 × 4.5–6.5 µm, gradually attenuated toward the ends; New Caledonia. *Pyrenula filiformis* Aptroot

ISSN 1868-4173

B64a Ascospores 30–38 µm long; pantropical. *Pyrenula montagnei* Müll. Arg.
B64b Ascospores 53–70 µm long; Australasian (New Zealand), also neotropical? *Pyrenula moniliformis* (C. Knight) Müll. Arg.

B65a Ascospores more than 4 times as long as wide; Brazil. *Pyrenula fusoluminata* Aptroot
B65b Ascospores less than 4 times as long as wide. 66

B66a Ascospores mostly >50 µm long. 67
B66b Ascospores mostly <50 µm long. 73

B67a Locules not in a straight line, somewhat zig-zag; Brazil. *Pyrenula hoehneliana* Zahlbr.
B67b Locules in a straight line or ascospores bent, not zig-zag. 68

B68a Terminal locules directly against the exospore wall. 69
B68b Terminal locules separated from the exospore wall by endospore thickening; thallus with pseudocyphellae or crystalline papillae. 71

B69a Thallus UV+yellow; neotropical. *Pyrenula praelucida* (Mont.) Trevis.
B69b Thallus UV-negative. 70

B70a Hamathecium not inspersed; pantropical. *Pyrenula rockii* Zahlbr.
B70b Hamathecium inspersed; neotropical. *Pyrenula subpraelucida* Müll. Arg.

B71a Hamathecium inspersed with orange oil globules, K+ greenish, not clear; ascospores 60–75 x 24–28 µm; thallus without papillae; Venezuela. *Pyrenula aurantioinspersa* Aptroot & Sipman
B71b Hamathecium not inspersed. 72

B72a Ascospores (45–)50–60 µm long; thallus without papillae but with pseudocyphellae; pantropical. *Pyrenula immissa* (Stirt.) Zahlbr.
B72b Ascospores 75–90 µm long; thallus with crystalline papillae; Costa Rica. *Pyrenula montocensis* Lücking

B73a Locules not in a straight line, somewhat to strongly zig-zag. 74
B73b Locules in a straight line, not zig-zag. 75

B74a Thallus UV+ yellow; pantropical. *Pyrenula aggregans* Vain.
B74b Thallus UV-negative; pantropical. *Pyrenula papilligera* (Leight.) Müll. Arg.

B75a Ascomata and/or thallus with yellow, orange or red anthraquines on the outside. 76
B75b Ascomata and thallus without external pigments. 97

B76a Terminal locules directly against the exospore wall. 77
B76b Terminal locules separated from the exospore wall by endospore thickening. 80

B77a Ascomata at least partly yellow; hamathecium not inspersed; neotropical and pacific (Panama, Costa Rica, Puerto Rico, Hawaii). *Pyrenula luteopruinosa* Etayo & Aptroot
B77b Ascomata or thallus orange or red; hamathecium inspersed. 78

B78a Ascomata red; thallus UV-negative; Australasian (Lord Howe Island). *Pyrenula howeana* Aptroot
B78b Thallus or ascomata orange, thallus UV+ yellow. 79

B79a Ascomata orange; ascospores 17–20 µm long; eastern palaeotropical (Korea). *Pyrenula sipmanii* Aptroot & K.H. Moon
B79b Thallus orange; ascospores 19–22 µm long; eastern palaeotropical (Thailand). *Pyrenula aurantiopileata* Aptroot

B80a Thallus yellow (in shade) to orange (in full light). 81
B80b Thallus and/or ascomata at least partly pink, red or lilac. 82

B81a Ascospores ca. 25-40 µm long, not plicate; found in mangrove; neotropical. *Pyrenula cerina* Eschw.
B81b Ascospores 55-65 µm long, longitudinally plicate; Chile. *Pyrenula plicata* Sipman & Aptroot

B82a Thallus and/or ascomata at least partly red. 83
B82b Thallus lilac; Brazil. *Pyrenula lilacina* C.O. Mendonça, Aptroot & M. Cáceres
B82c Thallus salmon pink; hamathecium inspersed; ascospores 24–27 × 13–16 µm; Brazil. *Pyrenula salmonea* Aptroot

B83a Hamathecium not inspersed. 84
B83b Hamathecium inspersed with colourless oil globules. 91

B84a Ascomata aggregated in pseudostromata. 85
B84b Ascomata mostly separate. 88

B85a Ascospores 15–18(-20) µm long. 86
B85b Ascospores 20–24 µm long; ascomata densely aggregated in well-delimited, bright red pseudostromata; Brazil. *Pyrenula sanguinea* Aptroot, M. Cáceres & Lücking

B86a Ascomata covered by red thallus; Brazil. *Pyrenula cinnabarina* Aptroot, E.L. Lima & M. Cáceres
B86b Ascomata covered by red pigment only. 87

B87a Ascospores 15–17 µm long; Costa Rica. *Pyrenula rubroanomala* Aptroot & Lücking
B87b Ascospores 15–18(–20) µm long; Brazil. *Pyrenula coccinea* C.O. Mendonça, Aptroot & M. Cáceres

B88a Ascospores 16–19 µm long. 89
B88b Ascospores 21–28 µm long. 90

B89a Red colouration only in tissue arond the ostiole; neotropical. *Pyrenula rubrostoma* R.C. Harris
B89b Red coloration extending over the thallus; Brazil. *Pyrenula rubronitidula* Aptroot & M. Cáceres

B90a Thallus extensively carmine-red-stained; Brazil. *Pyrenula rubroacutispora* Aptroot
B90b Only ostioles of (immersed) ascomata red; Caribbean. *Pyrenula sanguineostiolata* Aptroot & Mercado Diaz

B91a Ascomata aggregated in pseudostromata; ascospores 12–15 x 6–8µm; Brazil. *Pyrenula reginae* E.L. Lima, Aptroot & M. Cáceres
B91b Ascomata not aggregated. 92

B92a Ascospores about 15–18 µm long. 93
B92b Ascospores mostly over 19 µm long. 94

B93a Ascospores about 19–22 µm long. 95
B93b Ascospores about 19–22 µm long. 96

B94a Thallus orange-brown to red, ostioles black; Brazil. *Pyrenula rubromamillana* E.L. Lima, Aptroot & M. Cáceres
B94b Only ostioles pigmented, red; Brazil. *Pyrenula rubrostigma* Aptroot & M. Cáceres

B95a Thallus dark reddish to orange-brown, K+ crimson; ascomata hemispherical, 0.4–0.7 mm diam.; eastern palaeotropical (Java). *Pyrenula rubrojavanica* Aptroot
B95b Thallus orange, K+ purple; ascomata low-conical, 0.7–1 mm diam.; Brazil. *Pyrenula aurantiacothallina* C.O. Mendonça, Aptroot & M. Cáceres

B96a Ascospores 25–29 x 10–12 µm; thallus with red spots or lines; ascomata immersed in thallus, 0.2–0.4 mm diam.; Caribbean. *Pyrenula sanguineomeandrata* Aptroot & Mercado Diaz
B96b Ascospores 27–35 µm long; thallus with red patches or completely red; ascomata erumpent, c. 0.4–0.8 mm diam.; pantropical. *Pyrenula cruenta* (Mont.) Vain.

B97a Ascomata mostly aggregated, with fused walls but with separate ostioles. 98
B97b Ascomata mostly simple, only aggregated as by chance when crowded (if difficult to decide, start with the first alternative). 112

B98a Old ascospores with red oil; Puerto Rico. *Pyrenula concastroma* R.C. Harris
B98b Old ascospores without red oil. 99

B99a Terminal locules directly against the exospore wall; Australasian (Papua New Guinea). *Pyrenula pyrenastrospora* Aptroot
B99b Terminal locules separated from the exospore wall by endospore thickening. 100

B100a Hamathecium inspersed with colourless oil globules. 101
B100b Hamathecium not inspersed. 103

B101a Ascospores 16–20 µm long. 102
B101b Ascospores 25–40 µm long; ostioles sometimes partly eccentric; pantropical. *Pyrenula minarum* Vain.

B102a Ascomata hemispherical, 0.3–0.5 mm diam.; ascospores 16–18 µm long; Brazil. *Pyrenula paraminarum* Aptroot & M. Cáceres
B102b Ascomata conical with flattened tip, 0.4–1.2 mm diam.; ascospores 17–20 µm long; Argentina. *Pyrenula inspersoleucotrypa* Aptroot, L.I. Ferraro & M. Cáceres

B103a Ascospores mostly <20 µm long. 104
B103b Ascospores mostly >21 µm long. 108

B104a Ascospores with dark bands between the locules; USA (Florida). *Pyrenula atrolaminata* R.C. Harris
B104b Ascospores without dark bands. 105

B105a Ascomata not in dense stromata but more loosely aggregated, the individual ascomata often well recognizable, conical, with distinct ostioles. 106
B105b Ascomata in dense, well delimited stromata, subglobose with indistinct, black ostioles. 107

ISSN 1868-4173

B106a Ascospores 11–13 x 3.5–5 µm; Brazil. *Pyrenula aggregataspistea* Aptroot & M. Cáceres
B106b Ascospores 15–18(–22) µm long; Brazil. *Pyrenula infraleucotrypa* Aptroot & M. Cáceres

B107a Ascospores mostly >15 µm long; ascomata in dense stromata; pantropical. *Pyrenula anomala* (Ach.) Vain.
B107b Ascospores mostly <15 µm long; pantropical. *Pyrenula arthoniotheca* Upreti

B108aAscospores mostly <25 µm long. 109
B108b Ascospores mostly >25 µm long. 110

B109a End locules elongated; Costa Rica. *Pyrenula subsoluta* (Müll. Arg.) Aptroot
B109b All locules more or less rounded; eastern palaeotropical. *Pyrenula leucotrypa* (Nyl.) Upreti

B110a Thallus with pseudocyphellae; neotropica. l111
B110b Thallus without pseudocyphellae; eastern palaeotropical (Sri Lanka). *Pyrenula zeylanica* Upreti & Ajay Singh

B111a Thallus with pseudocyphellae; Cuba. *Pyrenula wrightii* (Müll. Arg.) R.C. Harris
B111b Thallus with pseudocyphellae, ascomata slightly fused, in groups of few ascomata which are hardly touching eachother; Brazil. *Pyrenula diamantinensis* C.O. Mendonça, Aptroot & M. Cáceres

B112a Thallus ecorticate, whitish. 113
B112b Thallus corticate (may be difficult to observe in poor specimens; when in doubt try the first alternative first). 118

B113a Thallus UV+yellow. 114
B113b Thallus UV–. 115

B114a Ascospores 18–21 µm long; pantropical. *Pyrenula cocoes* Müll. Arg.
B114b Ascospores 14–17 µm long; Brazil. *Pyrenula xanthinspersa* Aptroot & M. Cáceres

B115a Hamathecium inspersed; pantropical (Brazil, Philippines). *Pyrenula albothallina* Vain.
B115b Hamathecium not inspersed. 116

B116a Thallus white to grey, dull; ascomata <0.7 mm diam.; pantropical. *Pyrenula microcarpa* Müll. Arg.

B116b Thallus cream to silvery or inapparent, smooth; temperate. 117

B117a Ascospores <18 µm long; hamathecium K-negative; pycnidia present. Known from temperate regions on the northern hemisphere and reported from Chile, so probably cosmopolitan in temperate regions. *Pyrenula coryli* A. Massal.
B117b Ascopores >18 µm long; hamathecium sometimes KOH+ blue (fleeting), pycnidia absent; temperate northern hemisphere. *Pyrenula laevigata* (Pers.) Arnold

B118a Old ascospores with orange oil. 119
B118b Old ascospores without orange oil. 120

B119a Ascospores 25–35 µm long; pantropical. *Pyrenula bahiana* Malme
B119b Ascospores (30–)35–51 µm long; eastern palaeotropical. *Pyrenula thailandica* Aptroot

B120a Terminal locules all directly against the exospore wall (if anthraquinones present on thallus and/or ascomata, compare B75). 121
B120b Terminal locules mostly (at least in mature ascospores) separated from the exospore wall by endospore thickening. 142
(See also *P. minae*, *P. pyrgillospora* and *P. xanthominuta* which are keyed out via 142 because they have a thick outer wall suggesting an apical endospore layer. Their ascospores resemble *Pyrgillus javanicus*)

B121a Thallus UV+yellow. 122
B121b Thallus UV-negative. 124

B122a Hamathecium not inspersed; pantropical. *Pyrenula cocoes* Müll. Arg.
B122b Hamathecium inspersed. 123

B123a Thallus olivaceous with thick cortex; northern temperate. *Pyrenula pseudobufonia* (Rehm) R.C. Harris
B123b Thallus pale grey, with thin cortex; neotropical (Guyana, Venezuela). *Pyrenula guyanensis* Sipman & Aptroot
(*Pyrenula obvoluta* (Nyl.) R.C. Harris may be an older name, but in the lectotype the fertile warts are only 0.6–0.8 mm wide and details of hamathecium and ascospores are missing)

B124a Hamathecium inspersed (in some cases only in the upper part). 125
B124b Hamathecium not inspersed. 136

B125a Ascospores with elongated end locules. 126
B125b Ascospores with all locules more or less rounded to angular. 129

ISSN 1868-4173

B126a Ascospores 20–25 µm long; pantropical (compare also *Pyrenula acutalis* which is only inspersed in the upper part). *Pyrenula maravalensis* Vain.
B126b Ascospores >25 µm long. 127

B127a Ascospores with attenuated, horn-like apices, (21–)25–29(–32) µm long; Brazil. *Pyrenula cornutispora* Aptroot & M. Cáceres
B127b Ascospores without horn-like apices. 128

B128a Ascospores 26–30 µm long; Australasian (Papua New Guinea). *Pyrenula rinodinospora* Aptroot
B128b Ascospores 35–50 µm long; eastern palaeotropical (India). *Pyrenula kurzii* Ajay Singh & Upreti

B129a Ascospores all <16 µm long; pantropical. *Pyrenula cayennensis* Müll. Arg.
B129b Ascospores partly >16 µm long. 130

B130a Ascospores <40 µm long. 132
B130b Ascospores >40 µm long. 135

B131a Ascospores partly >30 µm long. 132
B131b Ascospores all <30 µm long. 133

B132a Ascospores with black granular material around the ends; Australasian (Papua New Guinea). *Pyrenula grossa* Aptroot
B132b Ascospores without black granular material; eastern palaeotropical (Borneo, Sri Lanka). *Pyrenula fulva* (Krempelh.) Müll. Arg.

B133a Thallus with patches of orange brown colouration which are KOH+purple; known from temperate regions on the northern hemisphere and South Africa, so probably cosmopolitan. *Pyrenula occidentalis* (R.C. Harris) R.C. Harris
B133b Thallus without coloured, KOH+purple patches. 134

B134a Hamathecium only inspersed in the upper part; neotropical. *Pyrenula acutalis* R.C Harris
B134b Hamathecium totally inspersed; pantropical. *Pyrenula fetivica* (Krempelh.) Müll. Arg.

B135a Ascospores 42–50 µm long, mostly straight; eastern palaeotropical (India). *Pyrenula subcamptospora* Upreti
B135b Ascospores 45–52 µm long, mostly curved; neotropical. *Pyrenula cryptostoma* (Nyl.) Müll. Arg.

B136a End locules elongated. 137
B136b All locules more-or-less rounded to angular. 139

B137a Ascospores 20–29 µm long; ascomata <0.7 mm diam. 138
B137b Ascospores 27–40 µm long; ascomata >0.7 mm diam.; pantropical. *Pyrenula vernicosa* (Krempelh.) Müll. Arg.

B138a Ascospores 20–29 µm long; ascomata 0.5–0.7 mm diam.; eastern palaeotropical. *Pyrenula finitima* Müll. Arg.
B138b Ascospores 20–29 µm long; ascomata <0.5 mm diam.; pantropical. *Pyrenula approximans* (Krempelh.) Müll. Arg.

B139a Ascomata >2 mm diam.; eastern palaeotropical (Philippines). *Pyrenula irosina* Vain.
B139b Ascomata <2 mm diam. 140

B140a Ascospores 11–13 µm long; palaeotropical (Seychelles). *Pyrenula infracongruens* Aptroot & Schumm
B140b Ascospores >13 µm long. 141

B141a Ascospores 14–16 µm long; eastern palaeotropical. *Pyrenula mastophoriza* (Nyl.) Zahlbr.
B141b Ascospores at least partly >16 µm long; pantropical. *Pyrenula nitidula* (Bres.) R.C. Harris

B142a Hamathecium inspersed. 143
B142b Hamathecium not inspersed. 162

B143a Hamathecium inspersed with coloured particles. 144
B143b Hamathecium inspersed with colourless oil globules. 146

B144a Hamathecium inspersed with orange, K-negative crystals; Sri Lanka. *Pyrenula multicolorata* Weerakoon & Aptroot
B144b Hamathecium inspersed with red or yellow oil globules. 145

B145a Hamathecium inspersion red; ascospores 13-16 µm long; Venezuela. *Pyrenula rubroinspersa* Aptroot & Sipman
B145b Hamathecium inspersion yellow; ascospores 29-35 µm lon; Ecuador. *Pyrenula flavoinspersa* Aptroot & Sipman

ISSN 1868-4173

B146a Central ascospore locules elongated; Northern temperate. *Pyrenula subelliptica* (Tuck.) R.C. Harris
B146b Central ascospore locules transversely lenticular to rounded. 147

B147a Ascospores constricted at the central septum; litle endospore thickening between the outer lumina and the wall; Costa Rica. *Pyrenula minae* Aptroot & Lücking
B147b Ascospores not constricted, at most becoming constricted when postmature. 148

B148a Ascomata mostly <0.7 mm diam. 149
B148b Ascomata mostly >0.7 mm diam. 153

B149a Ascospores 13–18 µm long; neotropical. *Pyrenula laetior* Müll. Arg.
B149b Ascospores >18 µm long. 150

B150a Ascospores about 18-25 µm long. 151
B150b Ascospores >27 µm long. 152

B151a Ascospores 18–20 µm long; thallus and ascomata without any anthraquinone; eastern palaeotropical (Singapore). *Pyrenula subglabrata* (Nyl.) Müll. Arg.
B151b Ascospores 18–25 µm long; thallus and/or ascomata with patches of rusty anthraquinone; known from temperate regions on the northern hemisphere and South Africa, so probably cosmopolitan. *Pyrenula occidentalis* (R.C. Harris) R.C. Harris

B152a Ascospores 25–31 µm long; thallus with pseudocyphellae; Brazil. *Pyrenula supralaetior* C.O. Mendonça, Aptroot & M. Cáceres
B152b Ascospores 27–50 µm long; thallus without pseudocyphellae; eastern palaeotropical (India, Sri Lanka). *Pyrenula oculata* Ajay Singh & Upreti

B153a Ascospores mostly 10-20 µm long. 154
B153b Ascospores mostly >20 µm long. 156

B154a Ascospores mostly 10–14 µm long, up to 4 µm wide; Brazil. *Pyrenula inframamillana* Aptroot & M. Cáceres
B154b Ascospores mostly >15 µm long. 155

B155a Ascospores mostly 14-17 µm long and about half as wide, biseriate in the ascus; pantropical. *Pyrenula castanea* (Eschw.) Müll. Arg.
B155b Ascospores mostly 17–21 µm long and about one third as wide, uniseriate in the ascus; pantropical *Pyrenula mamillana* (Ach.) Trevis.

B156a Ascospores <30 µm long. 157

B156b Ascospores 30–45 µm long. 159

B157a Ascoma chamber deeply immersed in the bark; only ostioles visible, often with red-brown pigment; Brazil. *Pyrenula celaticarpa* Aptroot & M. Cáceres
B157b Ascomata erumpent, at least partially visible as black dots. 158

B158a Ascospores rounded, uniseriate in the ascus; pantropical. *Pyrenula massariospora* (Starb.) R.C. Harris
B158b Ascospores at least at one end pointed, biseriate in the ascus; neotropical. *Pyrenula acutalis* R.C. Harris

B159a Ascomata conical, emergent. 160
B159b Ascomata globose to hemiglobose. 161

B160a Ascomata conical, emergent, sides spreading; eastern palaeotropical. *Pyrenula interducta* (Nyl.) Zahlbr.
B160a Ascospores without black granular material, and with thin apical walls bulging at the spore tips; palaeotropical (Seychelles). *Pyrenula supracongruens* Aptroot & Schumm

B161a Ascomata globose, immersed; neotropical. *Pyrenula mastophoroides* (Nyl.) Zahlbr.
B161b Ascomata hemispherical, prominent; eastern palaeotropical. *Pyrenula ocellulata* Wijeyaratne, Lücking & Lumbsch

B162a Thallus UV+ yellow (when material well preserved, sometimes only part of the thallus reacting). 163
B162b Thallus UV-negative or greenish/whitish reflecting. 167

B163a Ascospores mostly >24 µm long. 164
B163b Ascospores mostly <24 µm long. 165

B164a Ascospores 25–40 µm long, without black granules; Costa Rica (also reported from China). *Pyrenula andina* Aptroot
B164b Ascospores 36–45 µm long, with black granules at the tips; temperate America. *Pyrenula caryae* R.C. Harris

B165a Thallus brownish with thick cortex; ascomata not in distinct thalus warts. 166
B165b Thallus whitish with thin cortex; ascomata immersed in distinct thallus warts; ascospores 15–22 µm long, ellipsoidal, not pointed; neotropical. *Pyrenula micromma* (Mont.) Trevis.

ISSN 1868-4173

(*Pyrenula obvoluta* (Nyl.) R.C. Harris & Aptroot is most probably a separate species, characterized by not-pointed ascospores)

B166a Ascospores 9–11 µm long, ellipsoid; pantropical (Australia, Guyana). *Pyrenula xanthominuta* Aptroot
B166b Ascospores 14–21 µm long, fusiform, pointed; cosmopolitan. *Pyrenula dermatodes* (Borrer) Schaer.
(somewhat variable, especially in UV-reaction, but always with pointed ascospores and thallus at least partly covering the ascomata. *Pyrenula shirabeicola* Kurok. & S. Nakan. is probably a further synonym. The oldest name found for specimens that are not reacting with UV is *Pyrenula subtrahens* (Nyl.) Müll. Arg.)

B167a Ascospores mostly >25 µm long. 168
B167b Ascospores mostly <25 µm long. 181

B168a Ascospores 36–45 µm long, with or without black granules at the tips. 169
B168b Ascospores <40 µm long, without black granules at the tips. 170

B169a Ascospores 36–45 µm long, with black granules at the tips; temperate America. *Pyrenula caryae* R.C. Harris
B169b Ascospores 36–45 µm long, without black granules at the tips; pantropical. *Pyrenula subducta* (Nyl.) Müll. Arg.

B170a End locules elongated, ascospores 24–29 µm long; Virgin Islands. *Pyrenula expectata* R.C. Harris
B170b End locules not elongated. 171

B171a Growing on rock, ascospores with dark bands between the locules, 25–30 µm long; Brazil. *Pyrenula quarzitica* Aptroot
B171b Growing on bark, ascospores without dark bands. 172

B172a Ascomata mostly >0.7 mm diam. 173
B172b Ascomata mostly <0.7 mm diam. 176
(*Pyrenula lilacina* with lilac-colored thallus keys out here when the pigment is not observed)

B173a Thallus with pseudocyphellae; temperate regions of the old world. *Pyrenula macrospora* (Degel.) Coppins & P. James
B173b Thallus without pseudocyphellae. 174

B174a Ascospores 27–32 µm long, when mature dark brown and constricted at the central septum instead of pale grey brown and constricted at the locules; Guyana. *Pyrenula minoides* Aptroot & Sipman
B174b Ascospores pale greybrown and constricted at the locules. 175

B175a Ascospores 24–29 µm long, thallus thick; eastern palaeotropical (Papua New Guinea, Juan Fernandez). *Pyrenula media* Aptroot
B175b Ascospores 29–40 µm long, thallus thin; eastern palaeotropical. *Pyrenula complanata* (Mont.) Trevis.

B176a Ascospores with diamond-shaped locules; temperate regions on both hemispheres. *Pyrenula chlorospila* (Nyl.) Arnold
B176b Ascospores with somewhat rounded or quadrangular locules. 177

B177a Ascospores with dark granules between the locules; temperate America and Japan. *Pyrenula macounii* R.C. Harris
B177b Ascospores without dark bands. 178

B178a Ascospores 32–42 µm long; temperate America and Japan. *Pyrenula punctella* (Nyl.) Trevis.
B178b Ascospores mostly 25–37 µm long; tropical. 179

B179a Central locules of ascospores much longer than wide; ascomata superficial, hemispherical; Guyana. *Pyrenula perfecta* Aptroot & Sipman
B179b Central locules of ascospores not longer than wide. 180

B180a Central locules much wider than long, ascomata conical, emergent, thallus without pseudocyphellae; pantropical. *Pyrenula mastophora* (Nyl.) Müll. Arg.
B180b Central locules more-or-less rounded; ascomata somewhat rounded, often partly immersed in the thallus; thallus often with (generally sparse) pseudocyphellae; pantropical. *Pyrenula quassiicola* (Fée) Fée

B181a Ascospores mostly 21–25 µm long. 182
B181b Ascospores mostly <21 µm long. 189

B182a Ascomata c. 3–4 mm diam.; Japan. *Pyrenula gigas* Zahlbr.
B182b Ascomata <3 mm diam. 183

B183a Ascomata with red, KOH+purple crystals inside. 184
B183b Ascomata without red crystals. 185

ISSN 1868-4173

B184a Ascomata <0.5 mm diam.; temperate regions of Europe and Asia. *Pyrenula nitidella* (Flörke ex Schaer.) Müll. Arg.
B184b Ascomata >0.5 mm diam.; temperate regions of Europe and Asia. *Pyrenula nitida* (Weigel) Ach.

B185a Ascospores with angular diamond-shaped locules; temperate North and South America. *Pyrenula micheneri* R.C. Harris
B185b Ascospores with rounded or quadrangular locules. 186

B186a Ascospores with bands of dark granules between the locules, without much endospore thickening between the outer lumina and the wall; pantropical. *Pyrenula pyrgillospora* Aptroot
B186b Ascospores without bands of dark granules. 187

B187a Thallus with dense pseudocyphellae; postmature ascospores longitudinally plicate; Chile. *Pyrenula maritima* Sipman & Aptroot
B187b Thallus without pseudocyphellae; ascospores not regularly plicate when postmature. 188

B188a Ascospores with at least one pointed end; temperate northern hemisphere, extending to the tropics. *Pyrenula acutispora* Kalb & Hafellner
B188b Ascospores with rounded ends; eastern palaeotropical (India). *Pyrenula submastophora* Ajay Singh & Upreti

B189a Ascospores 7–10 µm long; Iran. *Pyrenula minutissima* Aptroot, Valadbeigi & Sipman
B189b Ascospores >10 µm long. 190

B190a Ascospores mostly <15 µm long. 191
B190b Ascospores mostly >15 µm long. 195

B191a Ascomata >0.7 mm diam.; pantropical. *Pyrenula atropurpurea* (Eschw.) Müll. Arg.
B191b Ascomata <0.7 mm diam. 192

B192a Ascospores 6–8 µm wide; pantropical. *Pyrenula brunnea* Fée
B192b Ascospores 3–6 µm wide. 193

B193a Ascospores 3.5–4.0 µm wide, rhomboidal; Brazil. *Pyrenula rhomboidea* Aptroot & M. Cáceres
B193b Ascospores 4–6 µm wide. 194

B194a Ascospores 4–6 µm wide; pantropical. *Pyrenula aspistea* (Ach.) Ach.
B194b Ascospores 5–6 µm wide; like P. aspistea but with immersed ascomata; Brazil. *Pyrenula minutispora* Aptroot & M. Cáceres

B195a Thallus at least partly 0.3 mm thick. 196
B195b Thallus thinner. 197

B196a Ascospores at least partly constricted at the septa; Australasian (Papua New Guinea). *Pyrenula montana* Aptroot
B196b Ascospores not constricted; neotropical (Puerto Rico, Brazil). *Pyrenula psoriformis* Zahlbr.

B197a Ascomata >0.7 mm diam. 198
B197b Ascomata <0.7 mm diam. 201

B198a Thallus with pseudocyphellae; New Zealand. *Pyrenula deliquescens* (C. Knight) Müll. Arg.
B198b Thallus without pseudocyphellae. 199

B199a Ascospores pale reddish brown instead of pale grey-brown; Brazil. *Pyrenula mattickiana* Sipman & Aptroot
B199b Ascospores pale grey-brown. 200

B200a Locules rounded; eastern palaeotropical (India). *Pyrenula scutata* (Stirt.) Zahlbr.
B200b Locules angular; pantropical. *Pyrenula balia* (Krempelh.) R.C. Harris

B201a Ascospores with distinct inner endospore layer around locules; neotropical (Florida, Louisiana). *Pyrenula rubrostoma* R.C. Harris
B201b Ascospores without extra endospore layers. 202

B202a Ascospores with dark bands between the locules; North America and Asia. *Pyrenula confoederata* R.C. Harris
B202b Ascospores without dark bands. 203

B203a Ascomata <0.4 mm diam.. 204
B203b Ascomata reaching >0.4 mm diam.; pantropical (UV-negative material of *Pyrenula dermatodes* keys out here as well; The oldest name found for such specimens that are not reacting with UV is *Pyrenula subtrahens* (Nyl.) Müll. Arg.). *Pyrenula aggregata* (Fée) Fée

B204a Thallus pale grey, with thin cortex and filled with crystals, not oily or olivaceous; Brazil. *Pyrenula albonigra* Aptroot, D.S. Andrade & M. Cáceres

B204b Thallus olivaceous, oily. 205

B205a Ascospores 18–20 µm long; palaeotropical. *Pyrenula tristissima* Vain.
B205b Ascospores <18 µm long; neotropical. *Pyrenula minor* Fée

Key C. Pyrenocarpous lichens with simple paraphyses and septate, hyaline, distoseptate ascospores

(*Celothelium, Lithothelium*; temperate *Celothelium* species are not keyed out as they are dubious; all saxicolous *Lithothelium* species are keyed out here as well to make the treatment of all Pyrenulaceae complete, even if some of them have have brown ascospores)

C1a Ascospores filiform. *Celothelium*. 2
C1b Ascospores ellipsoid to fusiform. *Lithothelium*. 7

C2a Ascomata sideways fused. 3
C2b Ascomata solitary or with joined ostioles. 4

C3a Ascomata sideways grouped in dense, often linear, clusters; neotropical. *Celothelium cinchonarum* (Vain.) Vain.
C3b Ascomata in small groups; temperate. *Celothelium ischnobelum* (Nyl.) M.B. Aguirre

C4a Ostiole lateral to eccentric, fused or not. 5
C4b Ostiole apical, ascomata solitary. 6

C5a Ascomata less than 0.5 mm wide; neotropical. *Celothelium aciculiferum* (Nyl.) Vain.
C5b Ascomata over 0.5 mm wide; Korea. *Celothelium longisporum* K.H. Moon & Aptroot
(here also keys out from India: *Lithothelium filisporum* (Patw., Makhija & D. Rane) Kr.P. Singh & G.P. Sinha, which could well be a Celothelium)

C6a Without yellow pigment; neotropical. *Celothelium dominicanum* (Müll. Arg.) Aguirre
C6b With yellow pigmentation; Bermuda. *Celothelium lutescens* F. Berger & Aptroot

C7a Ascospores colourless. 8
C7b Ascospores red-brown to brown; on rock (species on bark are in keys A and B). 25

C8a Thallus on bark; ascospores 3-septate. 9
C8b Thallus on rock. 21

C9a Ascospores 4/ascus; Thailand. *Lithothelium quadrisporum* Aptroot
C9b Ascospores mostly 8/ascus. 10

C10a Ascomata with fused ostioles only or with fused ostioles and fused walls. 11
C10b Ascomata solitary. 14

C11a Ascomata with fused ostioles only; thallus epiphloeodic, brownish to green; ascospores 12–15(–18) × 4–6 µm; pantropical. *Lithothelium obtectum* (Müll. Arg.) Aptroot
C11b Ascomata with fused ostioles and fused walls. 12

C12a Thallus endophloeodic, brownish to grey; ascospores 18–23 × (6–)7–10 µm; pantropical. *Lithothelium illotum* (Vain.) Aptroot
C12b Thallus epiphloeodic, brownish to green. 13

C13a Ascospores 18–23 × (6–)8–11 µm; neotropical. *Lithothelium paraguayense* Müll. Arg.
C13b Ascospores 16–18 × 7–8 µm; neotropical. *Lithothelium fugax* (Müll. Arg.) Aptroot

C14a Thallus epiphloeodic, brownish green to greenish. 15
C14b Thallus endophloeodic, whitish to brownish grey. 17

C15a Ascospores 10–12 × 4–4.5 µm; Florida. *Lithothelium microsporum* R.C. Harris
C15b Ascospores larger. 16

C16a Ascospores 16–19 × 5.5–6.5 µm; South Africa. *Lithothelium almbornii* Aptroot
C16b Ascospores 27–37 × 12–17 µm; pantropical (Brazil, China). *Lithothelium grossum* Aptroot

C17a Hymenium inspersed with oil droplets; ascospores (9–)10–14(–17) × (3–)4–5 µm; Japan. *Lithothelium japonicum* H. Harada
C17b Hymenium not inspersed. 18

C18a Terminal lumina much smaller than the central lumina. 19
C18b Terminal lumina only slightly smaller than the central lumina. 20

C19a Ascospores 14–20(–24) × 6–8(–12) µm; pantropical. *Lithothelium hyalosporum* (Nyl.) Aptroot
C19b Ascospores 18–31 × 10–20 µm; Australia. *Lithothelium quiescens* P.M. McCarthy

C20a Paraphyses simple; ascospores 18–23 × (6–)7–10 µm; pantropical. *Lithothelium illotum* (Vain.) Aptroot

C20b Paraphyses branched and anastomosing; ascospores 17–25 × 8.5–12 µm; India. *Lithothelium insulare* Makhija & Adaw.

C21a Ascospores 4–8-septate, 20–25(–30) × 3–5·5(–6) µm; Bahamas. *Lithothelium bahamense* Riddle
C21b Ascospores 3-septate. 22

C22a Ascomata with fused ostioles and fused walls; thallus endolithic. 23
C22b Ascomata solitary; thallus at least partly epilithic. 24

C23a Thallus violet-grey; hamathecium IKI+ blue to orange; asci with rounded ocular chamber; ascospores 13–17(–20) × 3·5–5(–6) µm; neotropical. *Lithothelium cubanum* Müll. Arg.
C23b Thallus greenish grey; hamathecium IKI negative; asci with sagittiform ocular chamber; ascospores 16–19 × 5–7 µm; Falklands. *Lithothelium falklandicum* (Nyl.) Aptroot

C24a Ostioles apical; asci without ocular chamber; ascospores 17–20 × 6–8 µm; Eurasian temperate. *Lithothelium triseptatum* (Nyl.) Aptroot
C24b Ostioles lateral; asci with rounded ocular chamber; ascospores 15–18 × 6–7 µm; Hawaii. *Lithothelium hawaiiense* Aptroot

C25a Ascospores ornamented with spines, 14–19 × 6–7·5 µm; China. *Lithothelium echinatum* Aptroot
C25b Ascospores smooth. 26

C26a Thallus epilithic, rimose to areolate; ascospores 18–28 × 8–14 µm; Australia. *Lithothelium austropacificum* P.M. McCarthy
C26b Thallus endolithic; ascospores 20–26 × 6–8 µm. 27

C27a Ascospore lumina all rounded: New Zealand. *Lithothelium australe* Aptroot & H. Mayrhofer
C27b Ascospore lumina partly diamond-shaped and connected; Bermuda. *Lithothelium bermudense* F. Berger, LaGreca & Aptroot

Key D. Tropical pyrenocarpous lichens with simple paraphyses and 1-septate, distoseptate, brown ascospores (mostly Pyrenulaceae: *Clypeopyrenis, Distopyrenis, Granulopyrenis, Parapyrenis*)

(all are rare or rarely collected and rarely studied; at least the sequenced *Granulopyrenis* is basal in or sister to the Pyrenulaceae)

D1a ascomata conical, distinctly flattened, superficial, 0.6–1.2 mm diam.; ostioles apical, black, obconical; ascospores brown, biloculate, 6–8 × 2–2.5 µm, ellipsoidal; neotropical. *Clypeopyrenis microsperma* (Müll. Arg.) Aptroot
D1b Ascospores larger. 2

D2a Ascospores with double wall, with a layer of conspicuous granules inside. *Granulopyrenis*. 3
D2b Ascospores without internal granules. 7

D3a Ascomata grouped under a stroma; Brazil. *Granulopyrenis nigrescens* (Müll. Arg.) R.C. Harris
D3b Ascomata single. 4

D4a Thallus not blackened around the ascocarps; ostiole apical, asci with rounded ocular chamber; South Africa. *Granulopyrenis macrocarpoides* (Zahlbr.) Aptroot
D4b Thallus blackened around the ascocarps. 5

D5a Ostiole lateral; asci with rounded ocular chamber; on bark; on driftwood; USA. *Granulopyrenis hymnothora* Aptroot
D5b Ostiole apical to eccentric; asci without ocular chamber. 6

D6a Ostiole partly eccentric; Caribbean. *Granulopyrenis portoricensis* (Petrak) Y. Z. Wang, Aptroot & K. D. Hyde
D6b Ostiole apical; Hong Kong. *Granulopyrenis seawardii* Aptroot & Sipman

D7a Thallus lichenicolous; Papua New Guinea. *Parapyrenis lichenicola* Aptroot & Diederich
D7b Thallus not lichenicolous. 8

D8a Thallus on driftwood; Papua New Guinea. *Parapyrenis maritima* Aptroot
D8b Thallus on bark. 9

D9a Ascomata grouped under a stroma; neotropical (Brazil, Puerto Rico). *Distopyrenis composita* R.C. Harris
D9b Ascomata single. 10

D10a Thallus with violet tinge; Australia. *Distopyrenis violacea* Aptroot
D10b Thallus brownish or whitish. 11

D11a Ascospores with elongated lumina. 12
D11b Ascospores with rounded or angular lumina. 13

D12a hamathecium IKI + blue; ascocarps flattened, simple or aggregated; Bermuda. *Parapyrenis elongata* Aptroot
D12b hamathecium IKI negative; ascocarps not flattened, simple; neotropical. *Parapyrenis guayaca* (Fée) Aptroot

D13a Ascospores with an euseptum within the septum. 14
D13b Ascospores only distoseptate with relatively small lumina. 15

D14a Lumina angular; thallus whitish grey to invisible, not blackened around the ascocarps; USA and Caribbean. *Distopyrenis americana* Aptroot
D14b Lumina rather rounded; thallus pinkish grey, blackened around the ascocarps; Mexico and Caribbean. *Distopyrenis fuliginosa* Aptroot

D15a Ascospores with large, rounded lumina; Australia. *Parapyrenis conica* Aptroot
D15b Ascospores with angular lumina, with much thickened endosporium formation, thus lumina relatively small. 16

D16a Ascomata flattened; Java. *Parapyrenis aurora* (Zahlbr.) Aptroot
D16b Ascomata conical; French Guiana. *Distopyrenis pachyspora* Aptroot

Key E. Pyrenocarpous lichens with mazaedioid ascomata (Pyernulaceae: *Mazaediothecium, Pyrgillus*)

E1a Ascoma wall not carbonized; ascospores amassing in the large ostioles of the cup-like ascomata, either1- septate or cruciate submuriform. *Mazaediothecium*. 2
E1b Ascoma wall carbonized; ascospores amassing in a cup-like structure on top of the closed ascomata, transversely 1-3-septate. *Pyrgillus*. 6

E2a Ascomata red; Papua New Guinea. *Mazaediothecium rubiginosum* Aptroot
E2b Ascomata black. 3

E3a Ascospores 1-septate. 4
E3b Ascospores cruciate submuriform. 5

E4a Thallus corticate; photobiont trebouxioid; Brazil. *Mazaediothecium serendipiticum* Aptroot

E4b Thallus not corticate; photobiont trentepohlioid; French Guiana. *Mazaediothecium uniseptatum* Aptroot

E5a Thallus UV+ yellow; neotropical. *Mazaediothecium album* Aptroot
E5b Thallus UV-negative; Malaysia. *Mazaediothecium mohamedii* H. Harada & Yoshik. Yamam.

E6a Ascospores 1-septate. 7
E6b Ascospores 3-septate. 9

E7a Mazaedium pruina golden-yellow; pantropical. *Pyrgillus indicus* (Kremp.) Aptroot
E7b Mazaedium pruina white. 8

E8a Ascospores with pale ends, red brown; Australia. *Pyrgillus fuscus* Aptroot
E8b Ascospores with dark ends, brown; pantropical. *Pyrgillus cubanus* Nyl.

E9a Thallus UV−; mazaedium pruina orange, KOH+ purple; ascospores 10–14 × 5–7 µm; Cambodia and China. *Pyrgillus cambodiensis* Kashiw., K.H. Moon & Aptroot
E9b Thallus UV+ yellow, with lichexanthone. 10

E10a Mazaedium pruina white, KOH−; ascospores 10–13 × 6.0–8.5 µm; pantropical. *Pyrgillus javanicus* (Mont. & Bosch) Nyl.
E10b Mazaedium pruina orange or red, KOH+ orange, reddish violet or violet. 11

E11a Mazaedium pruina red, KOH+ reddish violet, UV−; ascospores 15–20(–23) × 7.5–10.0 µm; India. *Pyrgillus tibellii* Kr. P. Singh & Pushpi Singh
E11b Mazaedium pruina red but KOH+ violet or orange, UV+ red; ascospores 13.0–17.5×5.0–7.5 µm. 12

E12a Mazaedium pruina orange, KOH+ violet; ascospores 13–16 × 6.0–7.5 µm, c. 2 times as long as wide; Brazil. *Pyrgillus aurantiacus* Aptroot & M. Cáceres
E12b Mazaedium pruina red, KOH+ orange; ascospores 15.0–17.5×5.0–6.5 µm, c. 2.5–3 times as long as wide; Brazil. *Pyrgillus rufus* Aptroot & M. Cáceres

Key F. *Aptrootia* species

F1a Thallus on bark, corticate, green, bullate; ascospores ornamented; Eastern palaeotropical. *Aptrootia elatior* (Stirt.) Aptroot

F1b Thallus on soil and bryophytes, not corticate, grey, cartilaginous, smooth; ascospores smooth. 2

F2a Ascospores 170–230 × 40–70 µm; amphipacific (Costa Rica, Papua New Guinea, Solomon Islands). *Aptrootia terricola* (Aptroot) Lücking, L. Umaña & Chaves
F2b Ascospores 150–360(–400) × 65–140 µm; Australia. *Aptrootia robusta* (P.M. McCarthy & Kantvilas) Aptroot

Key G. *Architrypethelium* species with transversely septate ascospores

G1a Ascospores remaining hyaline 2
G1b Ascospores soon becoming brown 5

G2a Ascomata sessile, very large (up to 3 mm diam.); Brazil. *Architrypethelium grande* (Kremp.) Aptroot & Lücking
G2b Ascomata immersed or in erumpent prominent thallus warts, smaller (usually not exceeding 1 mm diam.). 3

G3a Ascomata completely immersed between bullate thallus warts; Brazil. *Architrypethelium lauropaluanum* Lücking, M.P. Nelsen & Marcelli
G3b Ascomata erumpent to prominent, partly thallus-covered. 4

G4a Thallus UV+ yellow; neotropical. *Architrypethelium hyalinum* Aptroot
G4b Thallus UV–; Bolovia. *Architrypethelium penuriixanthum* Flakus & Aptroot

G5a Ostiole eccentric; ascomata sometimes with fused ostioles; neotropical. *Architrypethelium nitens* (Fée) Aptroot
G5b Ostiole apical; ascomata solitary. 6

G6a Hamathecium inspersed; Colombia. *Architrypethelium columbianum* (Nyl.) Aptroot & Lücking
G6b Hamathecium clear; pantropical. *Architrypethelium uberinum* (Fée) Aptroot

Key H. *Astrothelium* species with ascospores transversely septate, ostiole apical, secondary substances present on thallus and/or ascoma

H1a Lichexanthone present; thallus and/or ascomata UV+ yellow; pigment(s) present or absent. 2

H1b Lichexanthone absent; pigment(s) always present, thallus and ascomata usually K+ red to purple and UV+ orange or red. 21

H2a Pigments present in thallus and/or ascomata; lichexanthone on thallus; ascomata pseudostromatic, in irregular black lines. 3
H2b Pigments absent; lichexanthone on thallus and/or ascomata; ascomata variable. 4

H3a Ascospores 3-septate, small (18–26 µm long); ascomata with external orange pigment, scattered laterally along pseudostromata; neotropical. *Astrothelium neogalbineum* (R.C. Harris) Aptroot & Lücking
H3b Ascospores (3–)5-septate, large (90–115 µm long); ascomata with external purplish-red pigment, scattered laterally along pseudostromata; Brazil. *Astrothelium pictum* Aptroot

H4c Ascospores 9–11-septate; Brazil. *Astrothelium pseudodermatodes* Aptroot
H4a Ascospores 3–9-septate. 5

H5a Ascospores 5–9-septate. 6
H5b Ascospores 3-septate. 7

H6a Lichexanthone on ascomata; ascomata solitary to irregularly confluent, erumpent, covered by thallus; eastern palaeotropical. *Astrothelium cinereorosellum* (Kremp.) Aptroot & Lücking
H6b Lichexanthone on pseudostromata; ascomata distinctly pseudostromatic; Fiji. *Astrothelium fijiense* Lücking, Naksuwankul & Lumbsch

H7a Hamathecium inspersed; ascospores small (15–30 µm long). 8
H7b Hamathecium clear; ascospores small to medium-sized (15–70 µm long). 12

H8a Lichexanthone on ascomata only, absent from thallus; ascomata solitary to irregularly confluent, largely immersed and covered by thallus; Brazil. *Astrothelium punctulatum* Malme
H8b Lichexanthone on thallus and ascomata or on thallus only. 9

H9a Lichexanthone on thallus only, absent from ascomata; ascomata solitary but dense; thallus gall-like; eastern palaeotropical. *Astrothelium grossoides* Aptroot & Lücking
H9b Lichexanthone on thallus and ascomata; thallus not gall-like. 10

H10a Ascomata pseudostromatic, immersed in prominent pseudostromata that are often partly covered by thallus; pantropical. *Astrothelium porosum* (Ach.) Aptroot & Lücking

H10b Ascomata solitary, erumpent, whitish. 11

H11a Ascospores 20–25 µm long; neotropical. *Astrothelium vulcanum* Aptroot
H11b Ascospores 14–17 µm long; Brazil. *Astrothelium infravulcanum* Aptroot

H12a Ascospores small (15–30 µm long). 13
H12b Ascospores (small to) medium-sized (25–70 µm long). 18

H13a Lichexanthone only on ascomata or ostiole. 14
H13b Lichexanthone on thallus and ascomata. 15

H14a Lichexanthone on ascomata only, absent from thallus; ascomata pseudostromatic, immersed in prominent pseudostromata covered by thallus; neotropical (Brazil and Bolivia). *Astrothelium stromatofluorescens* Aptroot & M. Cáceres
H14b Lichexanthone only on ostioles; pseudostromata sessile, brownish; Brazil. *Astrothelium gyalostiolatum* Aptroot

H15a Ascomata solitary to irregularly confluent, erumpent, covered by thallus; pantropical. *Astrothelium pulcherrimum* (Fée) Aptroot & Lücking
H15b Ascomata pseudostromatic, immersed in pseudostromata that are often partly covered by thallus. 16

H16a Pseudostromata well-delimited, with whitish cover, prominent to sessile; neotropical. *Astrothelium leucosessile* Lücking, M.P. Nelsen & Aptroot
H16b Pseudostromata not well-delimited, covered by thallus, erumpent to prominent; pantropical. 17

H17a Hamathecium clear, and pseudostromata UV+ yellow. *Astrothelium phlyctaena* (Fée) Aptroot & Lücking
H17b Hamathecium inspersed with oil droplets, and pseudostromata UV-. *Astrothelium luminothallinum* C. Zhang & S.H. Jiang

H18a Ascospores under 40 µm long. 19
H18b Ascospores over 45 µm long. 20

H19a Ascospores 25–40 × 8–13 µm; thallus and thalline cover of ascomata UV+ yellow; pantropical. *Astrothelium pupula* (Ach.) Aptroot & Lücking
H19b Ascospores 34–38 × 8.5–10.5 µm; only pseudocyphellae UV+ yellow; Brazil. *Astrothelium xanthopseudocyphellatum* Aptroot

H20a Ascospores 60–70 × 16–18 µm; El Salvador. *Astrothelium megochroleucum* Aptroot
H20b Ascospores 45–60 × 19.5–22.5 µm; Brazil. *Astrothelium miniannulare* Kalb & Aptroot

H21a Ascospores 3-septate. 22
H21b Ascospores 5–19-septate. 45

H22a Ascospores medium-sized (50–65 µm long); medulla (ascomata) with internal, redpigment; ascomata solitary to irregularly confluent, erumpent, covered by thallus; neotropical. *Astrothelium thelotremoides* (Nyl.) Aptroot & Lücking
H22b Ascospores small to medium-sized (15–50 µm long); pigment external or internal, yellow to red. 23

H23a Hamathecium inspersed. 24
H23b Hamathecium clear. 29

H24a Hamathecium with red, K+ green pigment; Sri Lanka. *Astrothelium isohypocrellinum* Aptroot & Weerakoon
H24b Hamathecium hyaline. 25

H25a Ascomata with external, yellow to orange pigment mostly developed around the ostiolar area; ascomata solitary to irregularly confluent, erumpent, covered by thallus except for black ostiolar area. 26
H25b Medulla (ascomata) with internal, red pigment; ascomata variable. 28

H26a Ascomata with yellow pigment; ascomata diffusely pseudostromatic, pseudostromata forming irregular lines; El Salvador. *Astrothelium neoinspersum* Aptroot
H26b Thallus and/or ascomata with orange pigment; ascomata solitary to irregularly confluent. 27

H27a Both thallus and ascomata with abundant orange pigment; pantropical. *Astrothelium inspersaeneum* Aptroot & M. Cáceres
H27b Only ascomata with sparse orange pigment; pantropical (Colombia and Papua New Guinea). *Astrothelium aenascens* Aptroot

H28a Ascospores 20–30 × 7–9 µm; Brazil. *Astrothelium rubrocrystallinum* Aptroot & M. Cáceres
H28b Ascospores 30–50 × 14–16 µm; neotropical. *Astrothelium buckii* (R.C. Harris) Aptroot & Lücking

ISSN 1868-4173

H29a Thallus and/or ascomata with external pigment(s). 30
H29b Medulla (ascomata) with internal pigment(s). 40

H30a Ascomata with external, red pigment(s); Mexico. *Astrothelium coccineum* Córd.-Cháv., Aptroot & M. Cáceres
H30b Ascomata with external, yellow to orange pigment(s). 31

H31a Thallus grey, barely lichenized; ascomata distinctly pseudostromatic, pseudostromata prominent to sessile, with orange cover strongly contrasting with thallus; Thailand. *Astrothelium aurantiacocinereum* Lücking, Naksuwankul & Lumbsch
H31b Thallus olive-green, distinctly lichenized; ascomata solitary or pseudostromatic, if pseudostromatic, then pseudostromata immersed to erumpent and in irregular, often reticulate lines. 32

H32a Ascospores very small (16–18 µm long); ascomata pseudostromatic, immersed, pseudostromata in irregular, often reticulate lines, covered with pigment and contrasting with olive-green thallus; Brazil. *Astrothelium flavum* Aptroot & M. Cáceres
H32b Ascospores small (20–35 µm long). 33

H33a Ascospores 20–25(–27) × 6–10 µm; ascomata solitary or pseudostromatic. 34
H33b Ascospores 25–35 × 10–12 µm; ascomata solitary to irregularly confluent, erumpent, covered by thallus. 38

H34a Ascomata solitary to irregularly confluent; pigment on thallus and ascomata. 35
H34b Ascomata pseudostromatic, pseudostromata in irregular lines, covered with pigment and contrasting with olive-green thallus. 37

H35a Thallus resembling bubbles of wax plus prothallus; Venezuela. *Astrothelium bullatothallinum* Aptroot & Sipman
H35b Thallus smooth. 36

H36a Pigment orange to yellow; pantropical. *Astrothelium aeneum* (Eschw.) Aptroot & Lücking
H36b Pigment ochraceous; Brazil. *Astrothelium ochraceum* Aptroot

H37a Thallus strongly bullate; Brazil. *Astrothelium flavostromatum* Aptroot & M. Cáceres
H37b Thallus smooth to uneven; neotropical. *Astrothelium kunzei* (Fée) Aptroot & Lücking

H38a Pigment orange, abundant; neotropical (Brazil & Bolivia). *Astrothelium megaeneum* Flakus & Aptroot
H38b Pigment yellow, sparse. 39

H39a Ostioles partly lateral and fused; pigment K+ purple; Bolivia. *Astrothelium pallidoflavum* Flakus & Aptroot
H39b Ostioles consistently apical; pigment K+ red; neotropical. *Astrothelium subcatervarium* (Malme) Aptroot & Lücking

H40a Ascospores medium-sized (30–50 × 10–16 µm). 41
H40b Ascospores small (15–30 × 5–9 µm). 43

H41a Medullary pigment red; neotropical. *Astrothelium cartilagineum* (Fée) Aptroot & Lücking
H41b Medullary pigment yellow. 42

H42a Thallus bullate; Brazil. *Astrothelium endochryseum* (Vain.) Aptroot & Lücking
H42b Thallus smooth; Brazil. *Astrothelium laevithallinum* Lücking, M.P. Nelsen & Marcelli

H43a Ascomata solitary to irregularly confluent, erumpent, covered by thallus; medullary pigment orange; Brazil. *Astrothelium aeneoides* Aptroot
H43b Ascomata pseudostromatic; pseudostromata blackish brown; medullary pigment (pale) yellow. 44

H44a Medullary pigment K+ red; pantropical. *Astrothelium degenerans* (Vain.) Aptroot & Lücking
H44b Medullary pigment K–. 16; neotropical. *Astrothelium feei* (C.F.W. Meissn.) Aptroot & Lücking

H45a Ascospores small to medium-sized (15–45 × 6–9 µm). 46
H45b Ascospores medium-sized to large (50–150 × 10–40 µm). 48

H46a Medulla (ascomata) with internal, red pigment; ascospores 9–13-septate; ascomata pseudostromatic, exposed, forming irregular, reticulate lines or groups; India. *Astrothelium nigrorufum* (Makhija & Patw.) Aptroot & Lücking
H46b Medulla (ascomata) with internal, yellow to orange pigment; ascospores 5–9-septate. 47

H47a Hamathecium inspersed; medullary pigment K+ red; ascomata pseudostromatic, pseudostromata erumpent to prominent, whitish; Papua New Guinea. *Astrothelium pseudodissimulum* Aptroot

ISSN 1868-4173

H47b Hamathecium clear; medullary pigment K+ purple; ascomata pseudostromatic, exposed, forming irregular, reticulate lines or groups; India. *Astrothelium dissimilum* (Makhija & Patw.) Aptroot & Lücking

H48a Ascospores medium-sized (50–90 µm long). 49
H48b Ascospores large (110–150 µm long). 50

H49a Ascomata with scattered, external, pink pigment, solitary to irregularly confluent, immersed in large thallus verrucae; hamathecium inspersed; ascospores 50–65 × 10–17 µm; Brazil. *Astrothelium decemseptatum* Aptroot & M. Cáceres
H49b Medulla (ascomata) with internal, yellow pigment; ascomata pseudostromatic, pseudostromata prominent, covered by thallus except black ostiolar areas; hamathecium clear; ascospores 55–90 × 13–28 µm; East Asia *Astrothelium luridum* (Zahlbr.) Aptroot & Lücking

H50a Ascomata with scattered, external, pink pigment, solitary, immersed; ascospores 17–22 µm broad, 13–19-septate; Caribbean. *Astrothelium calosporum* (Müll. Arg.) Aptroot & Lücking
H50b Ascomata with external and internal yellow pigment; ascospores 30–37µm broad, 7–11-septate; neotropical. *Astrothelium rimosum* Aptroot

Key I. *Astrothelium* species with ascospores transversely septate, ostiole apical, secondary substances absent

I1a Ascospores 3-septate. 2
I1b Ascospores (3–)5–15-septate. 28

I2a Ascospores small to medium-sized (15–45(–50) × 6–16 µm). 3
I2b Ascospores medium-sized to large ((45–)50–120 × 15–35 µm). 22

I3a Hamathecium inspersed. 4
I3b Hamathecium clear. 7

I4a Ascospores (25–)30–40 µm long; ascomata in irregular groups to pseudostromatic, pseudostromata erumpent, white, contrasting with olive-green thallus; pantropical. *Astrothelium perspersum* Aptroot & Ertz
I4b Ascospores 15–25(–30) µm long. 5

I5a Ascomata solitary. 6

I5b Ascomata irregularly grouped to pseudostromatic, immersed to erumpent, mostly exposed, brown-black; pantropical. *Astrothelium scoria* (Fée) Aptroot & Lücking

I6a Ascomata erumpent, with white cover; neotropical. *Astrothelium subscoria* Flakus & Aptroot
I6b Ascomata erumpent to prominent, not white-covered, thallus bullate. *Astrothelium jiangxiense* C. Zhang & S.H. Jiang

I7a Ascospores I+ violet. 8
I7b Ascospores I−. 9

I8a Ascomata solitary, immersed, covered by thallus except dark ostiolar area; pantropical. *Astrothelium infossum* (Nyl.) Aptroot & Lücking
I8b Ascomata distinctly pseudostromatic, pseudostromata erumpent, brown-black, with pale ostiolar areas; neotropical. *Astrothelium infuscatulum* (Müll. Arg.) Aptroot & Lücking

I9a Ascospores 15–28 × 7–10(–12) µm (*Astrothelium nitidiusculum* complex). 10
I9b Ascospores 26–46 × (7–)10–16 µm. 17

I10a Ascomata pseudostromatic; pseudostromata either prominent or contrasting with thallus in colour. 11
I10b Ascomata solitary to irregularly confluent or grouped but not prominent or contrasting with thallus. 15

I11a Pseudostromata prominent, partly covered by thallus and hence not strongly contrasting with thallus in colour; pantropical. *Astrothelium bicolor* (Taylor) Aptroot & Lücking
I11b Pseudostromata immersed to erumpent and more or less leveled with thallus, often forming irregular to reticulate lines or groups, contrasting with thallus in colour (either dark or whitish). 12

I12a Pseudostromata with whitish cover; neotropical. 13
I12b Pseudostromata exposed, brown-black. 14
I13a Pseudostromata with more ascomata; neotropical. *Astrothelium rufescens* (Müll. Arg.) Aptroot & Lücking
I13b Pseudostromata with few ascomata; China. *Astrothelium subrufescens* C. Zhang & S.H. Jiang

I14a Pseudostromata forming distinct, compact groups laterally covered by thallus; neotropical. *Astrothelium subendochryseum* Lücking, M.P. Nelsen & Marcelli
I14b Pseudostromata diffuse, exposed; neotropical. *Astrothelium nigratum (*Müll. Arg.) Aptroot & Lücking

I15a Ascomata forming irregular groups, with rather broad, brown-black ostiolar area; eastern palaeotropical. *Astrothelium meiophorum* (Nyl.) Aptroot & Lücking
I15b Ostiolar areas not broadly. 16

I16a Ascomata covered by olive-green thallus; neotropical. *Astrothelium ceratinum* (Fée) Aptroot & Lücking
I16b Ascomata with broadly white area surrounding the ostiole or entirely white; pantropical. *Astrothelium nitidiusculum* (Nyl.) Aptroot & Lücking

I17a Ascospores 36–46 × 14–16 µm; ascomata solitary, erumpent, covered by thallus and with pale ring surrounding the dark ostiolar spot; pantropical, extending into temperate regions. *Astrothelium floridanum* Zahlbr. ex M. Choisy
I17b Ascospores 26–39 × 7–14 µm; ascomata variable. 18

I18a Ascomata pseudostromatic, pseudostromata exposed, immersed and leveled with thallus, forming irregular to reticulate lines or groups. 19
I18b Ascomata solitary, covered by thallus. 21

I19a Thallus consisting of globose verrucae with a thick hyaline cortex; Papua New Guinea. *Astrothelium diaphanocorticatum* Aptroot & Sipman
I19b Thallus smooth to verrucose. 20

I20a Ascospores 36–39 µm long; neotropical. *Astrothelium scoriothelium* Aptroot & Lücking
I20b Ascospores 29–33 µm long; Brazil. *Astrothelium disjunctum* Aptroot & M. Cáceres

H21a Thallus and ascomata finely verrucose; ascomata immersed to erumpent, numerous and dense; neotropical. *Astrothelium papulosum* (Nyl.) Aptroot & Lücking
H21b Thallus and ascomata smooth to uneven; ascomata erumpent to prominent, scattered; neotropical. *Astrothelium solitarium* Aptroot & M. Cáceres

I22a Hamathecium inspersed. 23
I22b Hamathecium clear. 25

I23a Ascospores 45–62 × 15–22 µm; thallus uneven-bullate; ascomata solitary, erumpent, covered by thallus, with pale ring surrounding the dark ostiolar spot; Bolivia. *Astrothelium inspersotuberculosum* Flakus & Aptroot

I23b Ascospores 85–120 × 22–35 µm; thallus and ascomata variable. 24

I24a Ascospores 85–95 × 22–25 µm; thallus uneven-verrucose; ascomata solitary, prominent, exposed, with gently sloping sides; Vietnam. *Astrothelium clypeatum* Aptroot & Gueidan
I24b Ascospores 100–120 × 30–35 µm; thallus uneven-verrucose; ascomata confluent, erumpent to prominent, exposed, with gently to steeply sloping sides; Guyana. *Astrothelium megatropicum* Aptroot

I25a Ascomata prominent, completely exposed; Colombia. *Astrothelium phaeothelium* (Nyl.) Aptroot & Lücking
I25b Ascomata immersed to erumpent, covered by thallus, with pale ring surrounding the dark ostiolar spot. 26

I26a Thallus verrucose-papillose; ascomata solitary to irregularly confluent, prominent to sessile; Panama. *Astrothelium pseudannulare* Aptroot & Etayo
I26b Thallus uneven to shallowly bullate. 27

I27a Ascospores 48–65 × 17–20 µm; neotropical. *Astrothelium tuberculosum* (Vain.) Aptroot & Lücking
I27b Ascospores 62–80 × 20–25 µm; neotropical. *Astrothelium annulare* (Fée) Aptroot & Lücking

I28a Ascospores medium-sized (29–59(–80) × 8–17 µm). 29
I28b Ascospores large (85–220 × (8–)20–45 µm). 34

I29a Hamathecium inspersed. 30
I29b Hamathecium clear. 31

I30a Ascospores 50–59 µm long; thallus verrucose-bullate; Papua New Guinea. *Astrothelium galligenum* (Aptroot) Aptroot & Lücking
I30b Ascospores 40–49 µm long; thallus smooth to uneven; Sri Lanka. *Astrothelium nitidulum* Weerakoon & Aptroot

I31a Ascomata at least mostly solitary. 32
I31b Ascomata in irregular groups to pseudostromatic, groups or pseudostromata erumpent to prominent. 33

I32a Ascomata solitary to irregularly confluent, immersed to erumpent, with erumpent part exposed, blackish; Malaysia. *Astrothelium trypethelizans* (Nyl.) Aptroot & Lücking

ISSN 1868-4173

I32b Ascomata solitary, immersed, not exposed; Brazil. *Astrothelium quintannulare* Aptroot

I33a Pseudostromata whitish; eastern palaeotropical. *Astrothelium ubianense* (Vain.) Aptroot & Lücking
I33b Pseudostromata brownish; Brazil. *Astrothelium pseudoplatystomum* (Makhija & Patw.) Aptroot & Lücking

I34a Hamathecium inspersed. 35
I34b Hamathecium. 40

I35a Ascospores 9–15-septate, occasionally with a longitudinal septum in one or several cells. 36
I35b Ascospores 5–9-septate, lacking longitudinal septa. 37

I36a Ascospores 120–195 µm long; India. *Astrothelium macrosporum* (Makhija & Patw.) Aptroot & Lücking
I36b Ascospores 75–100 µm long; Malaysia. *Astrothelium tanianum* Aptroot & Sipman

I37a Ascospores 180–220 × 40–45 µm, 5–9-septate; ascomata grouped in large thallus verrucae; Angola. *Astrothelium pustulatum* (Vain.) Aptroot & Lücking
I37b Ascospores 100–170 × 29–40 µm, 5-septate; ascomata variable. 38

I38a Ascospores curved; ascomata grouped; Brazil. *Astrothelium curvisporum* Aptroot & M. Cáceres
I38b Ascospores straight; ascomata solitary to irregularly confluent. 39

I39a Ascomata solitary, prominent to sessile, covered by thallus except dark ostiolar area; neotropical (Brazil and Guyana). *Astrothelium sipmanii* Aptroot
I39b Ascomata solitary to irregularly confluent, erumpent, upper part exposed; Colombia. *Astrothelium pseudomegalophthalmum* Aptroot

I40a Ascospores 140–190 µm long, 11–19-septate; ascomata solitary to irregularly confluent. 41
I40b Ascospores 85–120 µm long, 3–17-septate; ascomata irregularly grouped to pseudostromatic. 42

I41a Thallus verrucose-bullate; ascomata erumpent to prominent, covered by thallus except broad, blackened ostiolar area; Uruguay. *Astrothelium basilicum* (Kremp.) Aptroot & Lücking
I41b Thallus smooth; ascomata immersed, completely covered by thallus; Argentina. *Astrothelium spectabile* (Aptroot & L.I. Ferraro) Aptroot & Lücking

I42a Ascospores 20–33 µm broad, 3–7-septate. 43
I42b Ascospores 8–16 µm broad, 9–17-septate; neotropical. *Astrothelium olivaceofuscum* (Zenker) Aptroot & Lücking

I43a Thallus smooth to uneven; ascomata covered by thallus; Brazil. *Astrothelium trematum* Kalb & Aptroot
I43b Thallus verrucose-bullate; ascomata exposed, brown-black. 44

I44a Thallus verrucose-bullate; Brazil. *Astrothelium megalophthalmum* (Müll. Arg.) Aptroot & Lücking
I44b Thallus squamulose; Brazil. *Astrothelium squamosum* Aptroot

Key J. *Astrothelium* species with ascospores transversely septate, ostiole lateral, secondary substances present on thallus and/or ascoma

J1a Lichexanthone present; thallus and/or ascomata UV+ yellow; pigment(s) present or absent. 2
J1b Lichexanthone absent; pigment(s) always present, thallus and ascomata usually K+ red to purple and UV+ orange or red. 38

J2a Pigments present on/in thallus and/or ascomata; lichexanthone on thallus only. 3
J2b Pigments absent; lichexanthone on thallus and/or ascomata. 10

J3a Ascospores 9–11-septate, 50–75 × 12–22 µm; thallus with external, orange pigment (absent on ascomata); ascomata solitary to irregularly confluent, erumpent, covered by thallus except dark ostiolar area; Venezuela. *Astrothelium dicoloratum* Aptroot
J3b Ascospores 3-septate, 21–55 × 7–23 µm; ascomata with external, yellow-orange pigment (absent on thallus). 4

J4a Hamathecium inspersed; ascospores 20–25 × 9–11 µm; groups of fused ascomata dispersed to irregularly confluent; Singapore. *Astrothelium inspersogalbineum* Aptroot & Weerakoon
J4b Hamathecium clear; ascospores variable in size. 5

J5a Ascospores 21–28 × 7–11 µm. 6
J5b Ascospores 28–55 × 10–23 µm. 7

J6a Ascomata pseudostromatic; pseudostromata prominent, containing one or several groups of fused ascomata, covered with orange pigment; pantropical. *Astrothelium macrocarpum* (Fée) Aptroot & Lücking
J6b Ascomata solitary to irregularly confluent, erumpent, containing a single group of fused ascomata; Thailand. *Astrothelium flavocoronatum* Luangsuph., Aptroot & Sangvichien

J7a Ascospores 37–55 × 15–23 µm; ascomata pseudostromatic, pseudostromata erumpent, containing several groups of fused ascomata, covered with orange pigment; neotropical. *Astrothelium ochrothelium* (Nyl.) Müll. Arg.
J7b Ascospores 28–35 × 10–15 µm. 8

J8a Thallus strongly folded-bullate, whitish; groups of fused ascomata dispersed, immersed, with yellow-orange pigment around ostiolar area; pantropical. *Astrothelium versicolor* Müll. Arg.
J8b Thallus smooth to uneven, light olive-brown to olive-green; ascomata pseudostromatic. 9

J9a Pseudostromata immersed to erumpent, containing several groups of fused ascomata, forming irregular to reticulate lines covered with yellow-orange pigment; Brazil. *Astrothelium aureomaculatum* (Vain.) Aptroot & Lücking
J9b Pseudostromata erumpent to prominent, each containing one group of fused ascomata, covered by orange pigment; New Caledonia. *Astrothelium grossum* Müll. Arg.

J10a Ascospores 3-septate, rarely 3(–5)-septate. 11
J10b Ascospores (3–)5–11-septate. 27

J11a Ascospores small to medium-sized (20–52 × 6–17 µm). 12
J11b Ascospores medium-sized to large (50–120 × 17–40 µm). 23

J12a Ascospores 30–52 × 10–17 µm. 13
J12b Ascospores 20–30 × 6–11 µm. 16

J13a Hamathecium inspersed, inspersion dirty yellow-brown; lichexanthone on thallus and ascomata or ascomata only; ascomata pseudostromatic, pseudostromata prominent, containing one or several groups of fused ascomata. 14
J13b Hamathecium clear; lichexanthone on thallus and ascomata; ascomata crowded or indistinctly pseudostromatic, pseudostromata immersed to erumpent, containing one or several groups of fused ascomata. 15

J14a Lichexanthone on ascomata only; Brazil. *Astrothelium sordithecium* Lücking, M.P. Nelsen & Marcelli
J14b Lichexanthone on thallus and ascomata; Brazil. *Astrothelium xanthosordithecium* Aptroot

J15a Ascomata solitary or in dispersed, diffuse pseudostromata, conical with flattened top, whitish; neotropical. *Astrothelium leucothelium* Nyl.
J15b Ascomata crowded, hemispherical to wart-shaped, covered by thallus; Malaysia. *Astrothelium chrysostomum* (Kremp.) Aptroot & Lücking

J16a Lichexanthone on ascomata or only on ostiole or only on thallus. 17
J16b Lichexanthone on thallus and ascomata; hamathecium inspersed or clear; ascospores 20–26 µm long. 21

J17a Lichexanthone only on ostiole; hamathecium inspersed; Brazil. *Astrothelium eustominspersum* Aptroot & Oliveira-Junior
J17b Lichexanthone on ascomata only; hamathecium clear or inspersed. 18
J17c Lichexanthone on the thallus only; hamathecium inspersed. *Astrothelium subeustominspersum* C. Zhang & S.H. Jiang

J18a Hamathecium inspersed; Brazil. *Astrothelium studerae* Aptroot & M. Cáceres
J18b Hamathecium clear. 19

J19a Hamathecium inspersed; ascomata diffusely pseudostromatic, erumpent, with whitish cover; Thailand. *Astrothelium neovariolosum* Luangsuph., Aptroot & Sangvichien
J19b Hamathecium clear. 20

J20a Groups of fused ascomata dispersed, immersed to erumpent; Brazil. *Astrothelium laevigatum* Müll. Arg.
J20b Ascomata pseudostromatic, pseudostromata prominent, often whitish, containing one or several groups of fused ascomata; pantropical. *Astrothelium variolosum* (Ach.) Müll. Arg.

J21a Ascomata diffusely pseudostromatic, pseudostromata erumpent, covered by thallus, containing one or several groups of fused ascomata; ascospores 20–25 µm; Brazil. *Astrothelium subinterjectum* Lücking, M.P. Nelsen & Jungbluth
J21b Ascomata distinctly pseudostromatic, pseudostromata prominent, with white or grey cover contrasting with the olive-green thallus, containing one or several groups of fused ascomata. 22

ISSN 1868-4173

J22a Thallus with pseudocyphellae; pseudostromata grey; Brazil. *Astrothelium pseudocyphellatum* R.C. Harris
J22b Thallus lacking pseudocyphellae; pseudostromata whitish; pantropical. *Astrothelium interjectum* R.C. Harris

J23a Ascospores 100–130 × 35–45 µm; groups of fused ascomata dispersed to aggregate, immersed-erumpent, covered by thallus except for whitish ostiolar area; Venezuela. *Astrothelium ultralucens* Aptroot
J23b Ascospores 50–75 × 14–26 µm. 24

J24a Lichexanthone on thallus (and sometimes also on ascomata). 25
J24a Lichexanthone not on thallus, only on ascomata. 26

J25a Lichexanthone on thallus, not on ascomata; neotropical (Brazil and Venezuela). *Astrothelium leucoconicum* Nyl.
J25b Lichexanthone on thallus and ascomata; Bolivia. *Astrothelium nigrocacuminum* Flakus, Kukwa & Aptroot

J26a Ascomata distinctly pseudostromatic, pseudostromata erumpent, with white cover contrasting with the olive-green thallus, containing one or several groups of fused ascomata; ascospores 60–70 × 17–20 µm; Brazil. *Astrothelium sinuosum* Aptroot & Gumboski
J26b Groups of fused ascomata dispersed, immersed, with broad, dark ostiolar area; ascospores 50–60 × 14–17 µm; Brazil. *Astrothelium obtectum* Lücking, M.P. Nelsen & Benatti

J27a Ascospores small (20–30 × 6–11 µm), 3–5-septate. 28
J27b Ascospores medium-sized to large (30–110 × 11–25 µm), 5–11-septate. 30

J28a Lichexanthone on ostioles only; ascomata distinctly pseudostromatic, pseudostromata erumpent, with white cover contrasting with the olive-green thallus, containing one or several groups of fused ascomata; pantropical. *Astrothelium eustomum* (Mont.) Müll. Arg.
J28b Lichexanthone on thallus and ascomata; ascomata variable. 29

J29a Ascomata distinctly pseudostromatic, pseudostromata erumpent, with white cover contrasting with the olive-green thallus; Thailand. *Astrothelium neglectum* Luangsuph., Aptroot & Sangvichien
J29b Ascomata indistinctly pseudostromatic, pseudostromata erumpent to prominent, covered by thallus; pantropical. *Astrothelium sexloculatum* Aptroot

J30a Ascospores 30–55 µm long; ascomata with white cover contrasting with the olive green thallus, erumpent. 31
J30b Ascospores 65–110 µm long; ascomata variable. 34

J31a Hamathecium inspersed; ascomata dispersed to irregularly confluent; Thailand. *Astrothelium siamense* Luangsuph., Aptroot & Sangvichien
J31b Hamathecium clear; ascomata distinctly pseudostromatic, containing several groups of fused ascomata. 32

J32a Lichexanthone on thallus and ascomata; neotropical (Guyana and Venezuela). *Astrothelium septemseptatum* Aptroot
J32b Lichexanthone on ascomata or ostiole only. 33

J33a Lichexanthone on ascomata; Brazil. *Astrothelium octoseptatum* Aptroot & M. Cáceres
J33b Lichexanthone on ostiole only; French Guiana. *Astrothelium macroeustomum* Aptroot & Sipman

J34a Hamathecium inspersed; lichexanthone on thallus and ascomata; ascomata distinctly pseudostromatic, pseudostromata erumpent, with white cover contrasting with the olive-green thallus, each containing a single group of fused ascomata; Cuba. *Astrothelium diplocarpoides* Müll. Arg.
J34b Hamathecium clear; lichexanthone on ascomata only; ascomata variable. 35

J35a Ascospores 80–110 × 17–25 µm, 5–7-septate; ascomata diffusely pseudostromatic, pseudostromata erumpent, covered by thallus, each containing a single group of fused ascomata; Brazil. *Astrothelium macrostomoides* Lücking, M.P. Nelsen & Benatti
J35b Ascospores when transversely septate 65–85 × 14–18 µm, 5–11-septate, submuriform when longer; ascomata variable. 36

J36a Ascospores 5–7-septate; ascomata diffusely pseudostromatic, pseudostromata erumpent to prominent, covered by thallus, uneven-verrucose, each containing a single group of fused ascomata; French Guiana. *Astrothelium macrostomum* Aptroot
J36b Ascospores 9–11-septate; ascomata distinctly pseudostromatic, pseudostromata immersed, with white cover contrasting with the olive-green thallus, containing one to several groups of fused ascomata. 37

J37a Ascospores 65–70 × 15–17 µm, transversely septate only; Brazil. *Astrothelium eumultiseptatum* Aptroot & M. Cáceres

J38b Ascospores 90–110 × 22–28 µm, in part with longitudinal septa and becoming submuriform; neotropical. *Astrothelium diplocarpum* Nyl.

J38a Ascospores 5–9-septate. 39
J38b Ascospores 3-septate. 41

J39a Ascospores 5-septate; ascomata diffusely pseudostromatic, pseudostromata with external pink pigment; Brazil. *Astrothelium bivelum* Aptroot & M. Cáceres
J39b Ascospores 5–9-septate. 40

J40a Ascospores 7–9-septate; only ostiole yellow pruinose; Brazil. *Astrothelium septoconicum* Aptroot
J40b Ascospores 6–7-septate; pseudostromata with internal yellow pigment; Brazil. *Astrothelium introflavidum* Aptroot & M. Cáceres

J41a Ascospores small (20–30 × 6–12 µm). 42
J41b Ascospores small to medium-sized (30–65 × 10–25 µm). 47

J42a Thallus and ascomata with orange pigment. 43
J42b Pigment on ascomata only, yellow to orange. 44

J43a Pseudostromata immersed to erumpent, whitish; Indonesia. *Astrothelium pseudoferrugineum* Aptroot
J43b Pseudostromata erumpent to prominent, covered by thallus; Cuba. *Astrothelium ferrugineum* (Müll. Arg.) Aptroot & Lücking

J44a Hamathecium inspersed; Sri Lanka. *Astrothelium inspersoconicum* Aptroot & Weerakoon
J44b Hamathecium clear. 45

J45a Pigment yellow, sparse; ostioles partly apical; ascomata solitary to irregularly confluent and fused, erumpent, covered by thallus; Bolivia. *Astrothelium pallidoflavum* Flakus & Aptroot
J45b Pigment orange, distinct; ostioles consistently lateral and fused; ascomata pseudostromatic, erumpent to prominent, with orange cover, containing one to many of fused ascomata. 46

J46a Ascomata with one or few fused ascomata; pantropical. *Astrothelium cinnamomeum* (Eschw.) Müll. Arg.
J46b Ascomata pseudostromatic, with many groups of fused ascomata; Brazil. *Astrothelium stromatocinnamomeum* Aptroot

J47a Thallus yellow; ascospores 30–35 × 10–12 µm; Brazil. *Astrothelium croceum* Malme
J47b Thallus not cracked; ascospores (30–)35–65 × (10–)12–25 µm. 48

J48a Ascospores 55–65 × 15–25 µm; pantropical. *Astrothelium ocellatum* Malme
J48b Ascospores 30–45 × 10–17 µm; pantropical. *Astrothelium scorioides* Nyl.

Key K. *Astrothelium* species with ascospores transversely septate, ostiole lateral, secondary substances absent

K1a Ascospores 3-septate. 2
K1b Ascospores (3–)5–23-septate. 21

K2a Ascospores medium-sized (55–80 × 15–25 µm); thallus distinctly verrucose papillose. 3
K2b Ascospores small to medium-sized ((12–)20–45 × (4–)7–15 µm); thallus variable. 4

K3a Ascospores medium-sized (55–65 × 15–19 µm); thallus distinctly verrucose-papillose; ascomata immersed; Brazil. *Astrothelium simplex* Aptroot & S.M.A. Martins
K3b Ascospores 80–88 × 32–36 µm; ascomata prominent to sessile; Panama. *Astrothelium pseudannulare* Aptroot & Etayo

K4a Hamathecium inspersed. 5
K4b Hamathecium clear. 7

K5a Thallus covering the pseudostromata; Sri Lanka. *Astrothelium conjugatum* Weerakoon & Aptroot
K5b Thallus not covering the pseudostromata. 6

K6a Ascospores 20–25 µm long; ascomata diffusely pseudostromatic, pseudostromata prominent, laterally covered by thallus, with one to several groups of fused ascomata forming broad, flat, dark ostiolar areas often fused in lobate pattern; eastern palaeotropical. *Astrothelium straminicolor* (Nyl.) Aptroot & Lücking
K6b Ascospores 25–30 µm long; ascomata diffusely pseudostromatic, pseudostromata prominent, covered by thallus, with one to several groups of fused ascomata with dark, papilliform, always separate ostiolar areas; pantropical. *Astrothelium pyrenastrosulphureum* Aptroot & Lücking

K7aAscospores (12–)20–27 × (4–)7–10 µm. 8
K7b Ascospores 28–45 × 10–15 µm. 17

ISSN 1868-4173

K8a Ascospores 12–15 × 4–5 µm; ascomata diffusely pseudostromatic, pseudostromata prominent, laterally covered by thallus, with one to several groups of fused ascomata forming broad, flat, dark ostiolar areas often fused in lobate pattern; Cuba. *Astrothelium acrophaeum* Müll. Arg.
K8b Ascospores 19–27 × 7–10 µm; thallus and ascomata variable (*Astrothelium crassum* complex). 9

K9a Ostioles separate; ascomata aggregate in irregular groups or lines, immersed; neotropical. *Astrothelium scorizum* (Müll. Arg.) Aptroot & Lücking
K9b Ostioles fused; fused ascomata dispersed to aggregate. 10

K10a Thallus mottled olive-whitish, with irregular whitish areas or lines; ascomata usually developed on the whitish areas (but not covered by them). 11
K10b Thallus uniformly olive-brown to olive-green, without whitish areas except when covering the ascomata. 13

K11a Ascomata dispersed, conical, partially or fully covered by thallus; Malaysia. *Astrothelium peranceps* (Kremp.) Aptroot & Lücking
K11b Ascomata dispersed to irregularly confluent, completely exposed. 12

K12a Ascomata black, developed on distinct, white, irregular to reticulate lines; Brazil. *Astrothelium nigricans* Malme
K12b Ascomata dark brown, emerging from thallus; neotropical. *Astrothelium nigrum* Aptroot & M. Cáceres

K13a Ascomata aggregate in irregular groups bordered by a thin whitish line; thallus rather thick, uneven to irregularly folded; eastern palaeotropical. *Astrothelium subfuscum* Kremp.
K13b Ascomata dispersed to irregularly confluent, exposed and brown or with whitish or (partial) thallus cover; thallus thin to thick but not folded. 14

K14a Ascomata exposed and brown-black, conical; Brazil. *Astrothelium obscurum* Müll. Arg.
K14b Ascomata at least with lateral thallus cover or with whitish rim or cover, lens-shaped to hemispherical. 15

K15a Ascomata prominent, with basal thallus cover and upper part exposed; individual ascomata clearly discernible; Australia. *Astrothelium oligocarpum* (Müll. Arg.) Aptroot & Lücking
K15b Ascomata erumpent to prominent, whitish or whitish rim; individual ascomata only visible as immersed chambers. 16

K16a Ascomata with complete whitish cover including ostiolar area; thallus thin; Thailand. *Astrothelium subdissocians* (Nyl. ex Vain.) Aptroot & Lücking
K16b Ascomata with whitish rim bordering a wide, dark ostiolar area; thallus thickened; pantropical. *Astrothelium crassum* (Fée) Aptroot
K16c Ascomata irregularly grouped to pseudostromatic, pseudostromata usually white-covered and the thallus thin. *Astrothelium pseudocrassum* C. Zhang & S.H. Jiang

K17a Thallus strongly verrucose-bullate; groups of fused ascomata dispersed, immersed, visible only by their dark, narrow ostiolar area; Brazil. *Astrothelium globosum* Aptroot & M. Cáceres
K17b Thallus smooth to uneven; ascomata distinct, diffusely to distinctly pseudostromatic, pseudostromata covered with thallus. 18

K18a Ascomata distinctly pseudostromatic, pseudostromata strongly prominent to sessile, uneven-verrucose, covered with thallus, with whitish ring around dark ostiolar zone, containing one or several groups of fused ascomata; pantropical. *Astrothelium fallax* Müll. Arg.
K18b Ascomata diffusely pseudostromatic, pseudostromata erumpent to prominent, smooth to uneven, covered with thallus, containing several to many groups of fused ascomata. 19

K19a Ascospores 35–45 × 14–16 µm; Brazil. *Astrothelium marcidum* (Fée) Aptroot & Lücking
K19b Ascospores 28–33 × 8–15 µm. 20

K20a Pseudostromata with distinctly separate, whitish ostiolar areas; ascospores 12–15 µm broad; Costa Rica. *Astrothelium intermedium* Aptroot & Lücking
K20b Pseudostromata with upper part entirely whitish; ascospores 8–10 µm broad; Brazil. *Astrothelium pleiostomum* Redinger

K21a Ascospores small and slender, 6–9-septate, 35–54 × 7–10 µm, curved; India. *Astrothelium himalayense* (Upreti &Aptroot) Aptroot & Schumm
K21b Ascospores medium-sized (45–80 × 12–28 µm). 22
K21c Ascospores large (80–130 × 17–30 µm). 29

K22a Hamathecium inspersed. 23
K22b Hamathecium clear. 24

K23a Ascospores 45–55 × 13–16 µm, 7–9-septate; ascomata indistinctly pseudostromatic, pseudostromata erumpent to prominent, brownish; Venezuela. *Astrothelium trypethelioides* Aptroot

K23b Ascospores 50–75 × 14–22 µm, 5-septate; ascomata diffusely pseudostromatic, pseudostromata erumpent, covered by thallus, containing one to several groups of fused ascomata; pantropical. *Astrothelium subclandestinum* Leight.

K23c Ascomata in distinct, white pseudostromata; Vietnam. *Astrothelium decemloculare* Aptroot, Weerakoon & G. Vo

K24a Ascospores 4–5-septate; groups of fused ascomata dispersed, erumpent, with whitish ring around dark ostiolar zone. 25

K24b Ascospores 7–19-septate; ascomata variable. 26

K25a Ascospores predominantly 4-septate, 45–55 × 15–18 µm; Brazil. *Astrothelium quatuorseptatum* Aptroot & M. Cáceres

K25b Ascospores 5-septate, 50–75 × 14–22 µm; Brazil. *Astrothelium supraclandestinum* Aptroot & M. Cáceres

K26a Ascospores 7-septate, 14–28 µm broad; ascomata pseudostromatic, pseudostromata immersed-erumpent, with whitish cover; neotropical (Brazil and Guyana). *Astrothelium zebrinum* Aptroot

K26b Ascospores 9–19-septate, 12–17 µm broad. 27

K27a Ascospores 45–55 µm long, consistently 9-septate; ascomata pseudostromatic, pseudostromata erumpent, with white cover contrasting with olive-green thallus, with several to many groups of fused ascomata; Brazil. *Astrothelium novemseptatum* Aptroot & M. Cáceres

K27b Ascospores 60–80 µm long, 9–19-septate. 28

K28a Ascospores 69–80 µm long, 11–15-septate; ascomata deeply immersed in groups; Brazil. *Astrothelium valsoides* Aptroot & M. Cáceres

K28b Ascospores 60–80 µm long, 9–19-septate; groups of fused ascomata dispersed, erumpent, brown-black with whitish ring around base; eastern palaeotropical. *Astrothelium heterophorum* Nyl.

K29a Ascospores I+ violet, occasionally with a longitudinal septum in one or several cells; thallus bullate-folded; groups of ascomata dispersed to irregularly confluent, immersed to erumpent, exposed and brown-black; Bolivia. *Astrothelium neodiplocarpum* Flakus, Kukwa & Aptroot

K29b Ascospores I−, lacking longitudinal septa; groups of fused ascomata dispersed (or ostioles separate), mostly covered by thallus. 30

K30a Hamathecium inspersed; groups of fused ascomata prominent, with broad white ostiolar area; ascospores 9–11-septate, 80–100 µm long; Thailand. *Astrothelium macrostiolatum* Luangsuph., Aptroot & Sangvichien

K30b Hamathecium clear; ascomata variable; ascospore septa variable, ascospores (90–)100–130 µm long. 31

K31a Ascospores 5–7(–9)-septate. 32
K31b Ascospores 9–23-septate. 33

K32a Ascospore median septum not thickened; neotropical. *Astrothelium robustum* Müll. Arg.
K32b Ascospore median septum thickened; Brazil. *Astrothelium fernandae* Aptroot

K33b Ascospores 9–11-septate, median septum thickened; ostioles fused; Brazil. *Astrothelium medioincrassatum* Aptroot & M. Cáceres
K33c Ascospores 11–23-septate. 34

K34a Ascospores 11–15-septate; groups of fused ascomata erumpent; Brazil. *Astrothelium robustosporum* Aptroot & M. Cáceres
K34b Ascospores 19–23-septate; groups of fused ascomata strongly prominent to sessile, with protruding, irregular ostioles; neotropical. *Astrothelium gigasporum* R.C. Harris

Key L. *Astrothelium* species with ascospores muriform, ostiole apical, secondary substances present on thallus and/or ascoma

L1a Lichexanthone present; thallus and/or ascomata UV+ yellow; pigment(s) present or absent. 2
L1b Lichexanthone absent; pigment(s) always present, thallus and ascomata usually K+ red to purple and UV+ orange or red. 14

L2a Pigments present on/in thallus and/or ascomata; ascospores large, 70–160 × 15–32 µm. 3
L2b Pigments absent; ascospores medium-sized to large, 40–160 × 15–40 µm. 6

L3c Hamathecium inspersed; ascomata mostly solitary; lichexanthone only on thallus. Vietnam. *Astrothelium curcumarum* Aptroot, Weerakoon & G. Vo
L3b Hamathecium clear. 4

L4a Thallus and ascomata with external, yellow-orange, K+ purple pigment(s); lichexanthone on thallus only; ascomata distinctly pseudostromatic, pseudostromata erumpent to prominent, covered with orange pigment, sharply delimited and contrasting with thallus; Brazil. *Astrothelium flavoduplex* Aptroot & M. Cáceres

L4b Ascomata or medulla with internal, red, K+ yellow-green pigment(s); lichexanthone on thallus and ascomata. 5

L5a Ascospores I+ violet; ascomata solitary to irregularly confluent, erumpent to prominent, whitish around ostiole(s); Brazil. *Astrothelium sanguineoxanthum* Aptroot
L5b Ascospores I−; ascomata distinctly pseudostromatic, pseudostromata erumpent to prominent, whitish, sharply delimited and contrasting with thallus; Bolivia. *Astrothelium elixii* Flakus & Aptroot

L6a Hamathecium inspersed. 7
L6b Hamathecium clear. 10

L7a Ascospores (2–)4(–6) per ascus, 40–80 µm long; on rock; Brazil. *Astrothelium stramineum* (Malme) Aptroot & Lücking
L7b Ascospores 8 per ascus. 8

L8a Ascospores 65–71 µm long; Brazil. *Astrothelium rhinothallinum* Kalb & Aptroot
L8b Ascospores 70–220 µm long. 9

L9a Ascospores 70–120 × 18–24 µm; eastern palaeotropical. *Astrothelium meristosporoides* (P.M. McCarthy & Vongshew.) Aptroot & Lücking
L9b Ascospores 120–220 × 25–40 µm; eastern palaeotropical. *Astrothelium meristosporum* (Mont. & Bosch) Aptroot & Lücking

L10a Ascospores 70–115 × 15–24 µm. 11
L10b Ascospores 110–170 × 20–35 µm. 13

L11a Lichexanthone on pseudostromata only; ascomata covered by thallus except for dark ostiolar area; neotropical (Brazil and Guyana). *Astrothelium corallinum* Aptroot
L11b lichexanthone on thallus, sometimes also on ascomata. 12

L12a Ascospores 70–90 × 18–20 µm, 8 per ascus; lichexanthone on thallus only; ascomata indistinctly pseudostromatic, pseudostromata erumpent, whitish, not sharply delimited; Guyana. *Astrothelium lucidothallinum* Aptroot
L12b Ascospores 80–115 × 15–24 µm, 4 per ascus; lichexanthone on thallus and ascomata; ascomata distinctly pseudostromatic, pseudostromata erumpent to prominent, brown-black, sharply delimited; Brazil. *Astrothelium ochroleucoides* Aptroot & M. Cáceres

L13a Ascomata pseudostromatic; pseudostromata erumpent, whitish; Bolivia. *Astrothelium variabile* Flakus & Aptroot
L13b Ascomata pseudostromatic; pseudostromata erumpent, brown-black; Brazil. *Astrothelium xanthosuperbum* Aptroot & M. Cáceres

L14a Ascospores medium-sized, 45–80 µm long. 15
L14b Ascospores large over 80 µm long. 21

L15a Ascospores 40–55 µm long. 16
L15b Ascospores 60–80 µm long. 18

L16a Ascomata with internal, yellow pigment; ascospores about 3–6 times as long as broad. 17
L16b Thallus and ascomata with external, orange pigment and medulla (ascomata) additionally with internal, purple pigment; ascospores about twice as long as broad; Ecuador. *Astrothelium condoricum* Aptroot

L17a Acospores about 4–6 times as long as broad; Brazil. *Astrothelium lineatum* Aptroot & M. Cáceres
L17b Ascospores about 3–4 times as long as broad; Brazil. *Astrothelium duplicatum* Aptroot & M. Cáceres

L18a Ascomata with internal red pigment crystals; ostiolar area very broad and flat, black; Sierra Leone. *Astrothelium sierraleonense* (C.W. Dodge) Aptroot & Lücking
L18b Pigment external; ostiolar area narrow, whitish. 19

L19a Hamathecium inspersed; pigment on ascomata only; ascospores 20–30 µm broad; ascomata pseudostromatic, pseudostromata prominent, covered with orange pigment, sharply delimited from thallus; neotropical. *Astrothelium auratum* (R.C. Harris) Aptroot & Lücking
L19b Hamathecium clear; pigment on thallus and ascomata; ascospores 12–25 µm broad; ascomata variable. 20

L20a Ascomata pseudostromatic; pseudostromata erumpent, covered with orange pigment, sharply delimited; Brazil. *Astrothelium graphicum* Aptroot & S.M.A. Martins
L20b Ascomata pseudostromatic; pseudostromata immersed, whitish, not sharply delimited; neotropical. *Astrothelium flavomaculatum* Aptroot

L21a Ascomata with internal, red, K+ yellow-green pigment; ascospores I + violet; ascomata pseudostromatic, pseudostromata erumpent to prominent, brown-black,

ISSN 1868-4173

sharply delimited; Brazil. *Astrothelium sanguinarium* (Malme) Aptroot & Lücking
L21b Thallus and/or ascomata with external or internal, yellow to orange, K+ purple pigment; ascospores I−; ascomata variable. 22

L22a Pigment in medulla of pseudostromata only. 23
L22b Pigment external on thallus and/or ascomata. 25

L23a. Ascospores 8 per ascus, 90–105 × 26–36 µm; Malaysia. *Astrothelium deforme* (Fée) Aptroot & Lücking
L23b Ascospores 2 or 4 per ascus. 24

L24a Ascospores 2 per ascus, 110–130 × 30–35 µm; Guyana. *Astrothelium laurerosphaerioides* Aptroot
L24a Ascospores 4 per ascus, up to 330 × 40 µm; French Guiana. *Astrothelium cayennense* Aptroot & Sipman

L25a Ascospores 8 per ascus, 20–25 µm broad; ascomata pseudostromatic, immersed to erumpent, covered with orange pigment, sharply delimited; Brazil. *Astrothelium mesoduplex* Aptroot & M. Cáceres
L25b Ascospores 2–4 per ascus, 25–50 µm broad; ascomata variable. 26

L26a Pigment on ascomata only, absent from thallus; ostiole in part lateral; Caribbean. *Astrothelium chrysoglyphum* (Vain.) Aptroot & Lücking
Ll26b Pigment on thallus only, absent from ascomata; ostiole consistently apical; pantropical. *Astrothelium vezdae* (Makhija & Patw.) Aptroot & Lücking

Key M. *Astrothelium* species with ascospores muriform, ostiole apical, secondary substances absent

M1a Hamathecium inspersed. 2
M1b Hamathecium clear. 20

M2a Ascospores small to medium-sized (30–60 × 11–20 µm). 3
M2b Ascospores large (80–300 × 20–75 µm). 6

M3a Ascomata aggregate into irregular groups or lines, immersed to erumpent, exposed, blackish; ascospores 35–50 × 12–18 µm; pantropical. *Astrothelium subdiscretum* (Nyl.) Aptroot & Lücking
M3b Ascomata solitary or few aggregate, at least partly covered by thallus; ascospores variable. 4

M4a Ascospores 32–45 × 11–13 µm; ascomata aggregate to pseudostromatic, pseudostromata prominent to sessile, covered by thallus; India. *Astrothelium pseudovariatum* (Upreti & Ajay Singh) Aptroot & Lücking
M4b Ascospores 45–70 × 13–20 µm; ascomata solitary to aggregate, if aggregate then whitish. 5

M5a Ascomata solitary, prominent, mostly exposed and brown-black; Brazil. *Astrothelium ambiguum* (Malme) Aptroot & Lücking
M5b Ascomata solitary, erumpent, mostly covered by thallus except for dark ostiolar area; eastern palaeotropical. *Astrothelium indicum* (Upreti & Ajay Singh) Aptroot & Lücking

M6a Ascospores 1–2 per ascus, 210–300 µm long. 7
M6b Ascospores 4–8 per ascus, 80–200(–230) µm long. 8
M6c Ascospores 4 per ascus, 240–260 µm long; Brazil. *Astrothelium flavogigasporum* Aptroot

M7a Ascospores 1 per ascus, 240–300 × 45–50 µm, with thickened median septum; ascomata confluent to irregularly pseudostromatic, immersed-erumpent, with whitish cover around the blackish ostiole; Colombia. *Astrothelium colombiense* Aptroot
M7b Ascospores 2 per ascus, 210–225 × 30–35 µm, without thickened median septum; ascomata solitary, prominent, mostly exposed and brown-black; eastern palaeotropical. *Astrothelium megaleium* (Kremp.) Aptroot & Lücking

M8a Ascospores submuriform, with few longitudinal septa, 17–22 µm broad; ascomata solitary to irregularly confluent, immersed to erumpent, covered by thallus with broad whitish area around the dark ostiolar spot; India. *Astrothelium macrosporum* (Makhija & Patw.) Aptroot & Lücking
M8b Ascospores distinctly muriform, 20–75 µm broad; ascomata variable. 9

M9a Ascospores 80–140 µm long. 10
M9b Ascospores (125–)140–230 µm long, most ascospores distinctly exceeding 140 µm. 12

M10a Ascospores 2–3 times as long as broad (80–100(–125) × 28–48 µm), without thickened median septum; ascomata solitary, erumpent, laterally covered by thallus with ostiolar area exposed, black; neotropical. *Astrothelium effusum* (Aptroot & Sipman) Aptroot & Lücking
M10b Ascospores 4–5 times as long as broad (115–140 × 25–33 µm), with thickened median septum; ascomata variable. 11

ISSN 1868-4173

M11a Ascomata solitary, strongly prominent, completely covered by thallus; eastern palaeotropical. *Astrothelium aurantiacum* (Makhija & Patw.) Aptroot & Lücking
M11b Ascomata solitary to irregularly confluent, immersed to erumpent, covered by thallus but with exposed, dark ostiolar area bordered by whitish rim; eastern palaeotropical. *Astrothelium sikkimense* (Makhija & Patw.) Aptroot & Lücking

M12a Ascospores with thickened median septum. 13
M12b Ascospores without thickened median septum. 17

M13a Ascomata distinctly pseudostromatic, pseudostromata prominent to sessile, with whitish cover; thallus smooth to uneven; Indonesia. *Astrothelium alboverrucoides* Aptroot
M13b Ascomata solitary; thallus variable. 14

M14a Thallus smooth to uneven; ascomata prominent, exposed and blackish; pantropical. *Astrothelium flavomeristosporum* Aptroot
M14b Thallus verrucose-papillose or bullate; ascomata immersed, covered by thallus. 15

M15a Thallus coarsely bullate, gall-like; ascomata immersed in large thallus verrucae, completely covered by thallus; Philippines. *Astrothelium philippinense* Aptroot & Schumm
M15b Thallus verrucose-papillose; ascomata variable but ostioles partly lateral. 16

M16a Hamathecium orange; ascospores 26–34 µm broad; Papua New Guinea. *Astrothelium papillosum* (P.M. McCarthy) Aptroot & Lücking
M16b Hamathecium uncoloured; ascospores 35–45 µm broad; Venezuela. *Astrothelium komposchii* Aptroot

M17a Ascospores 135–165 µm long; thallus uneven-verrucose; ascomata solitary to irregularly confluent, erumpent, covered by thallus, uneven-verrucose; Brazil. *Astrothelium irregulare* (Müll. Arg.) Aptroot & Lücking
M17b Ascospores (140–)165–230 µm long; thallus and ascomata variable. 18

M18a Hamathecium yellow; thallus bullate; ascomata solitary, prominent, beaked; neotropical. *Astrothelium flavomegaspermum* Aptroot & Etayo
M18b Hamathecium hyaline. 19

M19a Thallus bullate; ascomata solitary to confluent, immersed in coarse thallus verrucae, covered by thallus, with yellowish ostiolar rim; Ecuador. *Astrothelium flavostiolatum* Aptroot

M19b Thallus smooth to uneven; ascomata solitary, prominent, covered by thallus except for dark ostiolar area usually bordered by a whitish rim; pantropical. *Astrothelium megaspermum* (Mont.) Aptroot & Lücking

M20a Ascospores small (30–60 × 11–20 µm). 21
M20b Ascospores medium-sized to large (60–280 × (15–)20–65 µm). 22

M21a Ascospores 30–45 × 11–13 µm; ascomata pseudostromatic, pseudostromata (strongly) prominent, covered by thallus except for ostioles; neotropical. *Astrothelium variatum* (Nyl.) Aptroot & Lücking
M21b Ascospores 50–60 × 15–20 µm; ascomata solitary, prominent, covered by thallus; eastern palaeotropical. *Astrothelium keralense* (Upreti & Ajay Singh) Aptroot & Lücking

M22a Ascospores 200–280 × 50–65 µm, 2 per ascus; ascomata irregularly grouped to diffusely pseudostromatic, pseudostromata erumpent, completely covered by thallus; neotropical. *Astrothelium gigantosporum* (Müll. Arg.) Aptroot & Lücking
M22b Ascospores 70–195 × 14–45 µm, (2–)4–8 per ascus; ascomata variable. 23

M23a Ascospores I+ blue. 24
M23b Ascospores I-negative. 25

M24a Ascospores I+ violet-blue, 125–170 × 27–40 µm; Bolivia. *Astrothelium amylosporum* Flakus & Aptroot
M24b Ascospores I+ pale blue, 85–100 × 20–24 µm; Thailand. *Astrothelium palaeoexostemmatis* Sipman & Aptroot

M25a Ascospores with thickened median septum. 26
M25b Ascospores without thickened median septum. 27

M26a Ascospores 4 per ascus, 145–175 × 30–35 µm; thallus strongly bullate; ascomata solitary to irregularly confluent, immersed in bullate thallus warts and completely covered by thallus except for protruding ostiole; Brazil. *Astrothelium tetrasporum* Aptroot & M. Cáceres
M26b Ascospores (6–)8 per ascus, (85–)100–170 × 23–33 µm; thallus smooth to uneven; ascomata solitary to diffusely pseudostromatic, prominent, whitish surrounding the black ostiolar area; India. *Astrothelium alboverrucum* (Makhija & Patw.) Aptroot & Lücking

M27a Ascospores 1–4 per ascus. 28
M27b Ascospores 8 per ascus. 31

ISSN 1868-4173

M28a Ascospores 1 per ascus, 125–148 µm long; Brazil. *Astrothelium unisporum* Aptroot & M. Cáceres
M28b Ascospores 1–4 per ascus. 29

M29a Ascomata distinctly pseudostromatic, pseudostromata erumpent to prominent, with white cover, sharply delimited from thallus; ascospores 80–140 × 25–35 µm; pantropical. *Astrothelium sphaerioides* (Müll. Arg.) Aptroot & Lücking
M29b Ascomata irregularly confluent to diffusely pseudostromatic, erumpent to prominent, either fully exposed and brown-black or covered by olive-green thallus; ascospores 120–180(–200) × 30–50(–60) µm. 30

M30a Ascomata erumpent, fully exposed and brown-black; ascospores 120–175 × 30–45 µm; neotropical. *Astrothelium subdisjunctum* (Mont.) Aptroot & Lücking
M30b Ascomata prominent, covered by thallus except dark ostiole bordered by a white rim, with one to several ascomata per thallus wart and ostioles often pointing in different directions; ascospores 160–180(–200) × 40–50(–60) µm; Central America. *Astrothelium nicaraguense* Lücking, M.P. Nelsen & T. Orozco

M31a Ascospores 150–190 × 40–45 µm; ascomata solitary or few grouped, covered by thallus; neotropical (Brazil and Bolivia). *Astrothelium octosporoides* Aptroot & Lücking
M31b Ascospores 60–150 × 15–30 µm; ascomata variable. 32

M32a Ascomata solitary, immersed to erumpent, covered by thallus except for dark ostiolar area surrounded by irregular whitish rim; ascospores not exceeding 80 µm in length; neotropical. *Astrothelium exostemmatis* (Müll. Arg.) Aptroot & Lücking
M32b Ascomata irregularly confluent to pseudostromatic, erumpent to prominent, fully exposed; ascospores usually exceeding 80 µm in length; Brazil. *Astrothelium chapadense* (Malme) Aptroot & Lücking

Key N. *Astrothelium* species with ascospores muriform, ostiole lateral, secondary substances present on thallus and/or ascoma

N1a Lichexanthone present; thallus and/or ascomata UV+ yellow; pigment(s) present or absent. 2
N1b Lichexanthone absent; pigment(s) always present, thallus and ascomata usually K+ red to purple and UV+ orange or red. 17

N2a Hamathecium inspersed; ascomata with separate or fused ostioles. 3
N2b Hamathecium clear; ascomata with fused ostioles. 6

N3a Ascospores 80–115 × 25–35 µm, 4 per ascus; lichexanthone in medulla; ascomata with separate ostioles, immersed, covered by thallus; Ecuador. *Astrothelium lucidomedullatum* Aptroot
N3b Ascospores 150–220 × 40–65 µm, 2 or 8 per ascus; lichexanthone external on thallus and/or ascomata. 4

N4a Ascospores 8 per ascus, 40–50 µm broad; ascomata with separate ostioles,; lichexanthone on thallus and ascomata. 5
N4b Ascospores 2 per ascus, 50–65 µm broad; ascomata with fused ostioles, groups of fused ascomata dispersed, covered by thallus except ostioles; lichexanthone on ascomata only; Brazil. *Astrothelium megalostomum* (Vain.) Aptroot & Lücking

N5a Ascomata covered by thallus except for broad black ostiolar area; Bolivia. *Astrothelium carrascoense* Flakus, Kukwa & Aptroot
N5b Ascomata with separate ostioles, covered by thallus, without black ostiolar area; Brazil. *Astrothelium aureoirregulare* Aptroot & Gumboski

N6a Ascospores small (35–45 × 15–19 µm). 7
N6b Ascospores large (85–250 × 19–65 µm). 8

N7a Only ostiole UV+ yellow; fused ascomata pseudostromatic, pseudostromata erumpent, with white cover contrasting with olive-green thallus; Brazil. *Astrothelium eustomurale* Aptroot & M. Cáceres
N7b Entire pseudostroma UV+ yellow; fused ascomata pseudostromatic, pseudostroma immersed, with white cover contrasting with olive-brown thallus; Guyana. *Astrothelium lucidostromum* Aptroot

N8a Ascospores 150–250 × 30–65 µm, (1–)2 per ascus; ascomata with fused ostioles. 9
N8b Ascospores 85–150 × 19–45 µm, 2 or 8 per ascus; ascomata variable. 12

N9a Fused ascomata solitary, immersed and covered by thallus; lichexanthone on thallus and ascomata; neotropical. *Astrothelium sepultum* (Mont.) Aptroot & Lücking
N9b Fused ascomata pseudostromatic, pseudostromata immersed, whitish and contrasting with olive-green thallus; thallus bullate-bumpy; lichexanthone on pseudostromata only. 10

N10a Lichexanthone only on the thallus; pseudostroma with orange pigment; Brazil. *Astrothelium astrolucidum* Aptroot & M. Cáceres
N10b Lichexanthone only on pseudostromata; pseudostromata without pigment. 11

N11a Thallus dominated by finely and densely cracked pseudostromata, with thallus bumps dispersed; ascomata completely immersed in pseudostromata, hardly visible; Panama. *Astrothelium cryptolucens* Lücking, M.P. Nelsen & N. Salazar
N11b Thallus dominated by vegetative part forming large bumps separating the smooth pseudostromata; ascomata immersed to erumpent with the ostiolar area broadly exposed and black; neotropical. *Astrothelium norisianum* Lücking, M.P. Nelsen & Aptroot

N12a Ascospores 85–130 µm long, 2 per ascus; lichexanthone internal in medulla; fused ascomata solitary to irregularly confluent, immersed to erumpent, covered by thallus except dark ostiolar area surrounded by irregular whitish rim; neotropical. *Astrothelium consimile (*Müll. Arg.) Aptroot & Lücking
N12b Ascospores 100–150 µm long, 8 per ascus; lichexanthone external on ascomata; ascomata variable. 13

N13a Ascospores 30–45 µm broad. 14
N13b Ascospores 17–23 µm broad; ascomata variable. 15

N14a Ascomata solitary; lichexanthone on pseudostromata; neotropical. *Astrothelium defossum* (Müll. Arg.) Aptroot & Lücking
N14b Ascomata fused; lichexanthone only on ostiole; Brazil. *Astrothelium megeustomurale* M. Cáceres & Aptroot

N15a Ascomata solitary, immersed to erumpent, covered by thallus except dark ostiolar area; thallus shallowly folded; Brazil. *Astrothelium octosporum* (Vain.) Aptroot & Lücking
N15b Ascomata irregularly confluent to grouped, erumpent to prominent, completely covered by thallus; thallus smooth to uneven. 16

N16a Ostioles visible as sessile, broadly white papillae; Brazil. *Astrothelium megeustomum* Aptroot & Fraga Jr.
N16b Ostioles visible as minute, immersed dark pores; neotropical. *Astrothelium confluens* (Müll. Arg.) Aptroot & Lücking

N17a Ascospores (small to) medium-sized (35–80 × 12–25 µm). 18
N17b Ascospores large (75–200 × 22–50 µm). 20

N18a pigment external on ascomata only, orange. 19
N18b Ascomata pseudostromatic, pseudostromata immersed, whitish, forming irregular to reticulate lines, well delimited from thallus; pigment external on thallus only,

orange; French Guiana. *Astrothelium praetervisum* (Müll. Arg.) Aptroot & Lücking

N19a Ascomata pseudostromatic, with fused ostioles; Brazil. *Astrothelium muriconicum* Aptroot
N19b Ascomata solitary, immersed to erumpent, covered by thallus except dark ostiolar area surrounded by whitish rim; neotropical. *Astrothelium subaequans* Müll. Arg.

N20a Pigment orange, external on ascomata or internal in medulla. 21
N20b Pigment red, external on ascomata. 23

N21a Pigment internal in medulla of ascomata; neotropical. *Astrothelium amazonum* (R.C. Harris) Aptroot & Lücking
N21b Pigment external on ascomata. 22

N22a Ascospores 100–170 × 30–40 µm, regularly muriform; ascomata pseudostromatic, pseudostromata erumpent, with orange cover; Brazil. *Astrothelium carassense* Lücking, M.P. Nelsen & Marcelli
N22b Ascospores 90–110 × 22–28 µm, with few longitudinal septa, submuriform; ascomata solitary to irregularly grouped, covered by thallus except ostiole; neotropical. *Astrothelium* diplocarpum Nyl.

N23a Ascospores 100–130 × 25–30 µm, 8 per ascus; ascomata solitary, immersed to erumpent, irregularly bumpy with central red ostiole; neotropical. *Astrothelium purpurascens* (Müll. Arg.) Aptroot & Lücking
N23b Ascospores 150–200 × 40–50 µm, 2 per ascus; Brazil. *Astrothelium isabellinum* Eschw.

Key O. *Astrothelium* species with ascospores muriform, ostiole lateral, secondary substances absent

O1a Ascomata solitary, with separate ostioles. 2
O1b Ascomata fused, with joint ostioles. 18

O2a Hamathecium inspersed. 3
O2b Hamathecium clear. 11

O3a Ascospores 30–33 × 9.5–10.5 µm; Brazil. *Astrothelium quasimamillanum* Aptroot & C.O. Mendonça
O3b Ascospores 70–230 × 20–55 µm. 4

ISSN 1868-4173

O4a Ascospores 70–100 × 20–35 µm; thallus lacking pseudocyphellae. 5
O4b Ascospores (85–)100–230 × 25–55 µm, if less than 100 µm then thallus with pseudocyphellae. 7

O5a Ascospores curved, 74–90 × 25–34 µm; ascomata dispersed, immersed to erumpent and covered by thallus except ostioles; Brazil. *Astrothelium curvatum* Aptroot & M. Cáceres
O5b Ascospores straight. 6

O6a Ascospores 8 per ascus; thallus smooth to uneven; ascomata dispersed to irregularly confluent, immersed to erumpent, laterally covered by thallus but otherwise exposed and black, surrounded by irregular whitish rim; pantropical. *Astrothelium santessonii* (Letr.-Gal.) Aptroot & Lücking
O6b Ascospores 2 per ascus; thallus verrucose-papillose; ascomata dispersed to irregularly confluent, sessile, covered by thallus except the strongly protruding ostiole; Costa Rica. *Astrothelium minicecidiogenum* Aptroot & Sipman

O7a Ascospores 250–350 × 40–60 µm, 1 per ascus; thallus irregularly bullate-bumpy; ascomata solitary, immersed, hardly visible; Panama. *Astrothelium megacrypticum* Lücking, M.P. Nelsen & N. Salazar
O7b Ascospores 85–230 × 25–60 µm, (1–)2–8 per ascus; ascomata variable. 8

O8a Ascospores 8 per ascus, 200–230 × 30–40 µm, about 6–7 times as long as broad; Brazil. *Astrothelium longisporum* Aptroot, J.R. Silva & M. Cáceres
O8b Ascospores 1–2 per ascus. 9

O9a Ascospores 170–340 × 30–60 µm, about 5–6 times as long as broad, 2 per ascus; neotropical. *Astrothelium puiggarii* (Müll. Arg.) Aptroot & Lücking
O9b Ascospores 85–175 × 25–55 µm, about 2.5–4 times as long as broad. 10

O10a Thallus with pseudocyphellae; ascomata laterally covered by thallus, with black ostiolar area surrounded by black rim; Bolivia. *Astrothelium pyrenuliforme* Flakus & Aptroot
O10b Thallus lacking pseudocyphellae; ascomata solitary, exposed and pure black; Ecuador. *Astrothelium ecuadoriense* Aptroot

O11a Ascospores 210–230 µm long, 1 per ascus; thallus irregularly verrucose; ascomata dispersed, immersed, hardly visible; São Tomé. *Astrothelium lugescens* (Nyl.) Aptroot & Lücking
O11b Ascospores 70–200 µm long, (1–)2–8 per ascus; ascomata variable. 12

O12a Ascospores 70–130 × 20–36 µm. 13

O12b Ascospores 120–200 × 30–50(–60) µm. 15

O13a Ascospores 2 per ascus; ascomata irregularly confluent to pseudostromatic, immersed to erumpent, covered by thallus except black ostiolar area surrounded by irregular whitish rim; India. *Astrothelium andamanicum* (Makhija & Patw.) Aptroot & Lücking
O13b Ascospores 4–8 per ascus; ascomata dispersed. 14

O14a Ascospores with distinctly thickened median septum, 4–8 per ascus; thallus smooth to uneven; ascomata erumpent; pantropical. *Astrothelium tenue* (Aptroot) Aptroot & Lücking
O14b Ascospores lacking thickened median septum, 4 per ascus; thallus gall-like, coarsely bullate to folded; ascomata solitary, immersed, visible only by the dark ostiolar area; Guyana. *Astrothelium guianense* Aptroot

O15a Ascospores I+ violet; thallus strongly bullate; ascomata immersed in thallus warts, completely covered by thallus; Bolivia. *Astrothelium bullatum* Flakus & Aptroot
O15b Ascospores I−. 16

O16a Ascospores 8 per ascus; eastern palaeotropical. *Astrothelium superbum* (Fr.) Aptroot & Lücking
O16b Ascospores 2–4 per ascus. 17

O17a Ascomata usually single in thalline warts, warts up to 1 mm diam., with broad, diffusely grey-black ostiolar area; ostioles always eccentric; neotropical. *Astrothelium campylocartilagineum* Aptroot & Lücking
O17b Ascomata 1–5 in thalline warts, warts up to 2 mm diam., with narrow, brown-black ostiolar area surrounded by a whitish rim; ostioles pointing in various directions including apical ones; Nicaragua. *Astrothelium nicaraguense* Lücking, M.P. Nelsen & T. Orozco

O18a Ascospores small to medium-sized (30–80 × 16–26 µm). 19
O18b Ascospores large (125–230 × 28–80 µm). 23

O19a Hamathecium inspersed; ascospores 50–65 µm long; groups of fused ascomata irregularly confluent to pseudostromatic, immersed to erumpent, covered by thallus except black ostiolar area surrounded by irregular whitish rim; Brazil. *Astrothelium testudineum* Aptroot & M. Cáceres
O19b Hamathecium clear; ascospores 30–52 or 70–80 µm long. 20

ISSN 1868-4173

O20a Ascospores medium-sized (70–80 × 20–25 µm); thallus uneven-verrucose; groups of fused ascomata pseudostromatic, pseudostromata prominent, covered by thallus except for dark, papilliform ostiole; Brazil. *Astrothelium mediocrassum* Aptroot
O20b Ascospores small (30–52 × 16–22 µm); thallus and ascomata variable. 21

O21a Groups of fused ascomata hemispherical; Brazil. *Astrothelium citrisporum* Aptroot, Oliveira-Junior & M. Cáceres
O21b Groups of fused ascomata immersed to erumpent. 22

O22a Groups of fused ascomata pseudostromatic, pseudostromata immersed, with white cover, forming irregular to reticulate lines, contrasting with thallus; neotropical. *Astrothelium cinereum* (Müll. Arg.) Aptroot & Lücking
O22b Groups of fused ascomata dispersed, immersed-erumpent, hardly visible; neotropical. *Astrothelium leioplacum* (Müll. Arg.) Aptroot & Lücking

O23a Ascospores 215–230 µm long, 1 per ascus; groups of fused ascomata dispersed, erumpent, completely covered by thallus; on rock; Brazil. *Astrothelium saxicola* (Malme) Aptroot & Lücking
O23b Ascospores 125–200 µm long, 8 per ascus. 24

O24a Ascospores 2/ascus, with median thickening; hamathecium only inspersed above; Brazil. *Astrothelium dimidioinspersum* Aptroot & M. Cáceres
O24b Ascospores 8/ascus, with or without median thickening. 25

O25a Ascospores with median thickening; groups of fused ascomata dispersed, immersed, hardly visible; Brazil. *Astrothelium flavomurisporum* Aptroot & M. Cáceres
O25b Ascospores lacking thickening; groups of fused ascomata dispersed to irregularly confluent, covered by thallus except whitish ostiolar area; neotropical. *Astrothelium cecidiogenum* (Aptroot & Lücking) Aptroot & Lücking

Key P. *Bathelium* species

P1a Ascospores transversely septate. 2
P1b Ascospores muriform. 7

P2a Ascospores 13–17-septate, 65–105 µm long; ostiolar area UV+ yellow, with lichexanthone; Bolivia. *Bathelium flavostiolatum* Flakus & Aptroot
P2b Ascospores 3–9-septate, 18–40 µm long; UV−. 3

P3a Ascospores 3(–5)-septate, 18–25(–30) µm long. 4

P3b Ascospores 5–9-septate, 25–40 µm long. 6

P4a Internal pseudostroma pigment yellow, K+ persistently yellow; ascomata distinctly pseudostromatic; USA. *Bathelium carolinianum* (Tuck.) R.C. Harris
P4b Internal pseudostroma pigment yellow-orange to reddish brown, K+ orange-red or purple-violet; ascomata variable. 5

P5a Internal pseudostroma pigment yellow-orange, K+ purple-violet; ascospores 18–21 ×6–7 µm long; ascomata pseudostromatic; South Africa. *Bathelium austroafricanum* (Zahlbr.) Aptroot & Lücking
P5b Internal pseudostroma pigment reddish brown, K+ orange-red; ascospores 20–30 × 6–7 µm long; ascomata solitary to irregularly confluent; eastern palaeotropical. *Bathelium porinosporum* Lücking, M.P. Nelsen & Gueidan

P6a Ostioles usually white-pruinose; ascospores 5–7-septate, 25–32 µm long; eastern palaeotropical. *Bathelium albidoporum* (Makhija & Patw.) R.C. Harris
P6b Ostioles non-pruinose, black; ascospores 5–9-septate, 30–40 µm long; palaeotropical. *Bathelium nigroporum* (Makhija & Patw.) Aptroot & Lücking

P7a Thallus and pseudostromata UV+ yellow, with lichexanthone. 8
P7b Thallus and pseudostromata UV− or UV+ red when pigment is present, lichexanthone absent. 9

P8a Hamathecium inspersed; Brazil. *Bathelium pruinolucens* Aptroot & Lücking
P8b Hamathecium not inspersed; Bolivia and Brazil. *Bathelium pruinosum* Flakus, Kukwa & Aptroot

P9a Internal pigment of pseudostromata dark red, K+ green. 10
P9b Internal pigment of pseudostromata yellow-orange, K+ purple. 11

P10a Pseudostromata erumpent, covered with orange pigment and with dark ostioles surrounded by paler rim; Bolivia and Brazil. *Bathelium mirabile* Flakus, Kukwa & Aptroot
P10b Pseudostromata prominent to sessile, dark brown and with strongly contrasting, whitish ostiolar area; Bolivia. *Bathelium boliviense* Flakus & Aptroot

P11a Ascospores 40–80 µm long.12
P11b Ascospores 100–200 µm long. 15

P12a Hamathecium fully inspersed; Bolivia. *Bathelium inspersomastoideum* Flakus & Aptroot
P12b Hamathecium clear except for ostiolar area. 13

ISSN 1868-4173

P13a Ascospores (50–)60–80 µm long. *Bathelium mastoideum* Afzel. ex Ach.
P13b Ascospores 40–55 µm long. 14

P14a Thallus smooth to uneven; pantropical. *Bathelium madreporiforme* (Eschw.) Trevis.
P14b Thallus verrucose-bullate; pantropical. *Bathelium tuberculosum* (Makhija & Patw.) R.C. Harris

P15a Ascospores 100–120 µm long; pantropical. *Bathelium lineare* (C.W. Dodge) R.C. Harris
P15b Ascospores 130–200 µm long; Sierra Leone. *Bathelium sphaericum* (C.W. Dodge) R.C. Harris

Key Q; pantropical. *Bogoriella* species

Q1a Ascospores becoming distinctly muriform, with thin eusepta and rectangular lumina. 2
Q1b Ascospores 1-septate to rarely 3-septate or submuriform, with thin eusepta or with irregularly thickened endospore and distosepta. 4

Q2a Ascospores becoming thick-walled and with thickened median septum, 28–45 µm long; Bolivia. *Bogoriella chiquitana* (Flakus, Kukwa & Aptroot) Aptroot & Lücking
Q2b Ascospores remaining thin-walled, 20–35 µm long. 3

Q3a Ascospores 20–30 × 12–16 µm, about 1.5–2 times as long as broad; pantropical. *Bogoriella decipiens* (Müll. Arg.) Aptroot & Lücking
Q3b Ascospores 20–35 × 7–10 µm, about 3–3.5 times as long as broad; China. *Bogoriella pandanicola* (S.N. Zhang & K.D. Hyde) S.N. Zhang, Aptroot & Lücking

Q4a Ascospores with irregularly thickened endospore and distosepta, 1-septate or rarely 3-septate to submuriform; lumina irregular to halter-shaped. 5
Q4b Ascospores with thin eusepta, 1-septate or rarely 3-septate; lumina rectangular. 8

Q5a Ascospores (1–)3-septate to appearing submuriform, 27–45 × 10–17 µm. 6
Q5b Ascospores 1-septate but cells with irregular endospore invaginations, 15–29 × 6–14 µm. 7

Q6a Ascospores mostly distinctly 3-septate, occasionally 1-septate or with additional longitudinal septa; pantropical. *Bogoriella queenslandica* (Müll. Arg.) Aptroot & Lücking
Q6b Ascospores with one distinct, rather dark central septum and the two cells with irregular endospore invaginations giving them the appearance of a (sub-)muriform division; Brazil. *Bogoriella complexoluminata* Aptroot & Lücking

Q7a Ostiole with red pigment; ascospores 23–29 × 10–14 µm, secondary endospore invaginations developing late; Hong Kong. *Bogoriella isthmospora* (Aptroot) Lücking, R. Miranda & Aptroot
Q7b Ostiole lacking pigment; ascospores 15–18 × 6–8 µm, secondary endospore invaginations developing soon; Caribbean. *Bogoriella rubrostoma* (Aptroot) Aptroot & Lücking

Q8a Ostiole apical. 9
Q8b Ostiole lateral. 18

Q9a Hamathecium inspersed. 10
Q9b Hamathecium clear. 12

Q10a Ascospores 24–27 × 8–11 µm; neotropical. *Bogoriella oleosa* (Aptroot) Aptroot & Schumm
Q10b Ascospores 30–45 × 10–15 µm. 11

Q11a Ascomata 0.5–0.7 mm diam; Venezuela. *Bogoriella xanthonica* (Komposch, Aptroot & Hafellner) Aptroot & Lücking
Q11b Ascomata 0.7–1.1 mm diam; Venezuela. *Bogoriella macrocarpa* (Komposch, Aptroot & Hafellner) Aptroot & Lücking

Q12a Ascospores becoming 3-septate; pantropical. *Bogoriella obovata* (Stirt.) Aptroot & Lücking
Q12b Ascospores remaining 1-septate. 13

Q13a Ascospores 28–40 µm long; Brazil. *Bogoriella megaspora* (Aptroot & M. Cáceres) Aptroot & Lücking
Q13b Ascospores 17–27 µm long. 14

Q14a Black prothallus line present. 15
Q14b Black prothallus line absent. 16

Q15a Asci 10–20 µm broad; eastern palaeotropical. *Bogoriella conothelena* (Nyl.) Aptroot & Lücking

Q15b Asci 20–30 µm broad; pantropical. *Bogoriella thelena* (Ach.) Aptroot & Lücking

Q16a Ascomata 0.8–1 mm diam., basally broadly expanded; pantropical. *Bogoriella confuens* (Müll. Arg.) Aptroot & Lücking
Q16b Ascomata 0.2–0.4 mm diam., basally barely or not expanded. 17

Q17a Ascomata conical; Brazl. *Bogoriella pachytheca* (Sacc. & P. Syd.) Aptroot & Lücking
Q17b Ascomata fattened; pantropical. *Bogoriella modesta* (Müll. Arg.) Aptroot & Lücking

Q18a Thallus UV-negative; Puerto Rico. *Bogoriella triangularis* (Aptroot) Aptroot & Lücking
Q18b Thallus UV+ yellow; Brazl. *Bogoriella xantholateralis* Aptroot

Key R. *Constrictolumina* species

R1a Ascospores 17–23 × 5–8 µm, 1–3-septate. 2
R1b Ascospores (20–)22–46 × 7–24 µm, 1-septate. 3

R2a Ascomata aggregate; neotropical. *Constrictolumina esenbeckiana* (Fée) Lücking, M.P. Nelsen & Aptroot
R2b Ascomata solitary; pa; Dominica; neotropical ntropical. *Constrictolumina cinchonae* (Ach.) Lücking, M.P. Nelsen & Aptroot

R3a Ascospores 40–46 × 16–24 µm. *Constrictolumina porospora* (Vain.) Lücking, M.P. Nelsen & Aptroot
R3b Ascospores 20–30 × 7–16 µm. 4

R4a Ascomata aggregate; ascospores 3-septate; neotropical. *Constrictolumina leucostoma* (Müll. Arg.) Lücking, M.P. Nelsen & Aptroot
R4b Ascomata solitary; ascospores 1(–3)-septate; pantropical. *Constrictolumina planorbis* (Ach.) Lücking, M.P. Nelsen & Aptroot

Key S. *Dictyomeridium* species

S1a Thallus UV+ yellow. 2
S1b Thallus UV−. 5

S2a Ascomata deeply immersed in the bark, ascospores 2 per ascus, 45–70 × 17–25 µm; Brazil. *Dictyomeridium immersum* (Aptroot, A.A. Menezes & M. Cáceres) Aptroot, M.P. Nelsen & Lücking
S2b Ascomata erumpent, ascospores 8 per ascus. 3

S3a Ascospores 25–35 × 12–17 µm; Bolivia. *Dictyomeridium lueckingii* Flakus & Aptroot
S3b Ascospores over 35 µm long. 4

S4a Ascospores 35–54 × 12–19 µm; pantropical. *Dictyomeridium proponens* (Nyl.) Aptroot, M.P. Nelsen & Lücking
S4b Ascospores 55–75 × 19–21 µm; Brazil. *Dictyomeridium paraproponens* (Aptroot, M. Cáceres & E.L. Lima) Aptroot, M.P. Nelsen & Lücking

S5a Ascospores 55–80 × 17–25 µm; eastern palaeotropical. *Dictyomeridium campylothelioides* (Aptroot & Sipman) Aptroot, M.P. Nelsen & Lücking
S5b Ascospores under 55 µm long. 6

S6a Ostiole with red, KOH+ green pigment inside, ascospores 33–55 × 11–15 µm; Brazil. *Dictyomeridium isohypocrellinum* (Xavier-Leite, M. Cáceres & Aptroot) Aptroot, M.P. Nelsen & Lücking
S6b Ostiole without red pigment, ascospores 33–55 × 12–21 µm; pantropical. *Dictyomeridium amylosporum* (Vain.) Aptroot, M.P. Nelsen & Lücking

Key T. *Macroconstrictolumina* species

T1a Ascospores 18–30 × 7–10(–12) µm; pantropical. *Macroconstrictolumina lyrata* (R.C. Harris) Lücking, R. Miranda & Aptroot
T1b Ascospores 30–48 × 10–16(–24) µm. 2

T2a Ostiole lateral; ascospores 1-septate; Brazil. *Macroconstrictolumina megalateralis* Aptroot
T2b Ostiole apical; ascospores 1(–3)-septate. 3

T3a Ascospores 27–37 × 10–12 µm, often 3-septate; pantropical. *Macroconstrictolumina majuscula* (Nyl.) Lücking, R. Miranda & Aptroot
T3b Ascospores 40–50 × 15–20 µm, mostly 1-septate; pantropical. *Macroconstrictolumina malaccitula (Nyl.) Lücking, R. Miranda & Aptroot*

Key U. *Marcelaria* species

U1a Ascomata (and often thallus) bright red, UV−; hamathecium clear; ascospores 110–170 µm long, amyloid; Neotropics and African Palaeotropics. *Marcelaria purpurina* (Nyl.) Aptroot, Nelsen & Parnmen
U1b Ascomata (and often thallus) (orange-)yellow, UV+ yellow; hamathecium densely inspersed; ascospores 50–80 µm long, non-amyloid (rarely dextrinoid); eastern Palaeotropics. 2

U2a Thallus yellow-pruinose; ascospores 8 per ascus; ostiolar region narrow. *Marcelaria benguelensis* (Müll. Arg.) Aptroot, Nelsen & Parnmen
U2b Thallus non-pruinose; ascospores 2 per ascus; ostiolar region broad, flat. *Marcelaria cumingii* (Mont.) Aptroot, Nelsen & Parnmen

Key V. *Nigrovothelium* species

V1a Hamathecium inspersed; pantropical. *Nigrovothelium inspersotropicum* Aptroot & Diederich
V1b Hamathecium clear. 2

V2a Thallus smooth to uneven; pantropical. *Nigrovothelium tropicum (*Ach.) Lücking, M.P. Nelsen & Aptroot
V2b Thallus verrucose-bullate; India. *Nigrovothelium bullatum* Lücking, Upreti & Lumbsch

Key W. *Polymeridium* species

W1a Ascospores transversely septate. 2
W1b Ascospores submuriform to muriform. 49

W2a Ascospores regularly 3-septate. 3
W2b Ascospores irregularly (3–)4–17-septate. 28

W3a Thallus UV+ yellow, with lichexanthone. 4
W3b Thallus UV−, without substances or rarely with medullary pigments or pruina on ascomata. 14

W4a Hamathecium inspersed. 5
W4b Hamathecium clear. 7

W5a Ascospores 24–33 × 6–10 µm; pantropical. *Polymeridium subvirescens* (Nyl.) R.C. Harris
W5b Ascospores 17–23 × 5–8 µm. 6

W6a Ascospores 17–20 × 5–7 µm; pantropical. *Polymeridium pyrenuloides* (Müll. Arg.) Aptroot
W6b Ascospores 20–23 × 6–8 µm; pantropical. *Polymeridium albidovarians* Aptroot

W7a Ostioles eccentric, separate or fused; ascospores 24–33 µm long. 8
W7b Ostioles apical; ascospores variable. 9

W8a Ostioles fused; Venezuela. *Polymeridium pyrenastroides* R.C. Harris ex Aptroot
W8b Ostioles separate; neotropical. *Polymeridium stramineoatrum* (Vain.) Aptroot

W9a Ascospores IKI+ violet, 16–23 × 5–7 µm; Brazil. *Polymeridium amyloideum* R.C. Harris
W9b Ascospores IKI−. 10

W10a Ascospores 13–20 × 4–6 µm. 11
W10b Ascospores 19–33 × 6–11 µm. 12

W11a Ascospores 13–17 µm long; pantropical. *Polymeridium albopruinosum* (Makhija & Patw.) Aptroot
W11b Ascospores 16–20 µm long; pantropical. *Polymeridium bengoanum* (Vain.) Aptroot

W12a Ascomata mostly covered by thallus, with black ostioles bordered by white ring; ascospores relatively broad, 17–25 × 7–11 µm, c. 2.5 times as long as broad; Australia. *Polymeridium xanthoreagens* Aptroot
W12b Ascomata pure black; ascospores narrower, 20–33 × 6–10 µm, about 3 times as long. 13

W13a Ascospores 20–23 × 6–8 µm; Brazil. *Polymeridium albidoreagens* Aptroot, A.A. Menezes & M. Cáceres
W13b Ascospores 25–33 × 7–10 µm; pantropical. *Polymeridium catapastum* (Nyl.) R.C. Harris

W14a Hamathecium inspersed; ostioles apical. 15
W14b Hamathecium clear; ostioles variable. 20

W15a Ascospores 24–34 µm long; ascomata laterally covered by thallus. 16

W15b Ascospores 12–24 µm long; ascomata variable. 17

W16a Ascospores with rough wall, 26–34 µm long; blackish ostiolar area broad; eastern palaeotropical. *Polymeridium glaucoatrum* (Vain.) R.C. Harris
W16b Ascospores smooth, 24–30 µm long; blackish ostiolar area narrow; eastern palaeotropical. *Polymeridium suffusum* (Knight) Aptroot

W17a Ascospores 12–20 × 3–6 µm. 18
W17b Ascospores 20–24 × 5–10 µm. 19

W18a Ascospores 12–16 µm long; neotropical. *Polymeridium contendens* (Nyl.) R.C. Harris
W18b Ascospores 16–20 µm long; pantropical. *Polymeridium inspersum* Aptroot

W19a Ascomata with ostioles bordered by white ring; ascospores relatively broad, 20–24 × 7–10 µm, c. 2.5–3 times as long as broad; pantropical. *Polymeridium tribulationis* Aptroot
W19b Ascomata pure black; ascospores narrower, 20–23 ×5–7 µm, c. 3–4 times as long as broad; pantropical. *Polymeridium siamense* (Vain.) Aptroot

W20a Ascomata with internal or external, yellow-orange or red pigment. 21
W20b Ascomata without pigments. 22

W21a Ascomata with internal, yellow-orange, K+ purple pigment; ascospores 30–34 × 9–10 µm; Brazil. *Polymeridium endocrocinum* R.C. Harris
W21b Ascomata with external, red, K+ red pruina; ascospores 17–19 × 4–5 µm; Puerto Rico. *Polymeridium rhodopruinosum* Aptroot & Mercado Diaz

W22a Ostioles lateral. 23
W22b Ostioles apical. 24

W23a Ascomata grouped with fused ostioles; ascospores 26–30 ×8–10 µm; Brazil. *Polymeridium biloculare* R.C. Harris
W23b Ascomata solitary with separate ostioles; ascospores 30–35 ×12–13 µm; neotropical. *Polymeridium simulans* R.C. Harris

W24a Ascospores 24–33 µm long; eastern palaeotropical. *Polymeridium catapastoides* Aptroot
W24b Ascospores 11–27 µm long. 25

W25a Ascospores 11–16 × 3–6 µm; pantropical. *Polymeridium microsporum* (Makhija & Patw.) Aptroot

W25b Ascospores longer. 26

W26a Ascospores (15–)16–20 × 5.0–7.5 µm; pantropical. *Polymeridium subcinereum* (Nyl.) R.C. Harris
W26b Ascospores 19–27 × 6–11 µm. 27

W27a Ostiole flat and with white ring; ascospores 19–27 × 6.5–11.0 µm; eastern palaeotropical.
Polymeridium sulphurescens (Müll. Arg.) R.C. Harris
W27b Ostiole not flat, black; ascospores (18–)20–23 × 6–8(–9) µm; pantropical. *Polymeridium albidum* (Müll. Arg.) R.C. Harris

W28a Thallus UV+ yellow, with lichexanthone. 29
W28b Thallus UV−, without substances. 38

W29a Hamathecium inspersed. 30
W29b Hamathecium clear. 33

W30a Hamathecium yellow. 31
W30b Hamathecium hyaline. 32

W31a Ascospores 5–7-septate, 32–37 × 10–13 µm; Brazil. *Polymeridium endoflavens* Aptroot, D.S. Andrade & M. Cáceres
W31b Ascospores 8–12-septate, 56–70 × 12–14 µm; Brazil. *Polymeridium longiflavens* Aptroot, Mendonça & M. Cáceres

W32a Ascospores 4–7-septate, 18–28 × 5–7 µm; pantropical. *Polymeridium multiseptatum* Aptroot, A.A. Menezes & M. Cáceres
W32b Ascospores 8–12-septate, 40–50 × 9–11 µm; neotropical. *Polymeridium chioneum* (Mont.) R.C.Harris

W33a Ascomata with lateral ostioles. 34
W33b Ascomata with apical ostioles. 36

W34a Ascomata solitary, ascospores 11–13-septate, 52–88 × 16–19 µm; Panama. *Polymeridium xanthopleurothecium* Aptroot & Etayo
W34b Ascomata grouped with lateral, fused ostioles. 35

W35a Ascospores 28–35 µm long; neotropical. *Polymeridium dithecium* R.C. Harris
W35b Ascospores 30–50 µm long; Bolivia. *Polymeridium xanthoexcentricum* Flakus & Aptroot

ISSN 1868-4173

W36a Ascospores (4–)5(–7)-septate, 19–26 µm long; neotropical. *Polymeridium multiforme* Aptroot
W36b Ascospores 7–13-septate, 28–39 µm long. 37

W37a Thallus corticate; Brazil. *Polymeridium corticatum* A.A. Menezes, M. Cáceres & Aptroot
W37b Thallus ecorticate; neotropical. *Polymeridium alboflavescens* Aptroot

W38a Hamathecium inspersed. 39
W38b Hamathecium clear. 45

W39a Hamathecium yellow. 40
W39b Hamathecium hyaline. 41

W40a Ascospores 8–12-septate, 40–55 × 9–11 µm; ostioles lateral; ; neotropical. *Polymeridium flavothecium* R.C. Harris
W40b Ascospores 6–8-septate, 35–37 × 7.5–8 µm; ostioles apical; Sri Lanka. *Polymeridium fernandoi* Aptroot & Weerakoon

W41a Ostioles lateral; ascospores 3–6-septate, 22–26 × 7–9 µm; India. *Polymeridium refertum* (Stirt.) Aptroot
W41b Ostioles apical; ascospores variable. 42

W42a Ascospores 4–7-septate. 43
W42b Ascospores 7–12-septate. 44

W43a Ascospores with rough wall, 7-septate, 30–33 × 9–10 µm; Venezuela. *Polymeridium neuwirthii* Aptroot
W43b Ascospores smooth, (4–)5(–7)-septate, 18–28 × 4–7 µm; pantropical. *Polymeridium quinqueseptatum* (Nyl.) R.C. Harris

W44a Ascospores 7–11-septate, 25–36 × 5–9 µm; pantropical. *Polymeridium pleiomerellum* (Müll. Arg.) R.C. Harris
W44b Ascospores 8–12-septate, 40–50 × 9–11 µm; neotropical. *Polymeridium costaricense* Aptroot

W45a Ostioles lateral. 46
W45b Ostioles apical. 47

W46a Ascospores 7–9-septate, 40–50 × 8–9 µm; neotropical. *Polymeridium neblinae* R.C. Harris

W46b Ascospores 9–13-septate, (35–)50–75 × 12–16 µm; pantropical. *Polymeridium pleurothecium* R.C. Harris

W47a Ascospores 13–17-septate, 85–150 × 20–30 µm; Argentina. *Polymeridium bambusicola* Aptroot & L.I. Ferraro
W47b Ascospores 4–11-septate, 21–39 × 5–9 µm. 48

W48a Ascospores (4–)5(–7)-septate, 21–30 × 5–9 µm; pantropical. *Polymeridium jordanii* (C.W. Dodge) Aptroot
W48b Ascospores 7–11-septate, (24–)28–39 × 6–9 µm; pantropical. *Polymeridium albocinereum* (Kremp.) R.C. Harris

W49a Hamathecium inspersed, ascospores 18–20 × 6.0–7.5 µm; Philippines. *Polymeridium submuriforme* Aptroot
W49b Hamathecium clear, ascospores 25–45 × 9–13 µm. 50

W50a Ascospores 30–45 × 9–11 µm, submuriform, 3–4 times as long as broad; neotropical. *Polymeridium cinereonigricans* (Vain.) R.C. Harris
W50b Ascospores 25–29 × 11–13 µm, muriform, 2.0–2.5 times as long as broad; Brazil. *Polymeridium julelloides* E.L. Lima, M. Cáceres & Aptroot

Key X. *Pseudobogoriella* species

X1a Ostiole lateral; Australia. *Pseudobogoriella lateralis* (Sipman) Lücking, R. Miranda & Aptroot
X1b Ostiole apical 2

X2a Ascospores 24–28 µm long; pantropical. *Pseudobogoriella hemisphaerica* (Müll. Arg.) Lücking, R. Miranda & Aptroot
X2b Ascospores 12–22 µm long. 3

X3a Thallus foliicolous, subcuticular, distinctly lobed; New Zealand. *Pseudobogoriella striguloides* (Sérus. & Aptroot) Lücking, R. Miranda & Aptroot
X3b Thallus corticolous, not lobed. 4

X4a Excipulum with K+ red, dissolving pigment; pantropical. *Pseudobogoriella leuckertii* (D. Hawksw. & J.C. David) Lücking, R. Miranda & Aptroot
X4b Excipulum lacking pigment. 5

ISSN 1868-4173

X5a Black prothallus line with numerous pycnidia present; pantropical. *Pseudobogoriella punctata* (Aptroot) Lücking, R. Miranda & Aptroot
X5b Black prothallus line lacking pycnidia or absent. 6

X6a Thallus besides ascomata with numerous black dots representing pycnidia. 7
X6b Thallus lacking pycnidia. 8

X7a Ascomata aggregate; ascospores 15–20 × 6–9 µm; neotropical. *Pseudobogoriella socialis* (Zahlbr.) Lücking, R. Miranda & Aptroot
X7b Ascomata solitary; ascospores 11–17 × 5–7 µm; pantropical. *Pseudobogoriella fumosula* (Zahlbr.) Lücking, R. Miranda & Aptroot

X8a Involucrellum broadly expanding basally to over 0.5 mm diam; pantropical. *Pseudobogoriella alata* (Groenh. ex Aptroot) Lücking, R. Miranda & Aptroot
X8b Ascomata including basally slightly expanding involucrellum 0.2–0.5 mm diam. 9

X9a Ascospores 12–16 µm long. 10
X9b Ascospores 16–22 µm long. 12

X10a Ascomata 03–05 mm diam; pantropical. *Pseudobogoriella subfallens* (Müll. Arg.) Lücking, R. Miranda & Aptroot
X10b Ascomata 02–03 mm diam. 11

X11a Ascospores 4–55 µm broad, 25–3 times as long as broad; involucrellum basally not expanding; pantropical. *Pseudobogoriella exigua* (Müll. Arg.) Lücking, R. Miranda & Aptroot
X11b Ascospores 55–7 µm broad, 2–25 times as long as broad; involucrellum basally expanding; China. *Pseudobogoriella minutula* (Zahlbr.) Lücking, R. Miranda & Aptroot

X12a Black prothallus line present. 13
X12b Black prothallus line absent. 14

X13a Ascomata conical, basally not expanded; neotropical. *Pseudobogoriella captiosa* (Müll. Arg.) Lücking, R. Miranda & Aptroot
X13b Ascomata fattened, basally expanded; pantropical. *Pseudobogoriella miculiformis* (Müll. Arg.) Lücking, R. Miranda & Aptroot

X14a Ascomata basally broadly expanded, fringe 50–100 µm
Broad; pantropical. *Pseudobogoriella annonacea* (Müll. Arg.) Lücking, R. Miranda & Aptroot
X14b Ascomata basally narrowly expanded, fringe 20–50 µm

Broad; pantropical. *Pseudobogoriella nonensis* (Stirt.) Lücking, R. Miranda & Aptroot

Key Y. *Pseudopyrenula* species

Y1a Thallus UV+ yellow, with lichexanthone. 2
Y1bThallus UV−. 6

Y2a Hamathecium clear; ascospores 30–45 × 8–11 µm; Venezuela. *Pseudopyrenula cryptotheca* Komposch, Aptroot & Hafellner
Y2b Hamathecium inspersed with hyaline or yellow oil droplets; ascospores under 37 µm long. 3

Y3a Hamathecium inspersed with yellow oil droplets. 4
Y3b Hamathecium inspersed with hyaline oil droplets. 5

Y4a Ascospores 21–28 × 6–9 µm; neotropical. *Pseudopyrenula flavoreagens* Aptroot & M. Cáceres
Y4b Ascospores 26–36 × 7–10 µm; Bolivia. *Pseudopyrenula flavosuperans* Flakus & Aptroot

Y5a Ascospores 21–25 × 6–9 µm; neotropical. *Pseudopyrenula guianensis* Aptroot
Y5b Ascospores 30–35 × 9–11 µm; neotropical. *Pseudopyrenula superans* Müll. Arg.

Y6a Ascospores 5-septate, 16–21 × 6–7 µm; Venezuela. *Pseudopyrenula hexamera* Aptroot
Y6b Ascospores 3-septate. 7

Y7a Thallus corticate; ascospores 21–25 × 6–9 µm; Costa Rica. *Pseudopyrenula thallina* Lücking & Aptroot
Y7b Thallus not corticate. 8

Y8a Hamathecium inspersed with hyaline or yellow oil droplets. 9
Y8b Hamathecium clear (but ascospores may contain yellow oil). 19

Y9a Hamathecium inspersed with yellow oil droplets. 10
Y9b Hamathecium inspersed with hyaline oil droplets (but ascospores may contain yellow oil). 14

Y10a Ascospores 37–44 × 10–12 µm; neotropical. *Pseudopyrenula endoxantha* (Vain.) Zahlbr.
Y10b Ascospores under 35 × 10 µm. 11

Y11a Ascospores 25–30 × 7.5–8.5 µm; pantropical. *Pseudopyrenula media* Aptroot & Diederich
Y11b Ascospores 13–25 × 5.5–9 µm. 12

Y12a Ascospores 13–17 × 5.5–6.5 µm; Brazil. *Pseudopyrenula miniflavida* Aptroot & A.D. Nunes
Y12b Ascospores 18–25 × 6–9 µm. 13

Y13a Ascospores 21–25 × 6–9 µm; hamathecium K+ deep yellow to reddish; pantropical. *Pseudopyrenula subgregaria* Müll. Arg.
Y13b Ascospores 18–22 × 6–8 µm; hamathecium K+ weakly purplish; Thailand. *Pseudopyrenula endoxanthoides* Vain.

Y14a Ascoma wall with yellow, K+ red pigment; ascospores 21–32 × 7–10 µm; Brazil. *Pseudopyrenula cerei* Vain.
Y14b Ascoma wall without yellow pigment. 15

Y15a Thallus saxicolous; ascospores 35–45 × 11–13 µm; Brazil. *Pseudopyrenula saxicola* Malme
Y15b Thallus corticolous; ascospores under 38 µm long. 16

Y16a Ascospores 33–37 × 10–12 µm; neotropical. *Pseudopyrenula dubia* Vain.
Y16b Ascospores under 33 µm long. 17

Y17a Ascospores (24–)26–32 × 7–10 µm; pantropical. *Pseudopyrenula diluta* (Fée) Müll. Arg.
Y17b Ascospores 21–25 × 6–9 µm. 18

Y18a Ostioles eccentric; Cuba. *Pseudopyrenula cubana* (Müll. Arg.) Aptroot & Lücking
Y18b Ostioles apical; pantropical. *Pseudopyrenula subnudata* Müll. Arg.

Y19a Thallus without algae, brownish, of bark colour. 20
Y19b Thallus with algae, whitish. 22

Y20a Ascospores 45–55 × 12–15 µm; USA (Florida). *Pseudopyrenula staphyleae* (Petr.) Aptroot
Y20b Ascospores under 40 µm long. 21

Y21a Ascospores 25–29 × 7–9 µm; Papua New Guinea. *Pseudopyrenula papuana* Aptroot

Y21b Ascospores 30–39 × 6–8 µm; Papua New Guinea. *Pseudopyrenula serusiauxii* Aptroot

Y22a Ascospores 12–18 × 4–6 µm; Tasmania. *Pseudopyrenula kantvilasii* (P.M. McCarthy) Aptroot, comb. nov. Mycobank no 841169; basionym: *Lithothelium kantvilasii* P.M. McCarthy, Telopea 18: 168, 2015. The description and illustration show several characters that are characteristic of the genus *Pseudopyrenula*, especially the presence of yellow oil in the ascospores, which is unknown from any other genus, but also the diamond-shaped lumina and the branched hamathecium filaments.
Y22b Ascospores 26–35 × 7–11 µm. 23

Y23a Ascospores 26–32 × 7–10 µm; neotropical. *Pseudopyrenula americana* Aptroot
Y23b Ascospores 30–35 × 9–11 µm; Brazil. *Pseudopyrenula gelatinosa* Aptroot

Key Z. *Trypethelium* species

Z1a Thallus, pseudostroma and/or ostioles UV+ yellow, with lichexanthone 2
Z1b Thallus, pseudostroma and ostioles UV−. 9

Z2a Thallus UV−, only pseudostroma and/or ostioles UV+ yellow 3
Z2b Thallus and sometimes also pseudostroma UV+ yellow 4

Z3a Pseudostroma UV+ yellow outside, with yellow pigment inside. *Trypethelium aureornatum* Aptroot
Z3b Only ostioles UV+ yellow; Brazil. *Trypethelium xanthostiolornatum* Aptroot

Z4a Hamathecium not inspersed. 5
Z4b Hamathecium inspersed. 8

Z5a Pseudostromata externally with orange pigment; Bolivia. *Trypethelium xanthoplatystomum* Flakus & Aptroot
Z5b Pseudostromata internally with yellow pigment. 6

Z6a Thallus and pseudostroma UV+ yellow; neotropical. *Trypethelium tolimense* Lücking, Moncada & M. Gut.
Z6b Only thallus UV+ yellow; pseudostroma UV−. 7

Z7a Ascospores muriform; Brazil. *Trypethelium muriforme* Aptroot & M.F. Souza
Z7b Ascospores only transversely septate; Brazil. *Trypethelium endoflavum* Aptroot

Z8a Pseudostromata lacking pigment, prominent; ascospores (65–)83–97 × 11–15 µm,13–19-septate; *Brazil*. *Trypethelium regnellii* Malme
Z8b Pseudostromata internally with yellow pigment; neotropical. *Trypethelium tolimense* Lücking, B. Mocada & M.C. Gut.

Z9a Pseudostromata inside and/or outside with yellow or orange pigments (sometimes thin and not readily visible). 10
Z9b Pseudostromata lacking pigments. 15

Z10a Ostiolar areas very broad, disc-shaped or fused in lobate fashion; pigments usually sparse. 11
Z10b Ostiolar areas narrow, visible as small dots; pigments conspicuous 13

Z11a Ostiolar areas disc-shaped, uniformly brown-black, marginally with small, black papillae arranged in circular fashion. *Trypethelium krempelhuberi* Makhija & Patw.
Z11b Ostiolar areas fused in lobate fashion, contrasting with a bordering whitish rim, without marginal papillae. 12

Z12a Ostiolar area centrally with dark red pigment in addition to orange pigment bordering the ostiolar lobes; ostioles sometimes eccentric and fused; ascospores 65–100 µm long; neotropical. *Trypethelium astroideum* Flakus & Aptroot
Z12b Ostiolar area only with bordering orange pigment; ostioles always apical; ascospores 55–75 µm long; pantropical. *Trypethelium platystomum* Mont.

Z13a Pseudostromata immersed to erumpent; ascospores 37–42 µm long, 7–9-septate; Vietnam. *Trypethelium infraeluteriae* Aptroot & Gueidan
Z13b Pseudostromata prominent to sessile; ascospores (37–)42–85 µm long, 9–17-septate. 14

Z14a Pseudostromata inside and/or outside with yellow pigment; ascospores 37–52 × 8–11 µm, 9–13-septate; pantropical. *Trypethelium eluteriae* Spreng.
Z14b Pseudostromata inside and/or outside with orange-yellow pigment; ascospores 60–85 × 11–12 µm, 13–16-septate; pantropical. *Trypethelium subeluteriae* Makhija & Patw.

Z15a Pseudostromata immersed-erumpent, with ascomata clearly distinguished and individually protruding, brown-black. 16
Z15b Pseudostromata erumpent to prominent, with ascomata completely immersed in the pseudostromata and not individually protruding, with whitish ostiolar spot or ostiolar area surrounded by irregular whitish to cream-coloured rim or pseudostromata entirely whitish. 17

Z16a Thallus verrucose-rugose; individual ascomata with flattened top; ascospores 45–55 × 6–8 µm, 12–17-septate; palaeotropical. *Trypethelium plicatorimosum* Makhija & Patw.
Z16b Thallus smooth to uneven; individual ascomata with convex top; ascospores 21–40 × 9–15 µm, 5–11-septate; pantropical. *Trypethelium inaequale* Fée

Z17a Pseudostromata erumpent to prominent, entirely white or cream-coloured; hamathecium clear. 18
Z17b Pseudostromata prominent to sessile, grey to black with contrasting, white ostiolar spots; hamathecium inspersed. 19

Z18a Pseudostromata entirely white, with small, dark ostiolar spots; ascospores 30–60 µm long, 5–9(–13)-septate; palaeotropical. *Trypethelium epileucodes* Nyl.
Z18b Pseudostromata cream-coloured, with larger, often angular-lobate ostiolar spots; ascospores 58–78 µm long, 9–15-septate; Brazil. *Trypethelium foveolatum* Müll. Arg.

Z19a Pseudostromata sessile with constricted base, upper part black, sharply contrasting with white ostiolar spots; ascospores 80–110 × 14–18 µm, 13–19-septate; Africa. *Trypethelium sphaerocephalum* (Vain.) Zahlbr.
Z19b Pseudostromata prominent to sessile, grey to black with contrasting, white ostiolar spots; hamathecium inspersed; neotropical. *Trypethelium ornatum* Müll. Arg.

Ω Key of . *Viridothelium* species

Ω1a Ascospores muriform; thallus and ascomata strongly contrasting in colour, with thallus brownish and ascomata white with black ostiolar areas; neotropical. *Viridothelium tricolor* Lücking, M.P. Nelsen & N. Salazar
Ω1b Ascospores transversely septate; thallus and ascomata not strongly contrasting in colour, more or less olive-green to yellowish brown with ascomata sometimes blackish. 2

Ω2a Ascospores 5- or more septate. 3
Ω2b Ascospores 3-septate. 10

Ω3a Ostioles eccentric. 4
Ω3b Ostioles apical. 6

Ω4a Ascospores 75–98 × 17–20 µm, 15–19-septate; Solomon Islands. *Viridothelium solomonense* Aptroot

Ω4b Ascospores 90–152 × 22–33 µm. 5

Ω5a Ascospores 99–152 × 26–33 µm, (9–)15–17-septate; India. *Viridothelium megaspermum* (Makhija & Patw.) Aptroot & Lücking
Ω5b Ascospores 90–100 × 22–26 µm, 15–21-septate; Fiji. *Viridothelium vonkonratii* Lücking, Naksuwankul & Lumbsch

Ω6a Hamathecium inspersed; ascospores 60–75 × 12–17 µm, 12–14-septate; Papua New Guinea. *Viridothelium inspersum* Aptroot
Ω6b Hamathecium clear. 7

Ω7a Ascospores 90–150 µm long. 8
Ω7b Ascospores 38–80 µm long. 9

Ω8a Ascospores 90–105 × 12–16 µm, (9–)13–17-septate; eastern palaeotropical. *Viridothelium indutum* (Stirt.) Aptroot & Lücking
Ω8b Ascospores 100–150 × 18–23 µm, 17–25-septate; Malaysia. *Viridothelium kinabaluense* Aptroot

Ω9a Ascospores 38–52 × 7–10 µm; temperate America and Asia. *Viridothelium virens* (Tuck. ex Michener) Lücking, M.P. Nelsen & Aptroot
Ω9b Ascospores 50–80 × 8–15 µm; temperate Asia. *Viridothelium cinereoglaucescens* (Vain.) Lücking, M.P. Nelsen & Aptroot

Ω10a Thallus and ascomata thinly furnished with orange pigment; Brazil. *Viridothelium leptoseptatum* Aptroot & M. Cáceres
Ω10b Thallus and ascomata lacking pigment. 11

Ω11a Ascomata mostly single, immersed to erumpent; Brazil. *Viridothelium sinuosogelatinosum* Aptroot & M. Cáceres
Ω11b Ascomata in dense, raised groups; Brazil. *Viridothelium ustulatum* M. Cáceres & Aptroot
